# Dynamic formation of single-atom catalytic active sites on ceria-supported gold nanoparticles

**DOI:** 10.1038/ncomms7511

**Published:** 2015-03-04

**Authors:** Yang-Gang Wang, Donghai Mei, Vassiliki-Alexandra Glezakou, Jun Li, Roger Rousseau

**Affiliations:** 1 Institute for Interfacial Catalysis, Pacific Northwest National Laboratory, Richland, Washington 99352, USA; 2 Department of Chemistry, Tsinghua University, Beijing 100084, China; 3 Environmental Molecular Sciences Laboratory, Pacific Northwest National Laboratory, Richland, Washington 99352, USA

## Abstract

Catalysis by gold supported on reducible oxides has been extensively studied, yet issues such as the nature of the catalytic site and the role of the reducible support remain fiercely debated topics. Here we present *ab initio* molecular dynamics simulations of an unprecedented dynamic single-atom catalytic mechanism for the oxidation of carbon monoxide by ceria-supported gold clusters. The reported dynamic single-atom catalytic mechanism results from the ability of the gold cation to strongly couple with the redox properties of the ceria in a synergistic manner, thereby lowering the energy of redox reactions. The gold cation can break away from the gold nanoparticle to catalyse carbon monoxide oxidation, adjacent to the metal/oxide interface and subsequently reintegrate back into the nanoparticle after the reaction is completed. Our study highlights the importance of the dynamic creation of active sites under reaction conditions and their essential role in catalysis.

## Abstract (web summary)

Computational investigation of heterogeneous catalytic systems is fundamentally important. Here, the authors show that, under reaction conditions, reactant-induced structural changes in ceria-supported gold nanoparticle catalysts lead to the dynamic formation of single-atom catalytic sites at the interface.

## Graphical abstract

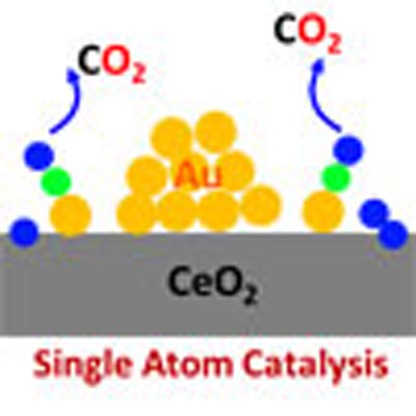

Ever since Haruta, Hutchings and others^1^,^2^,^3^ found that nanosized gold particles, supported by metal oxides, were highly reactive for a variety of important catalytic reactions, gold has been widely used as a catalyst. Extensive studies have been devoted to understanding the chemical nature of high reactivity of oxide supported gold (Au) particles^4^,^5^,^6^,^7^,^8^,^9^,^10^. Though it is generally recognized that the Au–oxide interfacial perimeter plays an important role, the chemical nature of the active sites and the details of the reaction mechanism are still hotly debated. This is partly because of the complexity of the systems, whose reactivity is affected by many factors, such as the structural and electronic properties of Au particles, the electronic interaction between the support and Au particles, the reactants and products, and finite temperature effects^4^. Nearly all of the theoretical studies on the Au/oxide system were based on carefully selected model clusters of surfaces, where the tendency of Au nanoparticles and surface to exhibit reconstruction, large amplitude motions and disorder at finite temperatures were not fully accounted for. As a prominent example, ceria (CeO_2_) has been found to be a particularly effective support for gold particles in catalysing reactions such as CO oxidation and water–gas shift reactions^9^,^10^. Generally, the high reactivity is attributed to the excellent redox properties of ceria, such as easy formation and diffusion of oxygen defects, high capacity of oxygen storage and release, and facile acceptance/release and transport of excess electrons in a polaronic state derived from a narrow 4f-band^9^,^10^. Various studies^11^,^12^,^13^,^14^ have identified the interface sites between ceria support and gold particles as vital in the catalytic reactions. Recent evidence^15^,^16^,^17^ has led to the claim that the active sites are, in actuality, positively charged single gold atoms bound to the support by O linkages. Furthermore, environmental transmission electron microscopy analyses^18^ also showed that gold nanoparticles on a CeO_2_ support change their shapes at the interfacial area in response to alternating oxidizing and reducing atmospheres. These observations highlight the necessity of understanding the nature of reactive sites on ceria-supported Au nanocatalysts, at actual reaction conditions, that is, at elevated temperature and in the presence of dynamic reacting species.

In our recent report^19^, we showed that a stable, high-symmetry, tetrahedral Au_20_ cluster on a rutile TiO_2_ support exhibited liquid-like motion on charge transfer and CO adsorption. The large adsorbate-induced structural reconstruction also allowed for facile transfer of adsorbed CO to the metal/oxide interface, where the CO_2_ formation reaction occurs. Connected with this phenomenon was the finding that charge transfer between the nanoparticle and support enabled low-energy redox reactions during the catalytic cycle. These ideas motivated us to consider how generalizable these findings are to other reducible oxides and whether other related phenomena could be involved.

In this work, we present results from density functional theory (DFT)-based *ab initio* molecular dynamics (AIMD) simulations to explore the reactivity and dynamics of ceria-supported Au catalysts for the CO oxidation reaction. Our simulations take into account both the dynamics of atoms in the Au metal particle, ceria support, reactants and products, as well as the excess charge carriers within the partially reduced ceria surface. We find that, in the presence of CO, a cationic Au^+^-CO species is created. This species migrates directly onto the ceria support and, via a strong coupling with the redox properties of the ceria, it enables low-energy barriers for redox steps in the catalytic cycle. After the catalytic cycle is complete, the Au single atom is reintegrated into the Au nanoparticle, indicating that the actual catalytic species only exists as a transient under operating conditions.

## Results

### The behaviour of Au particles on a ceria support

As is known, the geometric and electronic properties of Au particles on the oxide support strongly affect the catalytic activity of Au nanocatalyst^4^,^20^,^21^,^22^,^23^. Thus, we first performed AIMD simulations for ceria-supported Au_20_ cluster to illustrate how the finite temperature and the nature of the adsorbed species influence the morphology of gold nanoparticle, before examining the catalytic mechanism and the effect of particle size. Considering the extensive existence of oxygen vacancies on CeO_2_, we have removed one surface oxygen atom to model a partially reduced CeO_2_ support, on which we deposited the Au_20_ cluster, and allowed it to bind to the surface during a 20-ps AIMD trajectory at *T*=700 K. In a second step, we added an additional eight CO molecules on the cluster and allowed the system to further equilibrate for another 20 ps. This approach guaranteed a well-thermalized and unbiased structural model of CO adsorbed on a CeO_2_-supported Au_20_ cluster.

**Figure 1:**
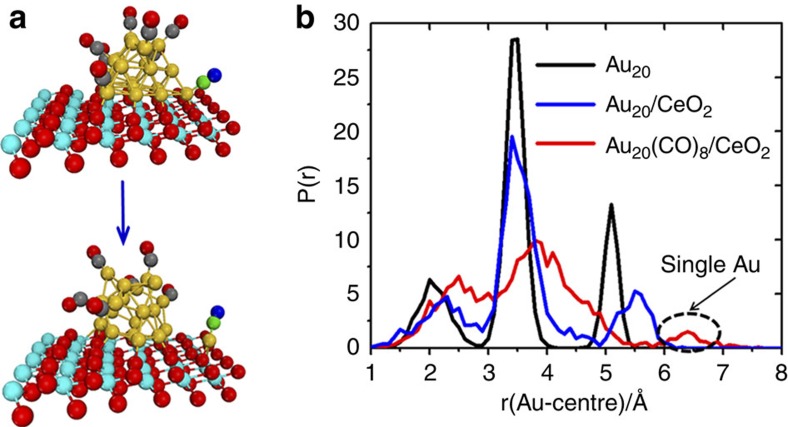
MD simulations. (**a**) Initial and final configuration of the AIMD simulation for a CeO_2_(111) supported Au_20_ cluster with eight CO adsorption. (**b**) The probability distribution functions P(r_cm_) of the Au atoms relative to the centre-of-mass of the Au_20_ based on ~20 ps MD simulations at 700 K. (Yellow sphere: Au; cyan sphere: Ce; red sphere or blue sphere: O; green sphere or grey sphere: C)

The distribution of the distance of Au atoms relative to the centre-of-mass of the Au_20_ cluster, P(r_cm_) shown in Fig. 1a, can be used to characterize the structural fluctuation within each system. For the isolated Au_20_ cluster in the gas phase, its tetrahedral configuration is retained at the simulation temperature of 700 K, and the P(r_cm_) exhibits three well-defined peaks clearly separated by zero probability density between them, corresponding to three types of Au atoms. However, when Au_20_ is supported on a CeO_2_(111) surface, Fig. 1b, it exhibits a distinct structural reconstruction and loses its tetrahedral configuration, which is manifested in P(r_cm_) as broader peaks, compared with the isolated Au_20_ cluster. The particle–surface interaction are strong due to a gold-to-oxide charge transfer, resulting in partially oxidized Au atoms at the interface as evidenced by the appearance of unpaired electrons localized in the upper layer of Ce atoms when the nanoparticle is adsorbed.

**Figure 2:**
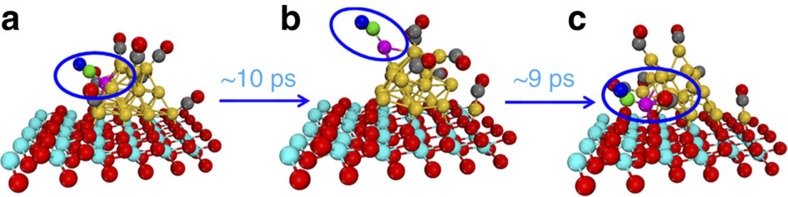
Diffusion of Au–CO unit. (**a**–**c**) Selected snapshots of the MD trajectory for Au_20_/CeO_2_ with a circled Au–CO unit to show the diffusion process. (yellow or pink sphere: Au; cyan sphere: Ce; red sphere or blue sphere: O; green sphere or grey sphere: C)

The gold nanoparticle, however, shows appreciably enhanced structural flexibility when CO is co-adsorbed. It is found that Au_20_ exhibits a strong adsorption-induced surface reconstruction and extrudes many low-coordinated sites to bind CO. As a result, the function P(r_cm_) for the Au nanoparticle exhibits significantly broader peaks with non-zero density between them. We further observed that CO diffusion proceeds in the same way as reported previously for Au/TiO_2_ (ref. 19); CO does not move freely from one Au site to another, but rather diffuses as an adsorbed Au–CO species (see Fig. 2 and Supplementary Fig. 1). Since CO oxidation is generally reported to happen at the gold-oxide interface, this finding is vital for understanding how the adsorbed CO species is transported to the perimeter site (Fig. 2a–c). These results indicate that the Au nanoparticle is rather flexible in geometry and can change its morphology on CO adsorption, consistent with previous theoretical studies^19^,^24^,^25^,^26^. However, the ceria-supported system exhibits one unique feature. As shown in Fig. 1, an Au–CO species migrates away from the gold nanoparticle directly onto the ceria support adjacent to the metal/oxide interfacial area. In the function P(r_cm_) for Au_20_, the isolated Au atom could also be identified by a peak at around 6.4 Å that is well separated from the rest of the probability function.

**Figure 3:**
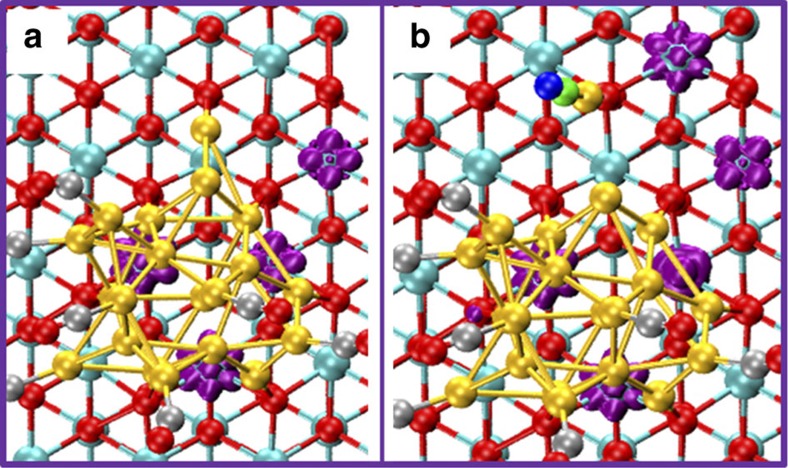
Three-dimensional isosurface of the spin densities. (**a**) Spin densities (purple areas)for the Au/CeO_2_ without an isolated Au–CO unit at the interfacial area. (**b**) Spin densities with an isolated Au–CO unit at the interfacial area. Note, the configuration in **a** is geometry-optimized configuration by removing one CO from the configuration in **b**.

To understand the nature of this specific single-Au atom species, we consider its electronic properties with and without CO adsorption. When CO is removed from the isolated Au–CO, the Au atom is readily reintegrated into the Au nanoparticle (see Fig. 3a), even in the course of a simple local geometry optimization. By monitoring the spin density, we observed four excess electrons in the CeO_2_ lattice, two more than the number that an oxygen defect can create, indicating that the Au nanoparticle transfers additional charges to the surface^27^. Once the CO is adsorbed at the Au atom, and the Au–CO moiety becomes isolated from the rest of the Au nanoparticle, an additional excess electron becomes localized on an adjacent Ce site in the topmost layer of the support. Concurrently, the charge of this Au atom increases from +0.10 |e| to +0.42 |e| This indicates that by moving to the oxygen site, the single Au atom transfers one electron to the support, resulting in a species that can be formally described as an adsorbed Au^+^–CO. Our results are consistent with previous findings^28^,^29^ that the single Au atom on CeO_2_ surface can transfer its 6s electron to the unoccupied 4f orbital of Ce. We note that the observed excess electrons are mobile on the AIMD timescale, showing hops between Ce sites in the topmost surface layer on the order of 2 ps, in accord with our previous work on partially reduced ceria^30^.

owing to the nature of ligand-to-metal electron donation of the Au–CO bond, CO is expected to energetically favour binding at the positively charge Au site. To understand the formation of single Au atom, we further considered the formation energies of the single Au atom with or without CO adsorption:


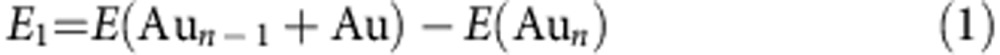



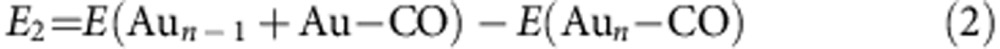


where *E*_1_ is the energy change by taking one Au atom from the Au_*n*_ nanoparticle to the next-nearest oxygen site on ceria surface and *E*_2_ is the energy associated with taking one Au–CO unit from the Au_*n*_ nanoparticle to the next-nearest oxygen site. Since in our AIMD simulations CO adsorption can only cause a low-coordinated Au atom to be isolated from the Au nanoparticle at the interface, we only consider a single four-coordinated Au site at the interface. For the Au_20_ cluster on ceria surface, the calculated *E*_1_ is 1.21 eV, indicating that for the bare Au nanoparticle it is energetically unfavourable for the Au atom to break away from the Au nanoparticle. While the calculated *E*_2_ is −0.81 eV, it indicates that forming an isolated Au^+^–CO could be thermodynamically favourable. Our work is consistent with the recent findings^31^ that CO adsorption on a CeO_2_-supported Au_4_ cluster leads to the formation of Au–CO species. Considering the CO oxidation on CeO_2_-supported Au nanoparticles is reported to proceed at room temperature^4^,^32^, we further considered the possible reaction paths for Au–CO dissociation into the adjacent oxygen site on Au_20_/CeO_2_ using climbing image nudged-elastic-band method (see Supplementary Fig. 2). The barrier is estimated to be ~0.20–0.62 eV, which indicates that the isolated Au–CO can easily be form at room temperature. Combined with the fact that Au–CO species on the Au cluster are mobile (Fig. 2), we also conclude that even if CO does not initially adsorb directly at the interface site, the isolated Au^+^–CO will eventually form.

Inasmuch as in our previous simulation^19^ we did not observe any isolated Au–CO on TiO_2_ support, we checked the single-atom formation energies on Au_20_/TiO_2_ system. The isolated Au atom without adsorbed CO is not stable at the oxygen site and slips to the adjacent five-coordinated Ti site spontaneously during the geometry optimization, in accord with Metiu’s recent report^33^,^34^. However, its formation energies are positive (*E*_2_=1.25 eV at the oxygen site; *E*_1_=0.96 eV and *E*_2_=0.90 eV at the Ti site), implying that stabilizing the oxidized Au^+^–CO on a TiO_2_ support is unfavourable. This is traced back to the fact that rutile TiO_2_ is less capable of accepting an electron from an Au–CO to stabilize a cationic species and highlights the point that the CeO_2_ support, as a stronger oxidizer, can.

Generally, the reactivity for an oxidation reaction is strongly affected by the electronic chemical potential (work function, *W*) of the catalyst. We found that creation of the isolated Au^+^–CO increased *W* from 4.8 to 5.1 eV, which indicates that the Ceria is more likely to take electrons by becoming a stronger oxidizing agent, and it is itself more easily reduced. This implies that the single Au atom at the interfacial area creates an activation centre for oxidation processes and, thus, is expected to be more reactive, in accord with recent reports on highly reactive single-atom catalysts on metal oxide^35^,^36^. In the next section, we further consider the possible reaction mechanism for CO oxidation at the isolated Au species on CeO_2_ surface and directly compare with the process at the cluster/oxide interface

### Reaction mechanism for CO oxidation

Recent theoretical studies have proposed^9^,^14^,^37^ three CO oxidation mechanisms on CeO_2_-supported Au particles: (1) CO oxidation by co-adsorbed O_2_ on Au particles, (2) CO oxidation by adsorbed O_2_ at the interfacial site and (3) CO oxidation by a lattice oxygen ion from CeO_2_ substrate. In the present study, we examined a large number of adsorption configurations of O_2_ on Au_20_ cluster or interfacial sites, but we found that the O_2_ hardly binds at these sites (*E*_ads_<−0.2 eV). These results suggest that CO oxidation with preadsorbed O_2_ at the Au nanoparticle or the interface sites are less likely to be operative for larger Au particles, and lower reduction levels of the support, as is expected to be the case for a reaction that is run under oxidizing conditions. Therefore, we consider the reaction of CO with the lattice oxygen ion, in accord with extensive experimental studies^18^,^38^,^39^,^40^ that strongly indicate that CO oxidation proceeds via a Mars-van Krevelen mechanism (that is, a redox) mechanism.

We first considered the oxidation of CO that preadsorbs on the gold nanoparticle, comparable with previous theoretical studies^14^. The detailed pathways and energetics are shown in Supplementary Fig. 3. We found that CO easily reacts with the lattice oxygen ion, and forms a bent CO_2_^−^ intermediate. This is a highly exothermic process (Δ*E*=−0.94 eV) with a low-energy barrier (Δ*E*^†^=0.25 eV). However, the subsequent CO_2_ desorption is quite unfavourable, with a desorption energy of 1.29 eV. As the lattice oxygen ion is being extracted, there is a concurrent reduction of the surface that leaves two excess electrons localized on the Ce 4f states. After the formation of an oxygen defect, O_2_ adsorption from the gas phase at this site is highly favourable in energy and the subsequent oxidization of the second CO to CO_2_ proceeds with a low-energy barrier (Δ*E*^†^=0.45 eV). Overall, the rate determining step for the oxidation of CO preadsorbing on the gold particle involves desorption of the first CO_2_ with a high-enough energy barrier such that the reaction would not be anticipated to proceed at room temperature. However, the result is in direct contradiction with the experimental findings^32^,^41^ that show the reaction of CO oxidation with lattice oxygen ion in the interfacial area at the room temperature. In short, for the current Au_20_ system on a mildly reduced ceria support, none of the three previously proposed routes corroborate this experimental observation.

**Figure 4:**
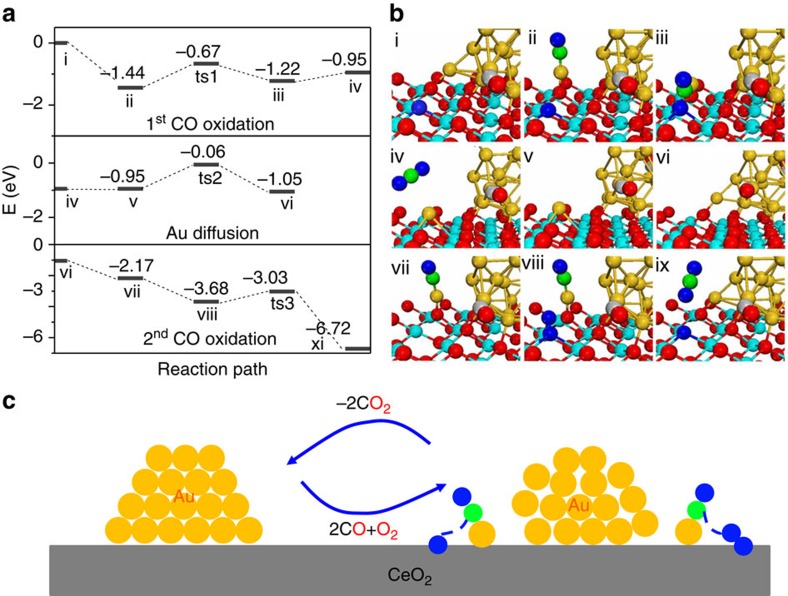
Proposed reaction mechanism for CO oxidation at single Au site. (**a**) Reaction pathways, (**b**) configurations of reactants, intermediates and products, and (**c**) schematic representation of the dynamic behavior at the interface. The catalytic processes proceed as the following steps: (1) i–ii, CO adsorption leads to the formation of isolated Au–CO; (2) ii–ts1–iii, CO preadsorbing at the single Au site reacts with the lattice oxygen ion, forming a bent intermediate; (3) iii–iv–v, CO_2_ desorbs into the gas phase and at the same time the Au atom fills into the oxygen defect; (4) v–ts2–vi, the Au atom at the oxygen defect moves back to the Au cluster; (5) vi–vii, the second CO adsorption leads to the formation of isolated Au–CO; (6) vii–viii, the O_2_ adsorption at the oxygen defect. (7) viii–ts3–xi, the second CO reacts with the adsorbed O_2_ species, forming a CO_2_ molecule. At the same time, the Au returns the Au nanoparticle, completing the catalytic cycle. (The configurations for transition states are shown in Supplementary Fig. 4. Yellow sphere: Au; cyan sphere: Ce; red sphere or blue sphere: O; green sphere: C.

As the adsorption of CO at the perimeter sites could lead to the formation of an Au^+^–CO near the interfacial area, we further consider the reaction mechanism for CO oxidation at this specific isolated site. The reaction pathway and associated energetics are shown in Fig. 4. For convenience of discussion, we also track the Bader charges of the isolated Au and the number of Ce^3+^ throughout the catalytic process for each stationary point on the potential energy surface, shown in Table 1. In the proposed mechanism, we start the reaction process from the Au nanoparticle without an isolated Au site (Configuration i in Fig. 4). First, the adsorption of CO at the low-coordinate site leads to the formation of an isolated Au^+^–CO unit (Configuration ii). By overcoming a barrier of 0.77 eV, the CO can react with the adjacent oxygen ion, forming a bent CO_2_^−^ intermediate (Configuration iii). Further, this bent intermediate only requires 0.27 eV of energy to release the CO_2_ molecule into the gas phase (Configuration iv). In this process, the single Au atom migrates towards and occupies the oxygen defect after CO_2_ desorption. The Bader charge of the Au atom significantly decreases from +0.40 to −0.56, while the number of Ce^3+^ ions remains unchanged. These results indicate that in tandem with the removal of the lattice oxygen ion, the two resulting excess electrons are transferred to the single Au atom, leading to a conversion of the oxidation state from Au^+^ to a Au^−^ that fills the oxygen vacancy. In other words, this single atom stabilizes the reduction of the ceria surface leading to more facile CO_2_ desorption. This observation is consistent with our previous analysis of work function, which suggests in the presence of a charged Au^+^+CO, Ceria is locally a stronger oxidizing agent. However, the resulting Au^−^ species, which fills the oxygen vacancy, is in direct contrast with the CO oxidation on the Au nanoparticle where the two resulting excess electrons become localized at two Ce^3+^ ions.

To complete the catalytic cycle, we consider the second half of the CO oxidation reaction. Once the single Au atom has moved into the oxygen defect, it is harder to bind CO (*E*_ads_=−0.16 eV) or O_2_ (*E*_ads_=−0.10 eV) molecules. At first glance, this would indicate that the catalyst is deactivated for further CO oxidation, which is consistent with a recent study^17^ showing that Au at the oxygen defect has no further reactivity for CO oxidation. However, further CO oxidation can be achieved by extracting the Au atom out of the vacancy back to the Au cluster, so that it could bind the second CO while the vacancy becomes available for O_2_ adsorption. We noted that our AIMD simulations for Au_20_/CeO_2_ with one oxygen vacancy did not show any Au atom permanently occupying the oxygen vacancy, which implies that it may also be possible that the single Au atom may be able to reintegrate with the Au cluster (Configuration vi). With this in mind, we further performed CI-NEB calculations, as well as, constrained AIMD computation of a potential of mean force for the single Au reintegrating into the Au cluster without the presence of CO. We found that the single Au atom, at the oxygen vacancy, can move up and reintegrate within into Au cluster with a barrier of 0.89 eV on the potential energy surface and 0.81 eV on the free energy surface. This process is slightly exothermic with Au preferring energetically to be reintegrating into the Au cluster by −0.10 eV. Note these relatively low barriers are consistent with room temperature activity. Once the Au is back to the Au cluster, the second CO can form another stable Au^+^–CO species near the interfacial area (Configuration vii), by exothermic adsorption energy of −1.12 eV. During these processes, the Bader charge of the single Au atom increases from −0.54 to 0.40 *e*^*−*^. The surface accumulates two more Ce^3+^ ions, indicating that by moving out of the oxygen defect, the Au ion returns two excess electrons to the lattice. This result implies that the driving force for the Au moving out of the oxygen defect comes from the attraction of the Au cluster, and it also explains the recent experimental findings^42^,^43^ that the clustering of Au from an isolated Au precursor results in an increase of the CO oxidation activity. Recent theoretical studies^17^,^31^ also showed that the small Au_*n*_ cluster (Au_2_ or Au_3_) are catalytically reactive for CO oxidation without filling one Au into the oxygen defect site. Since the oxygen vacancy is recreated, the O_2_ molecule in the gas phase could easily fill into the oxygen defect with a high binding energy of −1.51 eV, eliminating the two excess electrons and forming a peroxo O_2_^2−^ species (Configuration viii). As a result, the CO at the single Au site only needs to overcome a small barrier of 0.65 eV to react with the peroxo O_2_^2−^ species, releasing the second CO_2_ (Configuration ix). During this process, the Au atom gradually loses its adsorbed CO and returns to the Au nanoparticle, completing the catalytic cycle. Overall, the proposed mechanism suggests that the rate-limiting step for CO oxidation is the removal of the single Au out of the oxygen vacancy. The barrier of the rate-limiting step at the single atom site is 0.89 eV, which is far lower than that at the gold particles (1.29 eV), suggesting that the transient monatomic species are the active sites in CeO_2_-supported Au catalyst.

For a direct comparison with the experimental observation for the room temperature CO oxidation^32^,^41^, we made a simple estimation of the reaction rate in the form of an attempt frequency multiplied by a Boltzmann factor, that is, *ν*=*ν*_0_exp(−*E*_*a*_/*k*_B_*T*) and assuming *ν*_0_=10^13^ s^−1^, a reasonable value for most oxides as suggested by Marrochelli and Yildiz^44^. From this formula, the range of temperatures at which the reaction process could occur, within a minimum range of rate constant 0.01~1 s^−1^, are estimated to be 300–340 K, consistent with room temperature activity. We also performed constrained MD simulations to obtain the free energy barrier at 300 K for the migration of the Au atom towards the Au cluster (Supplementary Fig. 5). This simulation allows for the sampling of collective motions, such as the restructuring of the entire Au particle on reintegration of the Au ad-atom, and thus would be expected to better account for both the energy and entropic components of the process. The free energy barrier is estimated to be 0.81 eV and based on the transition state theory formalism *ν*=*k*_B_*T*/*h* exp(−Δ*G*^†^/*k*_B_*T*) the rate constant at 300 K is estimated to be 0.1 s^−1^, indicating that the process could occur several times per minute at room temperature.

Above, we proposed that the Au cluster could drive the Au atom out of the vacancy. We also noted that an isolated Au atom at the oxygen defect could also be directly pulled out of the oxygen defect by an adsorbed CO, with a barrier of 0.85 eV, and a highly exothermic reaction energy of −1.06 eV. Since the initially CO adsorption is very weak (only −0.16 eV), this process will resemble an Eley–Rideal mechanism (the energy barrier is 0.85–0.16=0.69 eV) with a relatively low rate at reaction conditions where CO oxidation is typically performed. However, it may still happen under CO-rich conditions, where the high partial pressure of CO would significantly increase the sticking possibility of CO at the single Au site, and would enhance the reaction probability for the second CO oxidation step without changing the subsequent steps in reaction mechanism. In addition, recent theoretical studies by Song *et al*.^45^,^46^ also proposed a relevant mechanism for CO oxidation and propylene oxidation at an isolated Au site, which are compatible with our findings.

Since the single Au atom is induced by CO adsorption at the interfacial area, it is its mobility that ultimately determines where the CO oxidation proceeds. In Supplementary Fig. 6, we show the reaction paths and energetics for Au diffusion to another oxygen site away from the Au nanoparticle. With the adsorption of CO, the single Au atom has to overcome a high barrier of 1.14 eV to diffuse from the interface to the next oxygen site, indicating that the diffusion of Au–CO unit on the ceria surface is improbable at ambient conditions. As the barrier for CO reacting with the lattice oxygen is smaller (0.77 eV) than the diffusion barrier, the Au^+^–CO is expected to oxidize appreciably faster than it can diffuse. We further consider a single Au atom away from the Au nanoparticle without CO adsorption. Similarly, the barrier for the diffusion away from the interface is much higher (1.59 eV). Especially, when considering the inverse diffusion, the Au atom at the oxygen site only needs to overcome a very low barrier of 0.37 eV to diffuse back towards the Au nanoparticle. The process is highly exothermic with a reaction energy of −1.22 eV and is the result of the above-mentioned propensity for the Au atoms to reintegrate with the nanoparticle when CO is not present. Taken together, these results demonstrate that single Au atom catalysis for CO oxidation would occur predominantly at the interfacial area.

### Nanoparticle size effects

**Figure 5:**
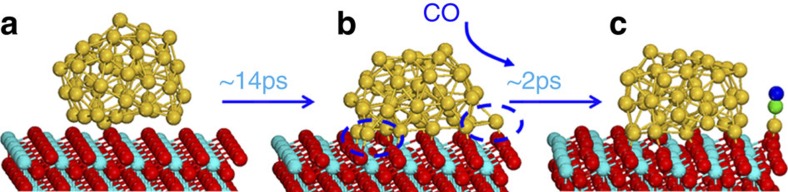
Snapshots of MD simulation for CeO_2_-supported Au_50_ cluster. (**a**) Initial configuration of Au_50_/CeO_2_. (**b**) the snapshot of Au_50_/CeO_2_ at ~14 ps. (**c**) the snapshot for the MD simulation with one CO initially adsorbed at the low-coordinated site. The low coordinate sites in (**b**) are circled in dotted line. See Supplementary Information for more snapshots to demonstrate the formation of low-coordinated sites frequently during the MD simulation.

Since our AIMD simulations for Au_20_/CeO_2_ only show the formation of the isolated Au^+^–CO species at the low-coordinated sites, one may ask if the formation of the isolated Au^+^–CO species is determined by the specific model cluster and how applicable are these findings to large Au particles. Therefore, we further performed AIMD simulations for a bigger Au_50_ cluster (~1.5 nm) on CeO_2_ support, which initially had no low-coordinate site at the interface area, shown in Fig. 5. We observed that the low-coordinate sites formed at the interface area, revealing that for even 1 nm Au particles, or larger, the low-coordination sites should always exist at the interface. We further considered the formation energy of the isolated Au–CO species on the CeO_2_ surface. In Table 2, we list the single-Au formation energies for Au_10_, Au_20_ and Au_50_. Without CO adsorption, the formation energies are endothermic for all three Au clusters, indicating that the creation of an isolated Au ad-atom is energetically unfavourable in all cases. Conversely, in the presence of CO, the adsorption energies at isolated Au^+^ sites for all clusters become exothermic, illustrating how formation of this species near the metal/oxide interface becomes thermodynamically favourable. We find that the above-mentioned formation energies are all within the same energy range with a variance of 0.3 eV and no discernible trend indicating that this general result is not particularly influenced by the cluster size. We further performed a short (~5 ps) AIMD simulation for CeO_2_-supported Au_10_ and Au_50,_ where CO molecules are initial located at a low-coordination interfacial Au site, to compare with our Au_20_ results. In agreement with our analysis above, we observe spontaneous formation of an Au^+^+CO atom at the interface for both cases within only a few ps, see Fig. 5c and Supplementary Fig. 7.

In summary, we find that, for both smaller and larger Au nanoparticles, the surface-adsorbed Au^+−^–CO can be formed readily by CO adsorption. We speculate that under realistic conditions, small-size Au particles, with potentially a higher ratio of low coordinate sites at the interface than the large particles, should produce more single Au atom on CO adsorption. This conclusion is also consistent with our AIMD simulations on Au_10_/CeO_2_ with four adsorbed CO molecules, where we observed two single-Au atoms at the interface (Supplementary Fig. 7).

## Discussion

In the present study, we performed static DFT calculations and *ab initio* molecular dynamic simulations to gain insight on the reaction mechanism of CO oxidation at the interfacial area. A new, dynamic, single-atom catalytic mechanism is established for CO oxidation on Au/CeO_2_ catalysts, arising from a transient Au^+^–CO species. During the reaction process, a single Au^+^ atom acts as charge acceptor for enhanced CO adsorption and transport. This species considerably lowers the barriers for CeO_2_ reduction, effectively promoting the CO oxidation reaction. However, the active species, an Au^+^–CO ion, emerges only in the presence of CO, and otherwise reintegrates with the Au nanoparticle. It is this monoatomic species that is able to couple its redox processes effectively with those of the support to enhance the redox processes, whilst the Au nanoparticle shows little evidence of coupling of its charge state with that of the oxide during the catalytic cycle.

Our study touches on a very important concept in the heterogeneous catalysis, namely that the actual catalytic active centre may be created only under reaction conditions and may not necessarily be easy to identify *ex situ*. The current study adds a layering to this concept by suggesting that, in the present case, the formation of the active site is a dynamic process occurring at the support oxide/metal particle interface. Our results promote the concept that the dynamic behaviour induced by the reactants at the interfacial area may be an overlooked aspect on the catalysis on reducible supports.

## Methods

### DFT parameters

All DFT calculations were performed using the CP2K package^47^. The exchange-correlation energy was described by the generalized-gradient approximation with spin-polarized Perdew–Burke–Ernzerh functional^48^. The wavefunctions were expanded in an optimized double-ζ Gaussian basis sets^30^,^49^ with an auxiliary plane wave basis set with a cutoff energy of 500 Rydberg^50^. Core electrons have been modelled by scalar relativistic norm-conserving pseudo potentials^51^ with 12, 6, 1 and 4 valence electrons for Ce, O, H and C, respectively. Brillouin zone integration is performed with a reciprocal space mesh consisting of only the gamma point. The DFT+U method, based on the Mullikan 4f state population analysis, was used to describe the Ce 4f electrons. A *U* value of 7.0 eV was used to reproduce the correct band gap of 3.2 eV, gap state location of 1.3 eV above the valence band, and work function of 4.6 eV for CeO_2_, which ensure that the redox chemistry is reproduced correctly. The detail tests and discussion can be found in our recent study^30^ and in the Supplementary methods. We observed many localized 4f electrons on CeO_2_-supported Au clsuter, which are mobile and can be localized at many different Ce sites in the simulations. This may lead to 0.1–0.3 eV difference for the energetics, as is discussed in our previous study^30^. The location and energy of the transition state was performed using the climbing image nudged-elastic-band method (CI-NEB)^52^,^53^ including 9 replicas. The convergence criterion for the maximum force is set as 2 × 10^−3^ atomic units. Vibrational analysis was further used to confirm the transition states corresponded to true saddle points on the potential energy surface with only one imaginary frequency.

### Computational models

The CeO_2_(111)-p(5 × 6) surface with one oxygen defect was used to model the CeO_2_ substrate, consisting of 4 O-Ce-O tri-layers (12 atomic layers), and only the bottom Ce atomic-layer were frozen while the remaining layers were allowed to relax. The slab was repeated periodically with a vacuum layer of 20 Å in the direction of the surface normal. A tetrahedral Au_20_ cluster, which has been well characterized in previous studies, was chosen to model Au particles. The benchmark test of current approach with respect to a family of Au_20_ isomers can be found in our recent study^19^. To consider the nanoparticle size effect, Au_10_ (planar) and Au_50_ cluster were chose to model the different size of gold nanoparticles (the coordinates are listed in Supplementary Data 1).

### AIMD simulations

All *ab initio* MD simulations were performed by sampling the canonical ensemble employing Nose–Hoover thermostats^54^,^55^ with a time step of 0.5 fs at a finite temperature of 300 or 700 K. On the basis of simple geometry optimization, CeO_2_-supported Au_20_ cluster is estimated to be able to bind up to 10 CO molecules and the average binding energy is 0.87 eV per CO. This result is consistent with our recent study^19^ on TiO_2_-supported Au_20_ cluster where we also found the system can at most bind 10 CO. Therefore, in our MD simulations, we considered both low coverage and high coverage of CO on the Au cluster to explore the behaviour of CO-induced reconstruction. Since the relatively short timescales of AIMD limit sampling to only very fast, low-energy barrier events and preclude the observation of slow processes, the high temperature simulations could more rapidly explore a large volume of phase space. On the basis of a simple estimation according to 10^13^_*_exp(*−E*_*a*_*/RT*), the rate constant for a reaction with an energy barrier of 0.5 eV at 700 K is around 0.0025, ps^−1^, this means that the process can happen only one time every 400 ps. Therefore, only the reaction process with the energy barrier lower than 0.5 eV may be observed in our MD simulations at 700 K due to the limitation of the timescale (10–30 ps).

## Author contributions

Y.-G.W. performed the calculations. D.M. and V.-A.G. contributed to the MD simulations. R.R., J.L. and V.-A.G. conceived the work and supervised the project. Y.-G.W., V.-A.G., R.R. and J.L. co-wrote the manuscript. All authors discussed the results and commented on the manuscript.

## Additional information

**How to cite this article:** Wang, Y.-G. *et al*. Dynamic formation of single-atom catalytic active sites on ceria-supported gold nanoparticles. *Nat. Commun.* 6:6511 doi: 10.1038/ncomms7511 (2015).

## Supplementary Material

Supplementary Figures, Supplementary Methods and Supplementary References Supplementary Figures 1-7, Supplementary Methods and Supplementary References

Supplementary Data 1 Coordinates and energies of all stationary points on the potential energy surface

## Figures and Tables

**Table 1:** The Bader charges of the single Au atom and the number of Ce^3+^ ions in the system.

	**First CO oxidation**				**Second CO oxidation**				
**Intermediate**	**i**	**ii**	**iii**	**iv**	**v**	**vi**	**vii**	**viii**	**ix**
q(Au)/*e−*	0.10	0.42	0.04	−0.56	−0.54	0.11	0.40	0.46	0.10
N(Ce^3+^)	4	5	5	5	5	6	7	5	4

**Table 2:** The formation energy of single Au atom at interfacial area.

	**Au**_**10**_	**Au**_**20**_	**Au**_**50**_
*E*_1_ (eV)	1.55	1.21	1.50
*E*_2_ (eV)	−0.50	−0.81	−0.73

## References

[b1] Haruta M., Kobayashi T., Sano H. & Yamada N. Novel gold catalysts for the oxidation of carbon monoxide at a temperature far below 0°C. Chem. Lett. 2, 405–408 (1987).

[b2] Haruta M. Size-and support-dependency in the catalysis of gold. Catal. Today 36, 153–166 (1997).

[b3] Hutchings G. J. Gold catalysis in chemical processing. Catal. Today 72, 11–17 (2002).

[b4] Hvolbæk B. . Catalytic activity of Au nanoparticles. Nano Today 2, 14–18 (2007).

[b5] Vilhelmsen L. B. & Hammer B. Identification of the catalytic site at the interface perimeter of au clusters on rutile TiO_2_(110). ACS Catal. 4, 1626–1631 (2014).

[b6] Green I. X., Tang W., Neurock M. & Yates J. T. Jr Insights into catalytic oxidation at the Au/TiO_2_ dual perimeter sites. Acc. Chem. Res. 47, 805–815 (2013). 24372536 10.1021/ar400196f

[b7] Wang J. & Hammer B. Oxidation state of oxide supported nanometric gold. Top. Catal. 44, 49–56 (2007).

[b8] Farnesi Camellone M. & Marx D. Nature and role of activated molecular oxygen species at the gold/titania interface in the selective oxidation of alcohols. J. Phys. Chem. C 118, 20989–21000 (2014).

[b9] Zhang C., Michaelides A. & Jenkins S. J. Theory of gold on ceria. Phys. Chem. Chem. Phys. 13, 22–33 (2010). 21046045 10.1039/c0cp01123a

[b10] Paier J., Penschke C. & Sauer J. Oxygen defects and surface chemistry of ceria: quantum chemical studies compared to experiment. Chem. Rev. 113, 3949–3985 (2013). 23651311 10.1021/cr3004949

[b11] Rodriguez J. A. Gold-based catalysts for the water–gas shift reaction: active sites and reaction mechanism. Catal. Today 160, 3–10 (2011).

[b12] Zhou Z., Kooi S., Flytzani-Stephanopoulos M. & Saltsburg H. The role of the interface in CO Oxidation on Au/CeO_2_ multilayer nanotowers. Adv. Funct. Mater. 18, 2801–2807 (2008).

[b13] Longo A. . Structure of the metal–support interface and oxidation state of gold nanoparticles supported on ceria. J. Phys. Chem. C 116, 2960–2966 (2011).

[b14] Kim H. Y., Lee H. M. & Henkelman G. CO oxidation mechanism on CeO_2_-Supported Au nanoparticles. J. Am. Chem. Soc. 134, 1560–1570 (2012). 22191484 10.1021/ja207510v

[b15] Guan Y. & Hensen E. Cyanide leaching of Au/CeO_2_: highly active gold clusters for 1, 3-butadiene hydrogenation. Phys. Chem. Chem. Phys. 11, 9578–9582 (2009). 19830344 10.1039/b909487c

[b16] Flytzani-Stephanopoulos M. Gold atoms stabilized on various supports catalyze the water–gas shift reaction. Acc. Chem. Res. 47, 783–792 (2013). 24266870 10.1021/ar4001845

[b17] Camellone M. F. & Fabris S. Reaction mechanisms for the CO oxidation on Au/CeO_2_ catalysts: Activity of substitutional Au^3+^/Au^+^ cations and deactivation of supported Au+ adatoms. J. Am. Chem. Soc. 131, 10473–10483 (2009). 19722624 10.1021/ja902109k

[b18] Ta N. . Stabilized gold nanoparticles on ceria nanorods by strong interfacial anchoring. J. Am. Chem. Soc. 134, 20585–20588 (2012). 23267697 10.1021/ja310341j

[b19] Wang Y.-G., Yoon Y., Glezakou V.-A., Li J. & Rousseau R. The role of reducible oxide–metal cluster charge transfer in catalytic processes: new insights on the catalytic mechanism of CO oxidation on Au/TiO_2_ from ab initio molecular dynamics. J. Am. Chem. Soc. 135, 10673–10683 (2013). 23782230 10.1021/ja402063v

[b20] Lopez N. & Nørskov J. K. Catalytic CO oxidation by a gold nanoparticle: a density functional study. J. Am. Chem. Soc. 124, 11262–11263 (2002). 12236728 10.1021/ja026998a

[b21] Yoon B. . Charging effects on bonding and catalyzed oxidation of CO on Au_8_ clusters on MgO. Science 307, 403–407 (2005). 15662008 10.1126/science.1104168

[b22] Remediakis I. N., Lopez N. & Nørskov J. K. CO oxidation on rutile‐supported Au nanoparticles. Angew. Chem. In. Ed. 117, 1858–1860 (2005). 10.1002/anie.200461699 15712248

[b23] Gates B. C. Supported gold catalysts: new properties offered by nanometer and sub-nanometer structures. Chem. Commun. 49, 7876–7877 (2013). 10.1039/c3cc44942d 23904034

[b24] Pal R. . Chemisorption-Induced 2D–3D–2D structural transitions in gold heptamer:(CO)_n_Au_7_^–^(n= 1–4). J. Phys. Chem. Lett. 2, 2288–2293 (2011).

[b25] Li W.-K., Chu L.-N., Gong X.-Q. & Lu G. A comparative DFT study of adsorption and catalytic performance of Au nanoparticles at anatase and brookite TiO_2_ surfaces. Surf. Sci. 605, 1369–1380 (2011).

[b26] Zhai H. J. . Chemisorption-induced structural changes and transition from chemisorption to physisorption in Au_6_(CO)_n_^−^(n= 4− 9). J. Phys. Chem. C 112, 11920–11928 (2008).

[b27] Zhang C., Michaelides A., King D. A. & Jenkins S. J. Positive charge states and possible polymorphism of gold nanoclusters on reduced ceria. J. Am. Chem. Soc. 132, 2175–2182 (2010). 20102238 10.1021/ja906687f

[b28] Liu Z. P., Jenkins S. J. & King D. A. Origin and activity of oxidized gold in water-gas-shift catalysis. Phys. Rev. Lett. 94, 196102 (2005). 16090190 10.1103/PhysRevLett.94.196102

[b29] Zhang C., Michaelides A., King D. A. & Jenkins S. J. Structure of gold atoms on stoichiometric and defective ceria surfaces. J. Chem. Phys. 129, 194708 (2008). 19026082 10.1063/1.3009629

[b30] Wang Y.-G., Mei D., Li J. & Rousseau R. DFT+U study on the localized electronic states and their potential role during H_2_O dissociation and CO oxidation processes on CeO_2_(111) surface. J. Phys. Chem. Lett. 4, 2256–2363 (2013).

[b31] Ghosh P., Camellone M. & Fabris S. Fluxionaality of Au clusters at ceria surfaces during CO oxidation: relationships among reactivity, size, cohesion, and surface defects from DFT simulations. J. Phys. Chem. C 117, 23082–23089 (2013).

[b32] Zhang S. . CO oxidation activity at room temperature over Au/CeO_2_ catalysts: disclosure of induction period and humidity effect. ACS Catal. 4, 3481–3489 (2014).

[b33] Chrétien S. & Metiu H. Density functional study of the interaction between small Au clusters, Aun (n= 1–7) and the rutile TiO_2_ surface. II. Adsorption on a partially reduced surface. J. Chem. Phys. 127, 244708 (2007). 18163696 10.1063/1.2806802

[b34] Chrétien S. & Metiu H. Density functional study of the charge on Aun clusters (n=1–7) supported on a partially reduced rutile TiO2 (110): are all clusters negatively charged? J. Chem. Phys. 126, 104701 (2007). 17362075 10.1063/1.2709886

[b35] Qiao B. . Single-atom catalysis of CO oxidation using Pt1/FeOx. Nat. Chem. 3, 634–641 (2011). 21778984 10.1038/nchem.1095

[b36] Yang X.-F. . Single-Atom catalysts: a new frontier in heterogeneous catalysis. Acc. Chem. Res. 46, 1740–1748 (2013). 23815772 10.1021/ar300361m

[b37] Song W. & Hensen E. J. M. A computational DFT study of CO oxidation on a Au nanorod supported on CeO_2_(110): on the role of the support termination. Catal. Sci. Technol. 3, 3020–3029 (2013).

[b38] Shapovalov V. & Metiu H. Catalysis by doped oxides: CO oxidation by Au_x_Ce_1−x_O_2_. J. Catal. 245, 205–214 (2007).

[b39] Widmann D., Leppelt R. & Behm R. Activation of a Au/CeO_2_catalyst for the CO oxidation reaction by surface oxygen removal/oxygen vacancy formation. J. Catal. 251, 437–442 (2007).

[b40] Qian K. . Influences of CeO_2_ microstructures on the structure and activity of Au/CeO_2_/SiO_2_ catalysts in CO oxidation. J. Mol. Catal. A-Chem. 306, 40–47 (2009).

[b41] Deng W., Carpenter C., Yi N. & Flytzani-Stephanopoulos M. Comparison of the activity of Au/CeO_2_ and Au/Fe_2_O_3_ catalysts for the CO oxidation and the water-gas shift reactions. Top. Catal. 44, 199–208 (2007).

[b42] Aguilar-Guerreroa V. & Gates B. C. Genesis of a highly active cerium oxide-supported gold catalyst for CO oxidation. Chem. Commun. 30, 3210–3212 (2007). 10.1039/b705562e 17653391

[b43] Aguilar-Guerrero V., Lobo-Lapidus R. J. & Gates B. C. Genesis of a cerium oxide supported gold catalyst for CO oxidation: transformation of mononuclear gold complexes into clusters as characterized by X-ray absorption spectroscopy. J. Phys. Chem. C 113, 3259–3269 (2009).

[b44] Marrocchelli D. & Yildiz B. First-principles assessment of H_2_S and H_2_O reaction mechanisms and the subsequent hydrogen absorption on the CeO2(111) surface. J. Phys. Chem. C 116, 2411–2424 (2012).

[b45] Song W. & Hensen E. J. M. Structure sensitivity in CO oxidation by a single Au atom supported on ceria. J. Phys. Chem. C 117, 7721–7726 (2013).

[b46] Song W. . Selective propylene oxidation to acrolein by gold dispersed on MgCuCr_2_O_4_ spinel. ACS. Catal. 5, 1100–1111 (2015).

[b47] VandeVondele J. . Quickstep: fast and accurate density functional calculations using a mixed Gaussian and plane waves approach. Comp. Phys. Commun. 167, 103–128 (2005).

[b48] Perdew J. P., Burke K. & Ernzerhof M. Generalized gradient approximation made simple. Phys. Rev. Lett. 77, 3865–3868 (1996). 10062328 10.1103/PhysRevLett.77.3865

[b49] VandeVondele J. & Hutter J. Gaussian basis sets for accurate calculations on molecular systems in gas and condensed phases. J. Chem. Phys. 127, 114105 (2007). 17887826 10.1063/1.2770708

[b50] Lippert B. G., Hutter J. & Parrinello M. A hybrid Gaussian and plane wave density functional scheme. Mol. Phys. 92, 477–488 (1997).

[b51] Goedecker S., Teter M. & Hutter J. Separable dual-space Gaussian pseudopotentials. Phys. Rev. B 54, 1703–1710 (1996). 10.1103/physrevb.54.1703 9986014

[b52] Mills G., Jónsson H. & Schenter G. K. Reversible work transition state theory: application to dissociative adsorption of hydrogen. Surf. Sci. 324, 305–337 (1995).

[b53] Henkelman G., Uberuaga B. P. & Jónsson H. A climbing image nudged elastic band method for finding saddle points and minimum energy paths. J. Chem. Phys. 113, 9901 (2000).

[b54] Nosé S. A unified formulation of the constant temperature molecular dynamics methods. J. Chem. Phys. 81, 511 (1984).

[b55] Hoover W. G. Canonical dynamics: equilibrium phase-space distributions. Phys. Rev. A 31, 1695–1697 (1985). 9895674 10.1103/physreva.31.1695

