# Supplementary material for: Dynamic formation of single-atom catalytic active sites on ceria-supported gold nanoparticles
Source: Nat Commun. 2015 Mar 4;6:6511. doi: 10.1038/ncomms7511 (PMC4366521; doi:10.1038/ncomms7511)
Supplement: Supplementary Figures, Supplementary Methods and Supplementary References — Supplementary Figures 1-7, Supplementary Methods and Supplementary References [file ncomms7511-s1.pdf]

Supplementary Figures

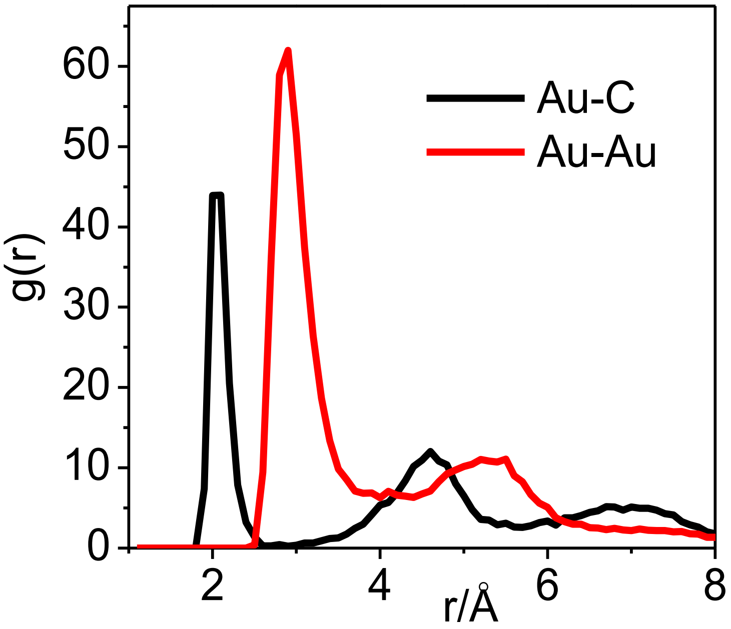

Supplementary Figure 1 | Radial bond distributions for Au-C and Au-Au bond. The zero density regime between the first two peaks in $g_{Au\text{-}C}(r)$ indicate that once a strong Au-C bond is formed, it does not break on the simulation time scale. While the appreciable density between the main broad peaks in $g_{Au\text{-}Au}(r)$ is indicative of the more liquid-like state of the Au particle. These results indicate that CO is not able to move freely from one Au site to another Au site, but it in fact diffuse easily all over the surface of gold particle by carrying along the Au atom to which it is bound. The diffusion coefficient is estimated to be 0.17 $Å^2 \bullet ps^{-1}$ at 700 K according to Einstein relationship (D=1/6<(r(t)-r(0))^2>/t). Since CO oxidation is generally reported to happen at the gold-oxide interface, this finding is very vital for understanding how the adsorbed CO species is transported to the perimeter site.

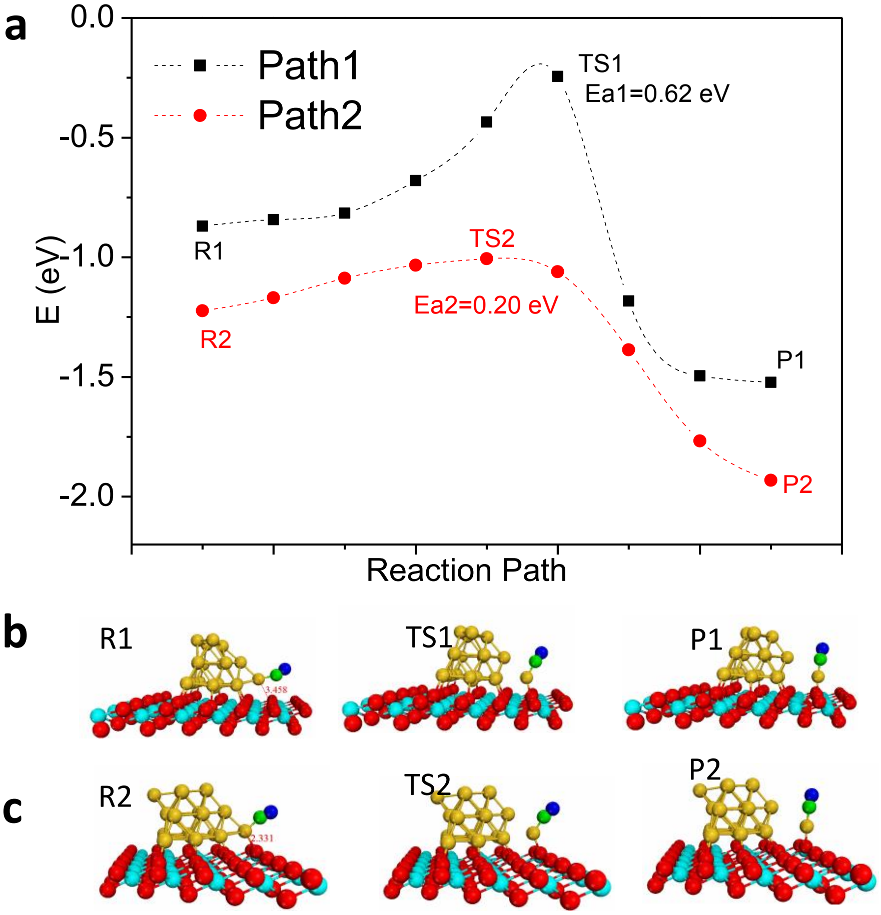

Supplementary Figure 2 | Potential energy surface for Au-CO dissociation. (a) potential energy surfaces for path 1 and path 2. (b) reactants, transition states and products for path 1. (c) reactants, transition states and products for path 2. The isolated Au^+-CO species are thermodynamically more favorable than the Au^+-CO species on the Au cluster and as a result, our AIMD simulation showed the formation of isolated Au^+-CO species. Here, we further consider the potential energy surface by using CINEB method. Based on simple geometry optimization, we found two types of CO adsorption at the interfacial Au sites depending on whether or not the Au is directly bonded to the surface oxygen. In reaction path 1, the Au^+-CO species is not initially bonded with the surface oxygen, as a result, it experience a barrier of 0.62 eV to dissociate into an isolated Au^+-CO species. In contrast, the Au^+-CO species, which is initially bonded with the surface oxygen, only needs to overcome a barrier of 0.20 eV to form an isolated Au^+-CO species. Overall, the energy barriers for dissociating the Au^+-CO species are very low that is expected to happen frequently at room temperature. (The spline line is just used to fit the points for better viewing. The value at the stationary point represents the CO adsorption energy.)

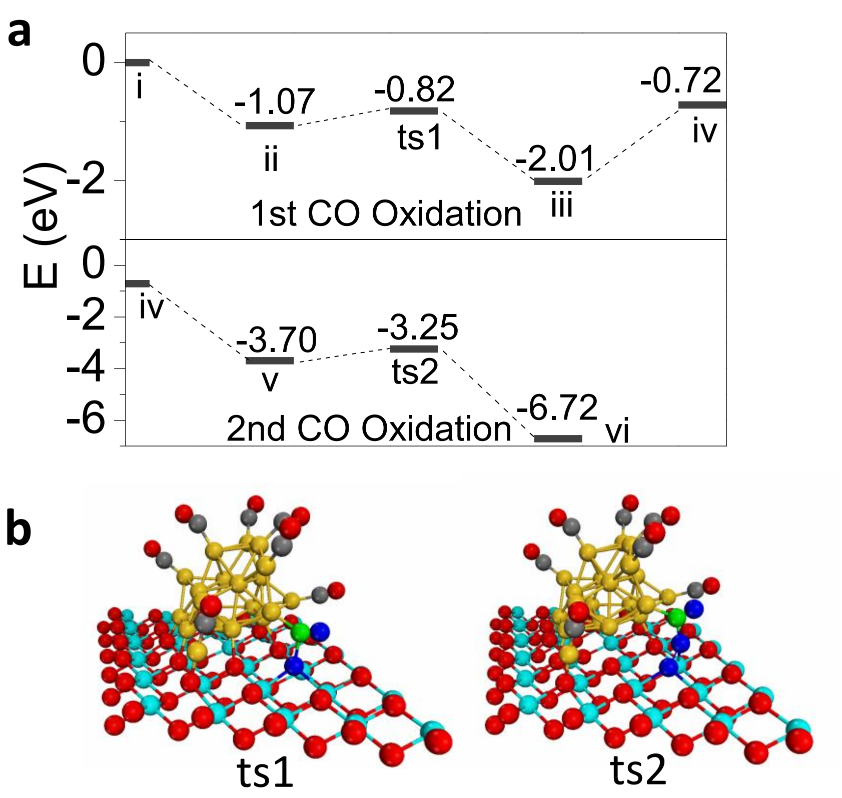

Supplementary Figure 3 | Potential energy surface for CO Oxidation on Au cluster. (a) Potential energy surface for CO oxidaion on Au cluster. (b) Configurations for transition states. The catalytic cycle proceeds as the following steps: (1) i-ii, CO adsorption; (2) ii-ts1-iii, the adsorbed CO reacts with the lattice oxygen, forming an adsorbed *CO_2 species; (3) iii-iv, CO_2 desorbs into the gas phase; (4) iv-v, CO adsorb at the Au cluster and O_2 fills the oxygen defect; (5) v-ts2-vi: CO reacts with the adsorbed O_2 species. The reaction path indicates that CO_2 desorption step is rate-limiting step and is energetically unfavorable at room temperature.

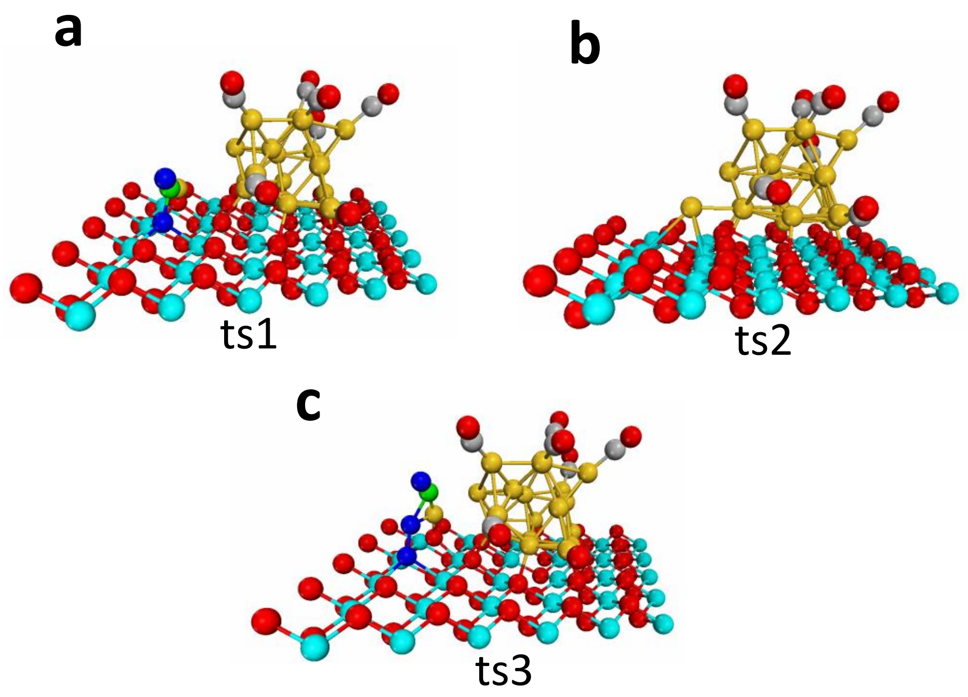

Supplementary Figure 4 | Transition state configurations for CO oxidation at single Au site. (ts1) CO oxidation with the lattice oxygen ion; **(**ts2) CO-Au diffuses from the oxygen defect to oxygen site; (ts3) CO oxidation with adsorbed O_2 species

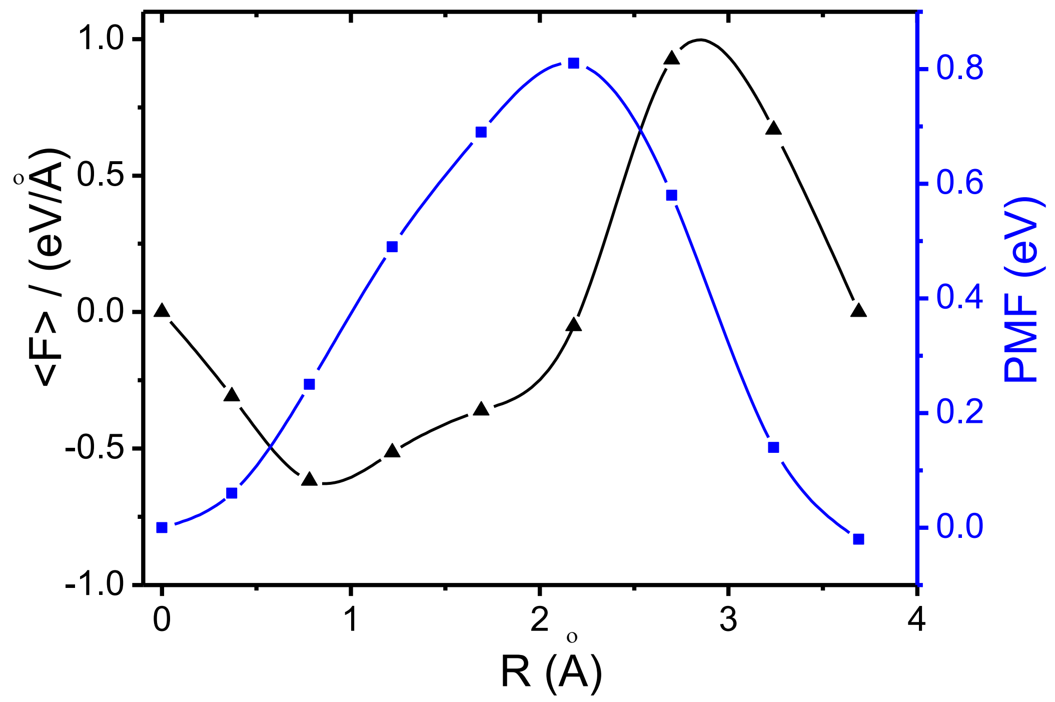

Supplementary Figure 5 | Free energy surface for the migration of the single Au at 300K. (<F> represents the expectation value of constrained force <F>; PMF represents the estimated free energy surface at 300 K; R is the distance between the single Au atom and the oxygen defect. See supplementary methods for computational details.)

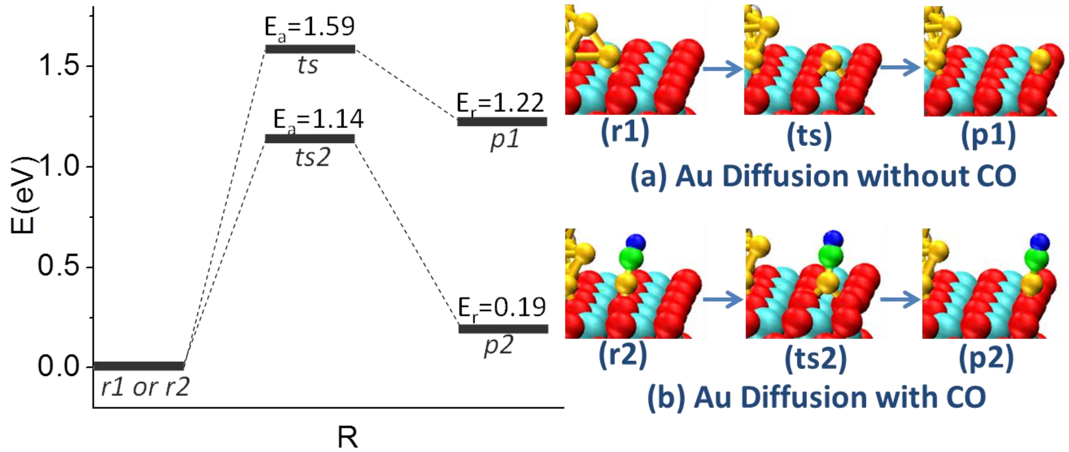

Supplementary Figure 6 | Diffusion of the single Au atom. (a) The diffusion of Au atom from the interface to the next site away from the interface. (b) The diffusion of Au-CO unit from the interface to the next site away from the interface.

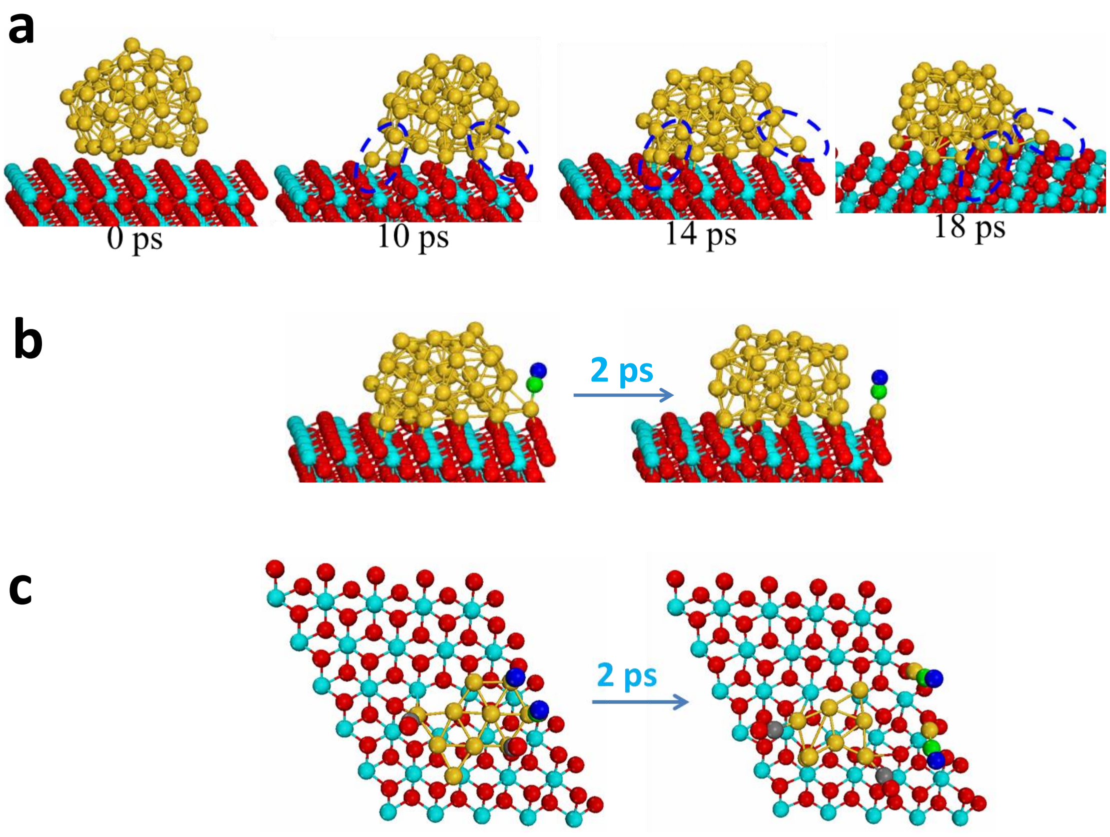

Supplementary Figure 7 | Snapshots of MD simulations for ceria supported Au_{50} or Au_{10} cluster. (a) The evolution of the low coordinated sites for Au_{50}/CeO_{20}. The low coordinated sites are circled. As is shown here, the formation of the low-coordinate site at the interface area are very frequent which indicating that under realistic conditions there should always exist low-coordinated sites for CO adsorption at the interface. (b) Initial and Final configurations for Au_{50}/CeO_2 systems with 1 CO (The CO is initially put to bind with the 4-coordinated gold atom). (c) Initial and Final configurations for Au_{10}/CeO_2 (with 4 CO); (only two surface O-Ce-O layers are shown)

Supplementary Methods

Test calculations. In our previous studies[1,2], we have tested our basis set and potential for Ce and chosen to deal with the excess electrons in CeO_2 using the DFT+U (U=7 eV) approach to best reproduce band gap, the location of gap state, and the work function, which ensures that the redox chemistry is reproduced correctly. We also tested six isomers (Td, C1, C2v, C2h, Oh, D2h) of Au_{20} and confirmed that exchange-correlation functional and basis set for Au give a reasonable description for the current system. Here we further consider a single Au atom adsorbed at oxygen site on the $CeO_2(111)$, comparable with previous studies [3,4]. We found one Ce ion adjacent to the single Au was reduced to Ce^{3+} with a net spin of 0.8. And the Au atom itself acquired a positive charge of $+0.29|e|$ with no net spin, consistent with Liu's study [3]. These results indicates that the single Au at the oxygen site become to Au^{+} by transferring one electron to the lattice Ce ion.

CO coverage. Considering the binding energies, CeO_2-supported Au_{20} cluster is estimated to be able to bind up to 10 CO molecules and the average binding energy is 0.87 eV per CO. This result is consistent with our recent study[1] on TiO_2-supported Au_{20} cluster where we also found the system can at most bind 10 CO. Therefore in our MD simulations we consider relative high coverage of CO on the Au_{20} cluster to explore the behavior of CO-induced reconstruction

Free energy surface. To obtain the free energy surface for reintegrating of the single Au into the Au cluster from the oxygen defect, we carried out potential of mean force (PMF) simulations at 300 K by moving the single Au atom toward the Au cluster. In this approach we constrained the Au atom along the reaction coordinate defined by the distance x between the single Au atom and the oxygen defect which was the collective variable most consistent with our results based on, the CINEB calculation of the same process. The initial and final configurations are the geometry-optimized configurations, which are shown in Figure 3 in the main text (Configuration v and vi). The average force on the constrained coordinate, $\langle F \rangle$ was obtained by ensemble-averages from 9 independent AIMD simulations of duration of 2ps of equilibrated trajectory after 2ps of pre-equilibrated simulation time. Increments in dx were

chosen to be 0.3-0.5Å apart depending on the steepness of the PMF to better estimate the free-energetics via thermodynamic integration. The force on each point in the PMF was ensured to converge to within ±0.001 a.u. The values of <F> were converted to a potential of mean force (PMF) based on the Blue-Moon ensemble method [5]. Integrating the average force along the reaction coordinate generated the free energy profile.

Supplementary References

1 Wang, Y.-G., Yoon, Y., Glezakou, V.-A., Li, J. & Rousseau, R. The role of reducible oxide–metal cluster charge transfer in catalytic processes: New insights on the catalytic mechanism of CO oxidation on Au/TiO_2 from ab initio molecular dynamics. *J. Am. Chem. Soc.* **135**, 10673-10683 (2013).

2 Wang, Y.-G., Mei, D., Li, J. & Rousseau, R. DFT+U Study on the Localized Electronic States and Their Potential Role During H_2O Dissociation and CO Oxidation Processes on CeO_2(111) Surface. *J. Phys. Chem. Lett* **4**, 2256-2363 (2013).

3 Liu, Z. P., Jenkins, S. J. & King, D. A. Origin and activity of oxidized gold in water-gas-shift catalysis. *Phys. Rev. Lett.* **94**, 196102 (2005).

4 Zhang, C., Michaelides, A., King, D. A. & Jenkins, S. J. Structure of gold atoms on stoichiometric and defective ceria surfaces. *J. Chem. Phys.* **129**, 194708 (2008).

5 Carter, E. A., Ciccotti, G.., Hynes, J. T., Kapral, R.. Constrained reaction coordinate dynamics for the simulation of rare events. *Chem. Phys. Lett.* **256**, 472-477, 1989.
