# Supplementary material for: Dynamic formation of single-atom catalytic active sites on ceria-supported gold nanoparticles
Source: Nat Commun. 2015 Mar 4;6:6511. doi: 10.1038/ncomms7511 (PMC4366521; doi:10.1038/ncomms7511)
Supplement: Supplementary Data 1 — Coordinates and energies of all stationary points on the potential energy surface [file ncomms7511-s2.doc]

The coordinates for all intermediates

1. Initial coordinates for Au_20_, Au_50_, and Au_10_ on CeO_2_ for the MD simulation

2. Intermediates for CO oxidation at single Au site

1. Initial coordinates for Au_20_, Au_50_, and Au_10_ on CeO_2_ for the MD simulation

(1) Au_20_/CeO_2_

O 0.0000000000 0.0000000000 6.2584769180

O -1.9162593770 3.3190586010 6.2584769180

O -3.8325187540 6.6381172030 6.2584769180

O -5.7487781310 9.9571758040 6.2584769180

O -7.6650375080 13.2762344060 6.2584769180

O -9.5812968850 16.5952930070 6.2584769180

O 3.8325187540 0.0000000000 6.2584769180

O 1.9162593770 3.3190586010 6.2584769180

O 0.0000000000 6.6381172030 6.2584769180

O -1.9162593770 9.9571758040 6.2584769180

O -3.8325187540 13.2762344060 6.2584769180

O -5.7487781310 16.5952930070 6.2584769180

O 7.6650375080 0.0000000000 6.2584769180

O 5.7487781310 3.3190586010 6.2584769180

O 3.8325187540 6.6381172030 6.2584769180

O 1.9162593770 9.9571758040 6.2584769180

O 0.0000000000 13.2762344060 6.2584769180

O -1.9162593770 16.5952930070 6.2584769180

O 11.4975562620 0.0000000000 6.2584769180

O 9.5812968850 3.3190586010 6.2584769180

O 7.6650375080 6.6381172030 6.2584769180

O 5.7487781310 9.9571758040 6.2584769180

O 3.8325187540 13.2762344060 6.2584769180

O 1.9162593770 16.5952930070 6.2584769180

O 15.3300750160 0.0000000000 6.2584769180

O 13.4138156390 3.3190586010 6.2584769180

O 11.4975562620 6.6381172030 6.2584769180

O 9.5812968850 9.9571758040 6.2584769180

O 7.6650375080 13.2762344060 6.2584769180

O 5.7487781310 16.5952930070 6.2584769180

O 0.0000000000 2.2127057340 9.3877153770

O -1.9162593770 5.5317643360 9.3877153770

O -3.8325187540 8.8508229370 9.3877153770

O -5.7487781310 12.1698815390 9.3877153770

O -7.6650375080 15.4889401400 9.3877153770

O -9.5812968850 18.8079987420 9.3877153770

O 3.8325187540 2.2127057340 9.3877153770

O 1.9162593770 5.5317643360 9.3877153770

O 0.0000000000 8.8508229370 9.3877153770

O -1.9162593770 12.1698815390 9.3877153770

O -3.8325187540 15.4889401400 9.3877153770

O -5.7487781310 18.8079987420 9.3877153770

O 7.6650375080 2.2127057340 9.3877153770

O 5.7487781310 5.5317643360 9.3877153770

O 3.8325187540 8.8508229370 9.3877153770

O 1.9162593770 12.1698815390 9.3877153770

O 0.0000000000 15.4889401400 9.3877153770

O -1.9162593770 18.8079987420 9.3877153770

O 11.4975562620 2.2127057340 9.3877153770

O 9.5812968850 5.5317643360 9.3877153770

O 7.6650375080 8.8508229370 9.3877153770

O 5.7487781310 12.1698815390 9.3877153770

O 3.8325187540 15.4889401400 9.3877153770

O 1.9162593770 18.8079987420 9.3877153770

O 15.3300750160 2.2127057340 9.3877153770

O 13.4138156390 5.5317643360 9.3877153770

O 11.4975562620 8.8508229370 9.3877153770

O 9.5812968850 12.1698815390 9.3877153770

O 7.6650375080 15.4889401400 9.3877153770

O 5.7487781310 18.8079987420 9.3877153770

O 1.9162593770 1.1063528670 3.1292384590

O 0.0000000000 4.4254114690 3.1292384590

O -1.9162593770 7.7444700700 3.1292384590

O -3.8325187540 11.0635286720 3.1292384590

O -5.7487781310 14.3825872730 3.1292384590

O -7.6650375080 17.7016458750 3.1292384590

O 5.7487781310 1.1063528670 3.1292384590

O 3.8325187540 4.4254114690 3.1292384590

O 1.9162593770 7.7444700700 3.1292384590

O 0.0000000000 11.0635286720 3.1292384590

O -1.9162593770 14.3825872730 3.1292384590

O -3.8325187540 17.7016458750 3.1292384590

O 9.5812968850 1.1063528670 3.1292384590

O 7.6650375080 4.4254114690 3.1292384590

O 5.7487781310 7.7444700700 3.1292384590

O 3.8325187540 11.0635286720 3.1292384590

O 1.9162593770 14.3825872730 3.1292384590

O 0.0000000000 17.7016458750 3.1292384590

O 13.4138156390 1.1063528670 3.1292384590

O 11.4975562620 4.4254114690 3.1292384590

O 9.5812968850 7.7444700700 3.1292384590

O 7.6650375080 11.0635286720 3.1292384590

O 5.7487781310 14.3825872730 3.1292384590

O 3.8325187540 17.7016458750 3.1292384590

O 17.2463343930 1.1063528670 3.1292384590

O 15.3300750160 4.4254114690 3.1292384590

O 13.4138156390 7.7444700700 3.1292384590

O 11.4975562620 11.0635286720 3.1292384590

O 9.5812968850 14.3825872730 3.1292384590

O 7.6650375080 17.7016458750 3.1292384590

O 1.9162593770 1.1063528670 12.5169538360

O 0.0000000000 4.4254114690 12.5169538360

O -1.9162593770 7.7444700700 12.5169538360

O -3.8325187540 11.0635286720 12.5169538360

O -5.7487781310 14.3825872730 12.5169538360

O -7.6650375080 17.7016458750 12.5169538360

O 5.7487781310 1.1063528670 12.5169538360

O 3.8325187540 4.4254114690 12.5169538360

O 1.9162593770 7.7444700700 12.5169538360

O 0.0000000000 11.0635286720 12.5169538360

O -1.9162593770 14.3825872730 12.5169538360

O -3.8325187540 17.7016458750 12.5169538360

O 9.5812968850 1.1063528670 12.5169538360

O 7.6650375080 4.4254114690 12.5169538360

O 5.7487781310 7.7444700700 12.5169538360

O 3.8325187540 11.0635286720 12.5169538360

O 1.9162593770 14.3825872730 12.5169538360

O 0.0000000000 17.7016458750 12.5169538360

O 13.4138156390 1.1063528670 12.5169538360

O 11.4975562620 4.4254114690 12.5169538360

O 9.5812968850 7.7444700700 12.5169538360

O 7.6650375080 11.0635286720 12.5169538360

O 5.7487781310 14.3825872730 12.5169538360

O 3.8325187540 17.7016458750 12.5169538360

O 17.2463343930 1.1063528670 12.5169538360

O 15.3300750160 4.4254114690 12.5169538360

O 13.4138156390 7.7444700700 12.5169538360

O 11.4975562620 11.0635286720 12.5169538360

O 9.5812968850 14.3825872730 12.5169538360

O 7.6650375080 17.7016458750 12.5169538360

O 0.0000000000 2.2127057340 4.6938576890

O -1.9162593770 5.5317643360 4.6938576890

O -3.8325187540 8.8508229370 4.6938576890

O -5.7487781310 12.1698815390 4.6938576890

O -7.6650375080 15.4889401400 4.6938576890

O -9.5812968850 18.8079987420 4.6938576890

O 3.8325187540 2.2127057340 4.6938576890

O 1.9162593770 5.5317643360 4.6938576890

O 0.0000000000 8.8508229370 4.6938576890

O -1.9162593770 12.1698815390 4.6938576890

O -3.8325187540 15.4889401400 4.6938576890

O -5.7487781310 18.8079987420 4.6938576890

O 7.6650375080 2.2127057340 4.6938576890

O 5.7487781310 5.5317643360 4.6938576890

O 3.8325187540 8.8508229370 4.6938576890

O 1.9162593770 12.1698815390 4.6938576890

O 0.0000000000 15.4889401400 4.6938576890

O -1.9162593770 18.8079987420 4.6938576890

O 11.4975562620 2.2127057340 4.6938576890

O 9.5812968850 5.5317643360 4.6938576890

O 7.6650375080 8.8508229370 4.6938576890

O 5.7487781310 12.1698815390 4.6938576890

O 3.8325187540 15.4889401400 4.6938576890

O 1.9162593770 18.8079987420 4.6938576890

O 15.3300750160 2.2127057340 4.6938576890

O 13.4138156390 5.5317643360 4.6938576890

O 11.4975562620 8.8508229370 4.6938576890

O 9.5812968850 12.1698815390 4.6938576890

O 7.6650375080 15.4889401400 4.6938576890

O 5.7487781310 18.8079987420 4.6938576890

O 0.0000000000 0.0000000000 10.9523346070

O -1.9162593770 3.3190586010 10.9523346070

O -3.8325187540 6.6381172030 10.9523346070

O -5.7487781310 9.9571758040 10.9523346070

O -7.6650375080 13.2762344060 10.9523346070

O -9.5812968850 16.5952930070 10.9523346070

O 3.8325187540 0.0000000000 10.9523346070

O 1.9162593770 3.3190586010 10.9523346070

O 0.0000000000 6.6381172030 10.9523346070

O -1.9162593770 9.9571758040 10.9523346070

O -3.8325187540 13.2762344060 10.9523346070

O -5.7487781310 16.5952930070 10.9523346070

O 7.6650375080 0.0000000000 10.9523346070

O 5.7487781310 3.3190586010 10.9523346070

O 3.8325187540 6.6381172030 10.9523346070

O 1.9162593770 9.9571758040 10.9523346070

O 0.0000000000 13.2762344060 10.9523346070

O -1.9162593770 16.5952930070 10.9523346070

O 11.4975562620 0.0000000000 10.9523346070

O 9.5812968850 3.3190586010 10.9523346070

O 7.6650375080 6.6381172030 10.9523346070

O 5.7487781310 9.9571758040 10.9523346070

O 3.8325187540 13.2762344060 10.9523346070

O 1.9162593770 16.5952930070 10.9523346070

O 15.3300750160 0.0000000000 10.9523346070

O 13.4138156390 3.3190586010 10.9523346070

O 11.4975562620 6.6381172030 10.9523346070

O 9.5812968850 9.9571758040 10.9523346070

O 7.6650375080 13.2762344060 10.9523346070

O 5.7487781310 16.5952930070 10.9523346070

O 0.0000000000 2.2127057340 14.0815730660

O -1.9162593770 5.5317643360 14.0815730660

O -3.8325187540 8.8508229370 14.0815730660

O -5.7487781310 12.1698815390 14.0815730660

O -7.6650375080 15.4889401400 14.0815730660

O -9.5812968850 18.8079987420 14.0815730660

O 3.8325187540 2.2127057340 14.0815730660

O 1.9162593770 5.5317643360 14.0815730660

O 0.0000000000 8.8508229370 14.0815730660

O -1.9162593770 12.1698815390 14.0815730660

O -3.8325187540 15.4889401400 14.0815730660

O -5.7487781310 18.8079987420 14.0815730660

O 7.6650375080 2.2127057340 14.0815730660

O 5.7487781310 5.5317643360 14.0815730660

O 1.9162593770 12.1698815390 14.0815730660

O 0.0000000000 15.4889401400 14.0815730660

O -1.9162593770 18.8079987420 14.0815730660

O 11.4975562620 2.2127057340 14.0815730660

O 9.5812968850 5.5317643360 14.0815730660

O 7.6650375080 8.8508229370 14.0815730660

O 5.7487781310 12.1698815390 14.0815730660

O 3.8325187540 15.4889401400 14.0815730660

O 1.9162593770 18.8079987420 14.0815730660

O 15.3300750160 2.2127057340 14.0815730660

O 13.4138156390 5.5317643360 14.0815730660

O 11.4975562620 8.8508229370 14.0815730660

O 9.5812968850 12.1698815390 14.0815730660

O 7.6650375080 15.4889401400 14.0815730660

O 5.7487781310 18.8079987420 14.0815730660

O 1.9162593770 1.1063528670 7.8230961480

O 0.0000000000 4.4254114690 7.8230961480

O -1.9162593770 7.7444700700 7.8230961480

O -3.8325187540 11.0635286720 7.8230961480

O -5.7487781310 14.3825872730 7.8230961480

O -7.6650375080 17.7016458750 7.8230961480

O 5.7487781310 1.1063528670 7.8230961480

O 3.8325187540 4.4254114690 7.8230961480

O 1.9162593770 7.7444700700 7.8230961480

O 0.0000000000 11.0635286720 7.8230961480

O -1.9162593770 14.3825872730 7.8230961480

O -3.8325187540 17.7016458750 7.8230961480

O 9.5812968850 1.1063528670 7.8230961480

O 7.6650375080 4.4254114690 7.8230961480

O 5.7487781310 7.7444700700 7.8230961480

O 3.8325187540 11.0635286720 7.8230961480

O 1.9162593770 14.3825872730 7.8230961480

O 0.0000000000 17.7016458750 7.8230961480

O 13.4138156390 1.1063528670 7.8230961480

O 11.4975562620 4.4254114690 7.8230961480

O 9.5812968850 7.7444700700 7.8230961480

O 7.6650375080 11.0635286720 7.8230961480

O 5.7487781310 14.3825872730 7.8230961480

O 3.8325187540 17.7016458750 7.8230961480

O 17.2463343930 1.1063528670 7.8230961480

O 15.3300750160 4.4254114690 7.8230961480

O 13.4138156390 7.7444700700 7.8230961480

O 11.4975562620 11.0635286720 7.8230961480

O 9.5812968850 14.3825872730 7.8230961480

O 7.6650375080 17.7016458750 7.8230961480

Ce 0.0000000000 0.0000000000 3.9115480740

Ce -1.9162593770 3.3190586010 3.9115480740

Ce -3.8325187540 6.6381172030 3.9115480740

Ce -5.7487781310 9.9571758040 3.9115480740

Ce -7.6650375080 13.2762344060 3.9115480740

Ce -9.5812968850 16.5952930070 3.9115480740

Ce 3.8325187540 0.0000000000 3.9115480740

Ce 1.9162593770 3.3190586010 3.9115480740

Ce 0.0000000000 6.6381172030 3.9115480740

Ce -1.9162593770 9.9571758040 3.9115480740

Ce -3.8325187540 13.2762344060 3.9115480740

Ce -5.7487781310 16.5952930070 3.9115480740

Ce 7.6650375080 0.0000000000 3.9115480740

Ce 5.7487781310 3.3190586010 3.9115480740

Ce 3.8325187540 6.6381172030 3.9115480740

Ce 1.9162593770 9.9571758040 3.9115480740

Ce 0.0000000000 13.2762344060 3.9115480740

Ce -1.9162593770 16.5952930070 3.9115480740

Ce 11.4975562620 0.0000000000 3.9115480740

Ce 9.5812968850 3.3190586010 3.9115480740

Ce 7.6650375080 6.6381172030 3.9115480740

Ce 5.7487781310 9.9571758040 3.9115480740

Ce 3.8325187540 13.2762344060 3.9115480740

Ce 1.9162593770 16.5952930070 3.9115480740

Ce 15.3300750160 0.0000000000 3.9115480740

Ce 13.4138156390 3.3190586010 3.9115480740

Ce 11.4975562620 6.6381172030 3.9115480740

Ce 9.5812968850 9.9571758040 3.9115480740

Ce 7.6650375080 13.2762344060 3.9115480740

Ce 5.7487781310 16.5952930070 3.9115480740

Ce 0.0000000000 0.0000000000 13.2992634510

Ce -1.9162593770 3.3190586010 13.2992634510

Ce -3.8325187540 6.6381172030 13.2992634510

Ce -5.7487781310 9.9571758040 13.2992634510

Ce -7.6650375080 13.2762344060 13.2992634510

Ce -9.5812968850 16.5952930070 13.2992634510

Ce 3.8325187540 0.0000000000 13.2992634510

Ce 1.9162593770 3.3190586010 13.2992634510

Ce 0.0000000000 6.6381172030 13.2992634510

Ce -1.9162593770 9.9571758040 13.2992634510

Ce -3.8325187540 13.2762344060 13.2992634510

Ce -5.7487781310 16.5952930070 13.2992634510

Ce 7.6650375080 0.0000000000 13.2992634510

Ce 5.7487781310 3.3190586010 13.2992634510

Ce 3.8325187540 6.6381172030 13.2992634510

Ce 1.9162593770 9.9571758040 13.2992634510

Ce 0.0000000000 13.2762344060 13.2992634510

Ce -1.9162593770 16.5952930070 13.2992634510

Ce 11.4975562620 0.0000000000 13.2992634510

Ce 9.5812968850 3.3190586010 13.2992634510

Ce 7.6650375080 6.6381172030 13.2992634510

Ce 5.7487781310 9.9571758040 13.2992634510

Ce 3.8325187540 13.2762344060 13.2992634510

Ce 1.9162593770 16.5952930070 13.2992634510

Ce 15.3300750160 0.0000000000 13.2992634510

Ce 13.4138156390 3.3190586010 13.2992634510

Ce 11.4975562620 6.6381172030 13.2992634510

Ce 9.5812968850 9.9571758040 13.2992634510

Ce 7.6650375080 13.2762344060 13.2992634510

Ce 5.7487781310 16.5952930070 13.2992634510

Ce 1.9162593770 1.1063528670 10.1700249920

Ce 0.0000000000 4.4254114690 10.1700249920

Ce -1.9162593770 7.7444700700 10.1700249920

Ce -3.8325187540 11.0635286720 10.1700249920

Ce -5.7487781310 14.3825872730 10.1700249920

Ce -7.6650375080 17.7016458750 10.1700249920

Ce 5.7487781310 1.1063528670 10.1700249920

Ce 3.8325187540 4.4254114690 10.1700249920

Ce 1.9162593770 7.7444700700 10.1700249920

Ce 0.0000000000 11.0635286720 10.1700249920

Ce -1.9162593770 14.3825872730 10.1700249920

Ce -3.8325187540 17.7016458750 10.1700249920

Ce 9.5812968850 1.1063528670 10.1700249920

Ce 7.6650375080 4.4254114690 10.1700249920

Ce 5.7487781310 7.7444700700 10.1700249920

Ce 3.8325187540 11.0635286720 10.1700249920

Ce 1.9162593770 14.3825872730 10.1700249920

Ce 0.0000000000 17.7016458750 10.1700249920

Ce 13.4138156390 1.1063528670 10.1700249920

Ce 11.4975562620 4.4254114690 10.1700249920

Ce 9.5812968850 7.7444700700 10.1700249920

Ce 7.6650375080 11.0635286720 10.1700249920

Ce 5.7487781310 14.3825872730 10.1700249920

Ce 3.8325187540 17.7016458750 10.1700249920

Ce 17.2463343930 1.1063528670 10.1700249920

Ce 15.3300750160 4.4254114690 10.1700249920

Ce 13.4138156390 7.7444700700 10.1700249920

Ce 11.4975562620 11.0635286720 10.1700249920

Ce 9.5812968850 14.3825872730 10.1700249920

Ce 7.6650375080 17.7016458750 10.1700249920

Ce 0.0000000000 2.2127057340 7.0407865330

Ce -1.9162593770 5.5317643360 7.0407865330

Ce -3.8325187540 8.8508229370 7.0407865330

Ce -5.7487781310 12.1698815390 7.0407865330

Ce -7.6650375080 15.4889401400 7.0407865330

Ce -9.5812968850 18.8079987420 7.0407865330

Ce 3.8325187540 2.2127057340 7.0407865330

Ce 1.9162593770 5.5317643360 7.0407865330

Ce 0.0000000000 8.8508229370 7.0407865330

Ce -1.9162593770 12.1698815390 7.0407865330

Ce -3.8325187540 15.4889401400 7.0407865330

Ce -5.7487781310 18.8079987420 7.0407865330

Ce 7.6650375080 2.2127057340 7.0407865330

Ce 5.7487781310 5.5317643360 7.0407865330

Ce 3.8325187540 8.8508229370 7.0407865330

Ce 1.9162593770 12.1698815390 7.0407865330

Ce 0.0000000000 15.4889401400 7.0407865330

Ce -1.9162593770 18.8079987420 7.0407865330

Ce 11.4975562620 2.2127057340 7.0407865330

Ce 9.5812968850 5.5317643360 7.0407865330

Ce 7.6650375080 8.8508229370 7.0407865330

Ce 5.7487781310 12.1698815390 7.0407865330

Ce 3.8325187540 15.4889401400 7.0407865330

Ce 1.9162593770 18.8079987420 7.0407865330

Ce 15.3300750160 2.2127057340 7.0407865330

Ce 13.4138156390 5.5317643360 7.0407865330

Ce 11.4975562620 8.8508229370 7.0407865330

Ce 9.5812968850 12.1698815390 7.0407865330

Ce 7.6650375080 15.4889401400 7.0407865330

Ce 5.7487781310 18.8079987420 7.0407865330

Au 0.3803286360 5.8337715120 17.7239433660

Au 8.5662782950 6.2114211140 17.7189831330

Au 4.1462667080 13.1117907230 17.7478675850

Au 4.3694840580 8.3609820170 24.4211280620

Au 2.7484101510 9.2141530730 20.0512845130

Au 5.8998332900 9.3595400590 20.0493740460

Au 4.3640908450 8.3866178380 17.4710978160

Au 4.4500246390 6.5576539520 20.0401653140

Au 3.1274491240 5.8112709210 17.6160352680

Au 5.8327698930 5.9360788030 17.6143964100

Au 4.2141845770 11.6703394730 20.0880442740

Au 2.9082979110 7.4339656000 22.2846810160

Au 7.2124251760 8.6023888220 17.6231606910

Au 5.7516850070 10.8828457810 17.6327059400

Au 1.5899490640 6.5987655070 20.0713746350

Au 5.9072310000 7.5723179350 22.2828642860

Au 2.7527528840 10.7444927520 17.6345234160

Au 1.5081730830 8.3392296200 17.6266172710

Au 7.2942013020 6.8619251260 20.0679171280

Au 4.2879539030 10.1002764290 22.2934458600

(2) Au_10_

Au 1.762061101 12.684472389 16.338644346

Au 4.524198984 12.626140811 16.423862511

Au 5.869515790 10.284102202 16.491411092

Au 4.439746151 7.905003196 16.474418839

Au 1.665407825 7.925859561 16.389248989

Au 0.370607256 10.296234631 16.322927980

Au 3.095177464 10.304958567 16.406241242

Au 3.010724631 5.583820952 16.456797570

Au 7.164316359 7.913727132 16.557732100

Au 5.772862514 5.525489374 16.542015735

(3) Au_50_

Au 3.744498617 8.368361633 10.796897107

Au 6.134288463 15.106793514 11.184209116

Au 9.573808536 9.064071713 15.622556316

Au 7.766381949 6.837009374 12.334714168

Au 8.282750685 13.375466918 10.793907259

Au 4.332191486 10.350350206 14.890522949

Au 9.961990885 14.545496966 15.278721122

Au 5.672670478 11.988940569 17.920458361

Au 7.555634589 15.850782010 15.687946070

Au 3.053614722 5.383794377 13.224529248

Au 3.077609437 14.682218185 14.894572417

Au 1.617428792 7.378364727 14.677926235

Au 3.684750634 10.014854874 17.751112012

Au 10.518823873 10.315128558 13.336860175

Au 10.323050555 11.390978472 10.712190476

Au 3.759023837 5.925775184 15.873204934

Au 7.497398846 7.991445907 17.246382647

Au 5.569172849 15.335453600 13.861438579

Au 5.450581314 10.050809066 12.277051853

Au 1.333207224 9.702615333 13.066458559

Au 1.183530299 7.177337342 11.976150275

Au 3.076669166 11.251812991 11.247069334

Au 2.115506528 7.706618108 17.363920073

Au 9.219128896 8.865558351 11.142529117

Au 4.499401263 7.863606902 13.808644509

Au 8.248159634 15.029257732 13.054953271

Au 6.367712459 5.443496758 16.891611844

Au 5.294633923 6.060527669 11.509041494

Au 7.808303659 11.391133065 12.901873433

Au 10.291133746 7.564767744 13.373325020

Au 1.217479424 9.430148403 10.285835989

Au 2.833229622 5.655210816 10.377925838

Au 10.213238565 13.066494055 12.960232122

Au 10.053920168 11.831285104 15.643243806

Au 6.120622741 9.540971657 19.120243377

Au 6.514515706 8.273413518 10.250378809

Au 2.288835701 12.149407745 13.885669554

Au 5.380983714 14.288320294 16.467581488

Au 8.127777080 10.679777235 17.366206478

Au 3.168139014 12.451642320 16.556622985

Au 5.566381492 12.394716380 10.717542058

Au 4.797335082 7.448377013 17.953045106

Au 1.649004636 10.004101928 15.773027574

Au 6.544205936 11.995292616 15.159590414

Au 3.861737396 13.790650198 12.310406818

Au 6.887283252 9.125086221 14.622763396

Au 8.228712855 6.527326819 15.029341117

Au 5.867944656 5.301678919 14.096556682

Au 7.606520001 10.778451015 9.966098559

Au 8.116820515 13.430286224 17.054610806

2. Intermediates for CO oxidation at single Au site

(1) Au_20_/CeO_2_ with 7 CO (one Isolated CO is removed, Config. i)

O 0.0073794768 -0.0016532123 6.2270866604

O -1.9135611393 3.3202767363 6.2258746969

O -3.8274698184 6.6382455813 6.2310636057

O -5.7431616986 9.9573019593 6.2327431527

O -7.6601229902 13.2746951777 6.2319634242

O -9.5796868202 16.5929942738 6.2287226289

O 3.8238318683 0.0057879057 6.2276829362

O 1.9175548550 3.3197129506 6.2313897470

O -0.0045497159 6.6420674905 6.2301815605

O -1.9258715867 9.9559320153 6.2294203709

O -3.8411784915 13.2752235054 6.2293112116

O -5.7581528057 16.5917236322 6.2252793337

O 7.6431069903 0.0048857326 6.2245908448

O 5.7806839218 3.3254945181 6.2804646563

O 3.8744148029 6.6395082919 6.2887185544

O 1.9522842490 9.9535864186 6.2359830647

O -0.0154809123 13.2803316605 6.2309435613

O -1.9350700746 16.5910844901 6.2243866840

O 11.5280361190 0.0039697468 6.2302873955

O 9.6126270432 3.3227256775 6.2309526596

O 7.6962817652 6.6371351822 6.2872539878

O 5.7775024078 9.9579842479 6.2927841279

O 3.8606806013 13.2776486299 6.2896259866

O 1.9471152931 16.5936500251 6.2836134666

O 15.3449823347 -0.0028515360 6.2292931439

O 13.4306255084 3.3188358839 6.2300488494

O 11.5177244857 6.6376124843 6.2827253585

O 9.6003852219 9.9572253989 6.2848511374

O 7.6818006686 13.2742994286 6.2851185906

O 5.7650483442 16.5927339341 6.2347341458

O 0.0463211643 2.2136190774 9.4811579154

O -1.8673689642 5.5263615946 9.4774238757

O -3.7865722947 8.8492463800 9.4776090984

O -5.6974082794 12.1722854705 9.4844597793

O -7.6154948384 15.4916657760 9.4878594444

O -9.5278049746 18.8063737335 9.4842818425

O 3.8697841158 2.2139226435 9.4864207965

O 1.9459737764 5.5316141767 9.4910604809

O 0.0273467020 8.8542098041 9.4846372347

O -1.8783302280 12.1710071018 9.4812094879

O -3.7960654910 15.4935993828 9.4804937439

O -5.7124422239 18.8067407002 9.4243632107

O 7.6895233448 2.2142160835 9.4852163323

O 5.7820414275 5.5298638353 9.4896982383

O 3.8513915634 8.8477227985 9.4944535679

O 1.9309735550 12.1714296553 9.4875646371

O 0.0213525573 15.4953776224 9.4815453220

O -1.8957283751 18.8110856725 9.4200262528

O 11.5050787981 2.2109881868 9.4792222095

O 9.6547983044 5.5386608533 9.4986209785

O 7.7482996938 8.8468912146 9.4998404394

O 5.7561256606 12.1706072615 9.4911550063

O 3.8399980415 15.4882669477 9.4870362883

O 1.9230599011 18.8047852555 9.4236198740

O 15.3931411680 2.2107115885 9.4879084426

O 13.4767394666 5.5322797499 9.4901652907

O 11.5638395277 8.8461093625 9.4918783446

O 9.6449934753 12.1715319219 9.4954190806

O 7.7273066060 15.4915028078 9.4945651991

O 5.8092341821 18.8120877183 9.4881215229

O 1.9162593770 1.1063528670 3.1292384590

O 0.0000000000 4.4254114690 3.1292384590

O -1.9162593770 7.7444700700 3.1292384590

O -3.8325187540 11.0635286720 3.1292384590

O -5.7487781310 14.3825872730 3.1292384590

O -7.6650375080 17.7016458750 3.1292384590

O 5.7487781310 1.1063528670 3.1292384590

O 3.8325187540 4.4254114690 3.1292384590

O 1.9162593770 7.7444700700 3.1292384590

O 0.0000000000 11.0635286720 3.1292384590

O -1.9162593770 14.3825872730 3.1292384590

O -3.8325187540 17.7016458750 3.1292384590

O 9.5812968850 1.1063528670 3.1292384590

O 7.6650375080 4.4254114690 3.1292384590

O 5.7487781310 7.7444700700 3.1292384590

O 3.8325187540 11.0635286720 3.1292384590

O 1.9162593770 14.3825872730 3.1292384590

O 0.0000000000 17.7016458750 3.1292384590

O 13.4138156390 1.1063528670 3.1292384590

O 11.4975562620 4.4254114690 3.1292384590

O 9.5812968850 7.7444700700 3.1292384590

O 7.6650375080 11.0635286720 3.1292384590

O 5.7487781310 14.3825872730 3.1292384590

O 3.8325187540 17.7016458750 3.1292384590

O 17.2463343930 1.1063528670 3.1292384590

O 15.3300750160 4.4254114690 3.1292384590

O 13.4138156390 7.7444700700 3.1292384590

O 11.4975562620 11.0635286720 3.1292384590

O 9.5812968850 14.3825872730 3.1292384590

O 7.6650375080 17.7016458750 3.1292384590

O 1.9500249546 1.0999021887 12.6094914999

O 0.0242228849 4.4276465503 12.6044689456

O -1.8915215681 7.7469414897 12.6674435210

O -3.8081993302 11.0748874892 12.6725848277

O -5.6826355133 14.4406321530 12.6169074549

O -7.5718890411 17.7054791587 12.6048120509

O 5.8325684537 1.1037783385 12.6636594412

O 3.8906793568 4.3445711476 12.6814263592

O 1.9170663262 7.7308377840 12.7034251371

O -0.0547312966 11.1375829482 12.6163161685

O -1.8748476608 14.4427278593 12.6125253937

O -3.7873486656 17.7591164499 12.6068634472

O 9.6484040558 1.1083039454 12.6120707772

O 7.7245872397 4.4233411006 12.6831936959

O 5.8206626378 7.7289115370 12.7715744512

O 3.8229962844 11.0673345512 12.6974615018

O 1.9494453399 14.4431266039 12.6844714472

O 0.0316825252 17.7574098627 12.6109108094

O 13.4649530410 1.1070378483 12.6140200645

O 11.5529649308 4.4219617075 12.6735584793

O 9.6399858633 7.7505534554 12.6830540218

O 7.8059060827 11.0471952469 12.6981856255

O 5.6704224549 14.4565282291 12.6951302932

O 3.8749797009 17.6895872561 12.6859435334

O 17.2802609225 1.1047000194 12.6019835586

O 15.3700199721 4.4232793649 12.6108602150

O 13.5225657318 7.7456725997 12.6184028168

O 11.6178781497 11.0627365492 12.6132819358

O 9.6952179050 14.3954537676 12.6855211973

O 7.6967230722 17.7125700991 12.6000883576

O 0.0000000000 2.2127057340 4.6938576890

O -1.9162593770 5.5317643360 4.6938576890

O -3.8325187540 8.8508229370 4.6938576890

O -5.7487781310 12.1698815390 4.6938576890

O -7.6650375080 15.4889401400 4.6938576890

O -9.5812968850 18.8079987420 4.6938576890

O 3.8325187540 2.2127057340 4.6938576890

O 1.9162593770 5.5317643360 4.6938576890

O -0.0000000000 8.8508229370 4.6938576890

O -1.9162593770 12.1698815390 4.6938576890

O -3.8325187540 15.4889401400 4.6938576890

O -5.7487781310 18.8079987420 4.6938576890

O 7.6650375080 2.2127057340 4.6938576890

O 5.7487781310 5.5317643360 4.6938576890

O 3.8325187540 8.8508229370 4.6938576890

O 1.9162593770 12.1698815390 4.6938576890

O -0.0000000000 15.4889401400 4.6938576890

O -1.9162593770 18.8079987420 4.6938576890

O 11.4975562620 2.2127057340 4.6938576890

O 9.5812968850 5.5317643360 4.6938576890

O 7.6650375080 8.8508229370 4.6938576890

O 5.7487781310 12.1698815390 4.6938576890

O 3.8325187540 15.4889401400 4.6938576890

O 1.9162593770 18.8079987420 4.6938576890

O 15.3300750160 2.2127057340 4.6938576890

O 13.4138156390 5.5317643360 4.6938576890

O 11.4975562620 8.8508229370 4.6938576890

O 9.5812968850 12.1698815390 4.6938576890

O 7.6650375080 15.4889401400 4.6938576890

O 5.7487781310 18.8079987420 4.6938576890

O 0.0707003830 0.0018233045 11.0942489958

O -1.8494110290 3.3153024917 11.0960343711

O -3.7523037301 6.6346577879 11.0967390446

O -5.6649449686 9.9550989454 11.0980321051

O -7.5876447455 13.2759740719 11.1035558043

O -9.5066120455 16.5965045742 11.1033830100

O 3.9025177103 0.0025144012 11.0987254764

O 1.9781599103 3.3188890790 11.0985439310

O 0.0000275797 6.6413131901 11.1055318099

O -1.9234264082 9.9543292985 11.1031986574

O -3.7690777230 13.2749307860 11.1029740570

O -5.6806775563 16.6037682409 11.0903188980

O 7.7146610791 0.0026481509 11.0915694640

O 5.8045312958 3.3172746085 11.1071861865

O 3.8837323502 6.6338690304 11.0855957910

O 1.8932409541 9.9586656520 11.0801710170

O 0.0481489900 13.2898974183 11.1038247455

O -1.8708333043 16.6084316421 11.0913007275

O 11.5338382260 0.0021736257 11.0887390465

O 9.6180291128 3.3224970082 11.1033950796

O 7.7066094330 6.6375858136 11.1681575027

O 5.7933182053 9.9551548904 11.0343728457

O 3.8580528716 13.2739575781 11.1035248563

O 1.9481111835 16.6066228037 11.1023242390

O 15.3508231262 -0.0091307791 11.0952557024

O 13.4372369844 3.3164413102 11.0960066838

O 11.5820583118 6.6367390729 11.1616225490

O 9.6782837317 9.9579859723 11.1093613191

O 7.6833499988 13.2811219900 11.0989730133

O 5.7721071403 16.6002008037 11.0963279128

O 0.0451394221 2.2044880247 14.2118235637

O -1.8690900984 5.5248035160 14.2869438024

O -3.7835961784 8.8536290612 14.2901876847

O -5.6540429679 12.2382501772 14.2857940004

O -7.5702740043 15.5566436033 14.2890271086

O -9.5266691782 18.8098383360 14.2072172981

O 3.9064296421 2.1394494082 14.2861328119

O 1.8140748449 5.3234123616 14.3925588178

O -0.1499296790 8.8451031468 14.3798367336

O -1.9210000244 12.2444457393 14.2880775416

O -3.7572652966 15.5549148072 14.2818630792

O -5.7091020580 18.8118730677 14.2858083878

O 7.7646259501 2.2068873246 14.2864516316

O 5.9431707521 5.3822299086 14.5522375294

O 1.8445431142 12.3225492540 14.4617896916

O -0.0174397944 15.5612080931 14.2837236698

O -1.8928099223 18.8156768346 14.2850692908

O 11.5804402006 2.2109823017 14.2827404402

O 9.6712785965 5.5287475150 14.2890531987

O 7.9263161700 8.7171495777 14.5514434240

O 5.6632752900 12.3166939644 14.4596730451

O 3.7913350730 15.5534972051 14.5576837595

O 1.9222697685 18.8136774345 14.2838249855

O 15.3959985661 2.2118004518 14.2750270083

O 13.4837914266 5.5325864543 14.2831093273

O 11.6479504558 8.8498723437 14.2841704222

O 9.8643368127 12.0953511290 14.3754842114

O 7.7021428787 15.7040064144 14.3006146357

O 5.7840908012 18.8723720019 14.2781477642

O 1.9354002582 1.1069109587 7.8807951897

O 0.0192540118 4.4258595666 7.8286778854

O -1.8976983205 7.7442112519 7.8860899338

O -3.8148136037 11.0637760640 7.8880001538

O -5.7318191815 14.3818597965 7.8824628521

O -7.6513809114 17.6992188538 7.8275333237

O 5.7527336176 1.1087995309 7.8874839623

O 3.8369096960 4.4238292664 7.8928339061

O 1.9191970480 7.7441790910 7.8910327646

O 0.0046263106 11.0623246654 7.8257545073

O -1.9131506606 14.3827096498 7.8798188579

O -3.8292939116 17.6992564098 7.8227936323

O 9.5773770773 1.1087434493 7.8254936175

O 7.7276888810 4.4250603955 7.8993314874

O 5.8059635660 7.7464256628 7.9071750680

O 3.8223401130 11.0617574235 7.8959686781

O 1.9055924348 14.3804998718 7.8904391113

O -0.0109430573 17.6988652860 7.8218560026

O 13.4574808420 1.1126644207 7.8862458946

O 11.5471832564 4.4222658046 7.8942128008

O 9.6315537277 7.7436907020 7.8983043861

O 7.7103363510 11.0660516815 7.9032824955

O 5.7933245174 14.3877547781 7.9000304509

O 3.8825817522 17.7030353404 7.8952501124

O 17.2778178169 1.1055529052 7.8317693555

O 15.3622412899 4.4235026456 7.8827618262

O 13.4495217498 7.7439988084 7.8882291107

O 11.5336159525 11.0644852987 7.8905602474

O 9.6159686110 14.3833051025 7.8945727351

O 7.6973928630 17.7018346627 7.8838085418

Ce 0.0000000000 0.0000000000 3.9115480740

Ce -1.9162593770 3.3190586010 3.9115480740

Ce -3.8325187540 6.6381172030 3.9115480740

Ce -5.7487781310 9.9571758040 3.9115480740

Ce -7.6650375080 13.2762344060 3.9115480740

Ce -9.5812968850 16.5952930070 3.9115480740

Ce 3.8325187540 0.0000000000 3.9115480740

Ce 1.9162593770 3.3190586010 3.9115480740

Ce 0.0000000000 6.6381172030 3.9115480740

Ce -1.9162593770 9.9571758040 3.9115480740

Ce -3.8325187540 13.2762344060 3.9115480740

Ce -5.7487781310 16.5952930070 3.9115480740

Ce 7.6650375080 -0.0000000000 3.9115480740

Ce 5.7487781310 3.3190586010 3.9115480740

Ce 3.8325187540 6.6381172030 3.9115480740

Ce 1.9162593770 9.9571758040 3.9115480740

Ce 0.0000000000 13.2762344060 3.9115480740

Ce -1.9162593770 16.5952930070 3.9115480740

Ce 11.4975562620 -0.0000000000 3.9115480740

Ce 9.5812968850 3.3190586010 3.9115480740

Ce 7.6650375080 6.6381172030 3.9115480740

Ce 5.7487781310 9.9571758040 3.9115480740

Ce 3.8325187540 13.2762344060 3.9115480740

Ce 1.9162593770 16.5952930070 3.9115480740

Ce 15.3300750160 0.0000000000 3.9115480740

Ce 13.4138156390 3.3190586010 3.9115480740

Ce 11.4975562620 6.6381172030 3.9115480740

Ce 9.5812968850 9.9571758040 3.9115480740

Ce 7.6650375080 13.2762344060 3.9115480740

Ce 5.7487781310 16.5952930070 3.9115480740

Ce 0.0454818790 -0.0054329441 13.4175818041

Ce -1.8703192758 3.3060759538 13.4184464450

Ce -3.7861133931 6.6394015228 13.4339961130

Ce -5.6744148864 9.9648321777 13.4552062017

Ce -7.5846863920 13.2952371555 13.4779580369

Ce -9.5025046588 16.6097151801 13.4435893804

Ce 3.8973203375 -0.0181848117 13.4491335492

Ce 1.9477866620 3.2420178062 13.4378295797

Ce -0.0049075748 6.6194431560 13.4534611836

Ce -1.9538016808 9.9826189199 13.4579585120

Ce -3.7998300369 13.3194045865 13.4602890065

Ce -5.6977562927 16.6215309871 13.4559021634

Ce 7.7313271624 -0.0070061780 13.4539096767

Ce 5.8287253504 3.2334889124 13.4844760579

Ce 3.8781882297 6.4868334937 13.4900572056

Ce 1.7559571601 10.0359897220 13.4935917935

Ce -0.0332525976 13.3554140159 13.4691763378

Ce -1.8999624300 16.6412995711 13.4487295508

Ce 11.5430957851 0.0207509878 13.4411575587

Ce 9.6478441052 3.3161310946 13.4589865116

Ce 7.7899157675 6.6181053270 13.5182354613

Ce 5.9133337850 9.9712031253 13.4819535125

Ce 3.8187866243 13.2638588894 13.4516772149

Ce 1.8962254719 16.6521817069 13.4748915745

Ce 15.3761779233 0.0089957644 13.4474408453

Ce 13.4658729780 3.3161140786 13.4486708543

Ce 11.5830398202 6.6404171329 13.4718351367

Ce 9.7419916846 10.0114050985 13.4953055862

Ce 7.6914841852 13.3497777293 13.6573301680

Ce 5.8345960259 16.6492449918 13.4683348356

Ce 1.9666416394 1.1028886735 10.2678791783

Ce 0.0438168419 4.4285940801 10.2720043029

Ce -1.8782085759 7.7514911323 10.2756307510

Ce -3.7941682508 11.0701029301 10.2755609236

Ce -5.7064439658 14.3904092202 10.2823397694

Ce -7.6108947825 17.6945747846 10.2657943506

Ce 5.7912750235 1.1079011730 10.2671430859

Ce 3.8736028277 4.4293299306 10.2867239562

Ce 1.9489431986 7.7550860837 10.3340586342

Ce 0.0234862489 11.0687849245 10.2711401153

Ce -1.8825902205 14.3900448992 10.2728812945

Ce -3.7986522870 17.7045827000 10.2565962980

Ce 9.6108869835 1.1077433206 10.2641986182

Ce 7.7077022130 4.4342590177 10.3027381872

Ce 5.7898224971 7.7523190622 10.3345407565

Ce 3.8472189791 11.0486662742 10.3248866637

Ce 1.9408539505 14.3864639840 10.2860895327

Ce 0.0248312221 17.7071170495 10.2554086872

Ce 13.4464387932 1.1050921594 10.2657586904

Ce 11.5453137330 4.4305034645 10.2915641198

Ce 9.6376039029 7.7593116806 10.3146006161

Ce 7.7179163408 11.0818410107 10.3237855927

Ce 5.7941157988 14.3728894755 10.3200030738

Ce 3.8686506200 17.6918759656 10.2841747609

Ce 17.2960253929 1.1002773685 10.2639030440

Ce 15.3826306100 4.4241617069 10.2706391513

Ce 13.4777364524 7.7495423720 10.2809703619

Ce 11.5614906594 11.0681121636 10.2801017845

Ce 9.6214817312 14.3685889501 10.3293035465

Ce 7.7150981502 17.6935780253 10.2704915144

Ce 0.0189154638 2.2147531818 7.0458139823

Ce -1.8984442910 5.5341438463 7.0539238333

Ce -3.8164118086 8.8522549453 7.0600430861

Ce -5.7364437167 12.1710093749 7.0604763462

Ce -7.6559886984 15.4839907133 7.0598976393

Ce -9.5656584658 18.8045948219 7.0509996307

Ce 3.8463530174 2.2179262380 7.0660772499

Ce 1.9345547412 5.5420670240 7.0707989909

Ce 0.0194482835 8.8495708646 7.0612131150

Ce -1.9096211100 12.1710989703 7.0514525452

Ce -3.8308147284 15.4899124098 7.0502375164

Ce -5.7443861847 18.8077951488 7.0411598676

Ce 7.6808908734 2.2173679034 7.0599875208

Ce 5.7811384568 5.5419069716 7.0890497983

Ce 3.8557610133 8.8537913689 7.0919091888

Ce 1.9320380248 12.1682070091 7.0705552969

Ce 0.0044350777 15.4872524915 7.0581537283

Ce -1.9145340502 18.8069113421 7.0356534646

Ce 11.5163003167 2.2140000105 7.0523319492

Ce 9.6132467147 5.5367123741 7.0795145558

Ce 7.6974215284 8.8534806018 7.0989980844

Ce 5.7685067351 12.1646136422 7.1023339986

Ce 3.8565044800 15.4833948513 7.0813057991

Ce 1.9348194319 18.8035637618 7.0552870922

Ce 15.3535852946 2.2114665679 7.0502185943

Ce 13.4377795629 5.5333037137 7.0666567394

Ce 11.5191392471 8.8508821988 7.0769140230

Ce 9.6000521592 12.1717442150 7.0813049174

Ce 7.6896660369 15.4792149976 7.0754832940

Ce 5.7718298823 18.8018390969 7.0536676872

Au -0.7020948136 6.5441577681 17.1491177493

Au 6.6588535238 7.3909376854 18.8295162128

Au 3.7389722979 14.3585443877 16.4118071682

Au 0.6063188553 11.4517132164 21.0494955146

Au -0.2819801540 10.7257858161 18.3263682920

Au 4.9888863512 9.7813736705 18.5248097302

Au 3.7135611560 8.8136307947 16.0162716746

Au 2.4055406692 6.1360065080 21.0854859964

Au 2.1871335593 6.3400888612 16.4378450470

Au 5.3277789573 6.2251298633 16.4943305832

Au 3.7426314381 12.6302455480 18.4756558535

Au 4.0605072907 8.9604235235 21.0322529202

Au 6.7063805188 8.9672547062 16.4528277363

Au 5.0688587980 11.4059402693 16.3433115845

Au 1.2279750511 7.3470918851 18.9593025884

Au 2.2945185796 10.2264378671 19.2710465550

Au 1.9498530271 11.3762421871 16.6539153558

Au 0.8041048490 8.7901155504 16.5937634562

Au 3.9572595333 7.0679471429 18.6947289731

Au 1.0520556083 8.7174332614 21.3830412426

C -2.5444134559 5.7969621711 17.4650756308

O -3.6308462604 5.8244694244 17.8377631207

C 8.2466250523 7.2185080303 20.0056141094

O 9.0083796703 7.3008290367 20.8609122335

C -0.3697329319 8.1129050323 22.7326013738

O -1.4057075652 7.6746108974 22.9861643851

C 5.1722055003 8.5106081180 22.6620593059

O 6.0910276780 8.3366751902 23.3350086618

C -0.6483390396 12.5100136199 22.2030788496

O -1.5628854383 12.5433576944 22.9013428511

C 2.5997445322 4.7677341950 22.5710260492

O 2.2736630301 4.0641382367 23.4204990576

C -2.2908996516 10.5512580255 18.2614227210

O -3.2660746715 9.9605293461 18.1064714557

(2) CO adsorption forming an Au^+^-CO; (Cofig. ii)

O 0.0077007898 -0.0006302766 6.2290356992

O -1.9104190085 3.3230133194 6.2304048977

O -3.8260852346 6.6379711677 6.2340421478

O -5.7425587240 9.9570944471 6.2338586467

O -7.6600482040 13.2742112576 6.2326214515

O -9.5798017390 16.5930216122 6.2293183721

O 3.8244268387 0.0067194988 6.2284996594

O 1.9153287367 3.3226258081 6.2794112357

O -0.0028998394 6.6394822614 6.2811923164

O -1.9208645583 9.9576577559 6.2808334521

O -3.8394415106 13.2746050983 6.2318419313

O -5.7572911830 16.5918929563 6.2259482475

O 7.6496504075 0.0077127519 6.2293238743

O 5.7838034673 3.3240204471 6.2846564527

O 3.8738092489 6.6394953341 6.2900033511

O 1.9459338180 9.9549615864 6.2763879317

O -0.0126542357 13.2752819294 6.2752340961

O -1.9338658161 16.5916085398 6.2261417180

O 11.5243038390 0.0054900657 6.2358069121

O 9.6134884430 3.3187877179 6.2844596650

O 7.6967155851 6.6377983803 6.2874315024

O 5.7769757239 9.9578221413 6.2915438516

O 3.8597876233 13.2766115400 6.2914929786

O 1.9463744310 16.5937541536 6.2856366899

O 15.3448282528 -0.0024829570 6.2304116143

O 13.4307537190 3.3188794206 6.2326194300

O 11.5182723377 6.6378416743 6.2836971910

O 9.6006489447 9.9574805514 6.2865107206

O 7.6821025923 13.2739297875 6.2861552718

O 5.7651040916 16.5927267493 6.2359923249

O 0.0510725203 2.2192811427 9.4862985939

O -1.8612654373 5.5335625084 9.4945041718

O -3.7772755323 8.8517656153 9.4922831278

O -5.6959119034 12.1697651788 9.4892021242

O -7.6146361775 15.4911165395 9.4873497213

O -9.5301930554 18.8089652630 9.4891065300

O 3.8676345755 2.2171792782 9.4902128700

O 1.9506229243 5.5313759076 9.4968161045

O 0.0303017869 8.8470475468 9.4940958588

O -1.8789882679 12.1697644983 9.4924329421

O -3.7959384950 15.4928936863 9.4831983178

O -5.7119375242 18.8064731715 9.4249531388

O 7.6896956191 2.2169188956 9.4899217623

O 5.8340541414 5.5317470522 9.4942533193

O 3.8545833846 8.8482117970 9.4985853944

O 1.9327093405 12.1722053732 9.4934862901

O 0.0208169904 15.4896801719 9.4868909504

O -1.8953403815 18.8083356606 9.4760179719

O 11.5054208714 2.2152842717 9.4862507985

O 9.6576555772 5.5369280102 9.5027733693

O 7.7485281926 8.8510658922 9.5062370679

O 5.7535554426 12.1695643254 9.4981836751

O 3.8398257684 15.4794631999 9.4959934796

O 1.9229175472 18.8050449278 9.4250391362

O 15.3924231525 2.2136942031 9.4893730548

O 13.4773312272 5.5323937588 9.4932924010

O 11.5608505699 8.8481629012 9.4962755085

O 9.6438254451 12.1667868088 9.4993732819

O 7.7255210880 15.4912024688 9.4978809428

O 5.8101253278 18.8131101582 9.4902569413

O 1.9162593770 1.1063528670 3.1292384590

O 0.0000000000 4.4254114690 3.1292384590

O -1.9162593770 7.7444700700 3.1292384590

O -3.8325187540 11.0635286720 3.1292384590

O -5.7487781310 14.3825872730 3.1292384590

O -7.6650375080 17.7016458750 3.1292384590

O 5.7487781310 1.1063528670 3.1292384590

O 3.8325187540 4.4254114690 3.1292384590

O 1.9162593770 7.7444700700 3.1292384590

O -0.0000000000 11.0635286720 3.1292384590

O -1.9162593770 14.3825872730 3.1292384590

O -3.8325187540 17.7016458750 3.1292384590

O 9.5812968850 1.1063528670 3.1292384590

O 7.6650375080 4.4254114690 3.1292384590

O 5.7487781310 7.7444700700 3.1292384590

O 3.8325187540 11.0635286720 3.1292384590

O 1.9162593770 14.3825872730 3.1292384590

O 0.0000000000 17.7016458750 3.1292384590

O 13.4138156390 1.1063528670 3.1292384590

O 11.4975562620 4.4254114690 3.1292384590

O 9.5812968850 7.7444700700 3.1292384590

O 7.6650375080 11.0635286720 3.1292384590

O 5.7487781310 14.3825872730 3.1292384590

O 3.8325187540 17.7016458750 3.1292384590

O 17.2463343930 1.1063528670 3.1292384590

O 15.3300750160 4.4254114690 3.1292384590

O 13.4138156390 7.7444700700 3.1292384590

O 11.4975562620 11.0635286720 3.1292384590

O 9.5812968850 14.3825872730 3.1292384590

O 7.6650375080 17.7016458750 3.1292384590

O 2.0032084942 1.1033479968 12.6150862457

O 0.0193998115 4.4267442620 12.6788836389

O -1.8904268331 7.7380756038 12.6783111611

O -3.8056978712 11.0735778069 12.6708844620

O -5.6810281003 14.4420604276 12.6164261124

O -7.5689719732 17.7131317000 12.6069899390

O 5.8318608735 1.1079774473 12.6772046775

O 3.9538085082 4.3621914565 12.6846813854

O 1.9990698853 7.7452791009 12.7796102636

O -0.0331619127 11.1313044569 12.6906167346

O -1.8776327812 14.4414527503 12.6183761734

O -3.7874751118 17.7587240140 12.6076237787

O 9.6479052272 1.1060001125 12.6210368756

O 7.7233750681 4.4292575400 12.6874004962

O 5.8190206144 7.7384366747 12.7847897770

O 3.8268023410 11.0565499047 12.7772734491

O 1.9552516539 14.4507332871 12.6914329627

O 0.0282980039 17.7616194072 12.6070774178

O 13.4652382995 1.1084839618 12.6153566325

O 11.5558678274 4.4240283998 12.6788779391

O 9.6397519316 7.7479731617 12.6867454124

O 7.8034832251 11.0537958206 12.7623164043

O 5.6381374925 14.3888069790 12.7015305230

O 3.8049909124 17.6982507237 12.6877460052

O 17.2813610185 1.1158626599 12.6045321083

O 15.3794210767 4.4255323760 12.6654951714

O 13.5241547683 7.7451254574 12.6214005671

O 11.6190689300 11.0669939451 12.6735354070

O 9.7095119032 14.4028403456 12.6682814590

O 7.7530873608 17.7755157043 12.6019166594

O 0.0000000000 2.2127057340 4.6938576890

O -1.9162593770 5.5317643360 4.6938576890

O -3.8325187540 8.8508229370 4.6938576890

O -5.7487781310 12.1698815390 4.6938576890

O -7.6650375080 15.4889401400 4.6938576890

O -9.5812968850 18.8079987420 4.6938576890

O 3.8325187540 2.2127057340 4.6938576890

O 1.9162593770 5.5317643360 4.6938576890

O 0.0000000000 8.8508229370 4.6938576890

O -1.9162593770 12.1698815390 4.6938576890

O -3.8325187540 15.4889401400 4.6938576890

O -5.7487781310 18.8079987420 4.6938576890

O 7.6650375080 2.2127057340 4.6938576890

O 5.7487781310 5.5317643360 4.6938576890

O 3.8325187540 8.8508229370 4.6938576890

O 1.9162593770 12.1698815390 4.6938576890

O -0.0000000000 15.4889401400 4.6938576890

O -1.9162593770 18.8079987420 4.6938576890

O 11.4975562620 2.2127057340 4.6938576890

O 9.5812968850 5.5317643360 4.6938576890

O 7.6650375080 8.8508229370 4.6938576890

O 5.7487781310 12.1698815390 4.6938576890

O 3.8325187540 15.4889401400 4.6938576890

O 1.9162593770 18.8079987420 4.6938576890

O 15.3300750160 2.2127057340 4.6938576890

O 13.4138156390 5.5317643360 4.6938576890

O 11.4975562620 8.8508229370 4.6938576890

O 9.5812968850 12.1698815390 4.6938576890

O 7.6650375080 15.4889401400 4.6938576890

O 5.7487781310 18.8079987420 4.6938576890

O 0.0484246438 0.0569330011 11.0962464614

O -1.8459822014 3.3228992656 11.1044194269

O -3.7518641703 6.6322347416 11.1014836994

O -5.6683464229 9.9566982265 11.1014352493

O -7.5857441699 13.2715379948 11.1041879554

O -9.5045791250 16.5970510277 11.1003094784

O 3.9042842955 0.0023298562 11.0974730739

O 1.9801945689 3.3283233947 11.1610596620

O 0.0670468569 6.6423585981 11.1772635294

O -1.8654158395 9.9540573447 11.1596204991

O -3.7692767794 13.2746767428 11.1039152597

O -5.6815455787 16.6030191535 11.0912822529

O 7.7108110315 0.0046040948 11.0950020285

O 5.8058411610 3.3150219010 11.1048255158

O 3.8872111476 6.6336417618 11.0893214393

O 1.8989084931 9.9571546783 11.0948393303

O 0.0490539842 13.2844008852 11.1641855052

O -1.8702569597 16.6034842537 11.0942089904

O 11.5363556047 0.0035753551 11.0911270364

O 9.6211064432 3.3238324782 11.1575899116

O 7.7080387824 6.6418924879 11.1748602456

O 5.7904874690 9.9607093968 11.0931512337

O 3.8231292173 13.2172931147 11.1643571552

O 1.9430986997 16.6032263931 11.0941299037

O 15.3531828624 -0.0075569276 11.0952802076

O 13.4426192622 3.3189955079 11.1004674298

O 11.5821531827 6.6381448493 11.1637062183

O 9.6777514422 9.9618202990 11.1720850290

O 7.6860684479 13.2784942625 11.1010931670

O 5.7756988257 16.5999163356 11.0917294081

O 0.0532638179 2.2067796416 14.2804264096

O -1.8663158885 5.5305020719 14.3639950174

O -3.7828900813 8.8542632560 14.2940203123

O -5.6499626139 12.2406397388 14.2888018280

O -7.5638622050 15.5572442678 14.2913174954

O -9.4844831721 18.8728291282 14.2836181833

O 3.8763531605 2.2002309847 14.2919311619

O 1.8635965262 5.3803823209 14.3818326141

O -0.1382420623 8.8464244287 14.3772392893

O -1.9240782478 12.2444065644 14.2921690500

O -3.7572079955 15.5559607766 14.2837101487

O -5.7059679280 18.8111222737 14.2883011399

O 7.7668320438 2.2075992507 14.2895744501

O 5.9419630048 5.3880060605 14.5510199426

O 1.8533339426 12.3208623391 14.4684463150

O -0.0208737884 15.5630260638 14.2875329327

O -1.8928195449 18.8143498608 14.2855804224

O 11.5811306735 2.2109051011 14.2861292782

O 9.6698272062 5.5307219730 14.2910713783

O 7.9618761991 8.7730079450 14.5560169845

O 5.6631335349 12.3204036085 14.5523740683

O 3.7402498234 15.4873935691 14.6511671266

O 1.9158009347 18.8165852527 14.2901198366

O 15.3957559048 2.2108167287 14.2833421157

O 13.4823692569 5.5344816318 14.2869618531

O 11.6491527669 8.8471367543 14.2885842499

O 9.8358960529 12.1575079623 14.3789335550

O 7.8743316795 15.6941902537 14.3798289602

O 5.8186286471 18.9562049268 14.2924994670

O 1.9353545737 1.1046476483 7.8876313742

O 0.0171650121 4.4256678961 7.8947895582

O -1.8995690664 7.7427460349 7.8950034453

O -3.8167422645 11.0641029686 7.8935601551

O -5.7314640612 14.3814004141 7.8837455642

O -7.6515815716 17.6998537330 7.8281525240

O 5.7521526401 1.1081846505 7.8912520335

O 3.8433242195 4.4266061224 7.8991791041

O 1.9222269100 7.7438423351 7.8996168293

O 0.0041847912 11.0626601446 7.8954006109

O -1.9151218069 14.3836228402 7.8879731674

O -3.8261096123 17.7008232344 7.8255547636

O 9.5782887997 1.1008948869 7.8812737149

O 7.7282625438 4.4252829529 7.8996372634

O 5.8058181563 7.7477086210 7.9111518346

O 3.8257448484 11.0621249396 7.9028355545

O 1.9071312789 14.3815241735 7.8943755514

O -0.0138971623 17.7001139690 7.8258282812

O 13.4583632727 1.1124222396 7.8888280971

O 11.5496822767 4.4239271962 7.8972140005

O 9.6311179460 7.7441852666 7.9008227904

O 7.7085495947 11.0661431578 7.9087352003

O 5.7925523528 14.3869246185 7.9015874938

O 3.8828731047 17.7018508695 7.8966364135

O 17.2776664809 1.1063202699 7.8351382446

O 15.3639619639 4.4247992696 7.8928713127

O 13.4502446450 7.7447720867 7.8925541038

O 11.5340051611 11.0647246319 7.8955078156

O 9.6160429644 14.3828329571 7.8947130349

O 7.6975229549 17.7019941230 7.8866548316

Ce -0.0000000000 0.0000000000 3.9115480740

Ce -1.9162593770 3.3190586010 3.9115480740

Ce -3.8325187540 6.6381172030 3.9115480740

Ce -5.7487781310 9.9571758040 3.9115480740

Ce -7.6650375080 13.2762344060 3.9115480740

Ce -9.5812968850 16.5952930070 3.9115480740

Ce 3.8325187540 0.0000000000 3.9115480740

Ce 1.9162593770 3.3190586010 3.9115480740

Ce -0.0000000000 6.6381172030 3.9115480740

Ce -1.9162593770 9.9571758040 3.9115480740

Ce -3.8325187540 13.2762344060 3.9115480740

Ce -5.7487781310 16.5952930070 3.9115480740

Ce 7.6650375080 -0.0000000000 3.9115480740

Ce 5.7487781310 3.3190586010 3.9115480740

Ce 3.8325187540 6.6381172030 3.9115480740

Ce 1.9162593770 9.9571758040 3.9115480740

Ce 0.0000000000 13.2762344060 3.9115480740

Ce -1.9162593770 16.5952930070 3.9115480740

Ce 11.4975562620 -0.0000000000 3.9115480740

Ce 9.5812968850 3.3190586010 3.9115480740

Ce 7.6650375080 6.6381172030 3.9115480740

Ce 5.7487781310 9.9571758040 3.9115480740

Ce 3.8325187540 13.2762344060 3.9115480740

Ce 1.9162593770 16.5952930070 3.9115480740

Ce 15.3300750160 -0.0000000000 3.9115480740

Ce 13.4138156390 3.3190586010 3.9115480740

Ce 11.4975562620 6.6381172030 3.9115480740

Ce 9.5812968850 9.9571758040 3.9115480740

Ce 7.6650375080 13.2762344060 3.9115480740

Ce 5.7487781310 16.5952930070 3.9115480740

Ce 0.0574300732 0.0186827673 13.4447362486

Ce -1.8684221771 3.3010344450 13.4540569531

Ce -3.7831071603 6.6419403626 13.4555680535

Ce -5.6696771130 9.9669525277 13.4652820915

Ce -7.5755678145 13.3091001690 13.4842135417

Ce -9.4875730122 16.6276655436 13.4742510220

Ce 3.8928008394 -0.0136132617 13.4663205851

Ce 1.9648956576 3.2453430804 13.4790805763

Ce 0.0007072580 6.6173883114 13.5089603023

Ce -1.9362189299 9.9911892051 13.4818968824

Ce -3.7976847344 13.3201811179 13.4615721222

Ce -5.6980721483 16.6267910768 13.4561425198

Ce 7.7309128949 -0.0079775604 13.4587964656

Ce 5.8380721274 3.2406617872 13.4907440211

Ce 3.8861804287 6.4975850555 13.4809358757

Ce 1.8320606370 10.0245312592 13.5076290093

Ce -0.0202464997 13.3496125071 13.4956403113

Ce -1.9058395038 16.6359900434 13.4518546426

Ce 11.5452525670 0.0285911576 13.4458341597

Ce 9.6502637996 3.3165801195 13.4748646670

Ce 7.7986129695 6.6245551827 13.5182822188

Ce 5.9211891933 10.0164404224 13.5765474152

Ce 3.8268266016 13.2480422297 13.4898159123

Ce 1.8670211180 16.6831167255 13.4248388393

Ce 15.3896927650 0.0048058819 13.4490209558

Ce 13.4714024493 3.3141070186 13.4576641377

Ce 11.5890133227 6.6401307327 13.4755810545

Ce 9.7450572101 10.0147744953 13.5263376293

Ce 7.7939317838 13.3590573340 13.6595666571

Ce 5.8953357118 16.6624712839 13.5316194378

Ce 1.9653730968 1.1029294675 10.2820264398

Ce 0.0518255353 4.4241783921 10.3074235702

Ce -1.8661188488 7.7470696187 10.3026264932

Ce -3.7902041225 11.0709950926 10.2882228503

Ce -5.7070451028 14.3916550306 10.2818125811

Ce -7.6168651040 17.6961183173 10.2715067348

Ce 5.7901119646 1.1022415918 10.2757899936

Ce 3.8900687768 4.4233537017 10.2903787619

Ce 1.9600881401 7.7509555108 10.3359203835

Ce 0.0273928501 11.0731549516 10.3049616379

Ce -1.8837955550 14.3942280201 10.2868359004

Ce -3.8012807122 17.7039713095 10.2632355398

Ce 9.6122402949 1.1071461039 10.2825859174

Ce 7.7147879844 4.4302061272 10.3097179154

Ce 5.8045379283 7.7644265216 10.3477383011

Ce 3.8602973130 11.0480134141 10.3277164643

Ce 1.9419440966 14.3791078298 10.3063947857

Ce 0.0218150141 17.7107195352 10.2492529205

Ce 13.4502454210 1.1007092104 10.2707584642

Ce 11.5486682823 4.4282059706 10.3019646647

Ce 9.6427251488 7.7613526036 10.3237903868

Ce 7.7142715593 11.0871484221 10.3482981158

Ce 5.7871278761 14.3783852752 10.3588445373

Ce 3.8803731486 17.6900078856 10.3018087671

Ce 17.2972461003 1.1050326489 10.2721143930

Ce 15.3855809325 4.4231805926 10.2839312951

Ce 13.4800982361 7.7475495057 10.2867402506

Ce 11.5601195430 11.0727178956 10.2977200985

Ce 9.6230043911 14.3812540070 10.3256270718

Ce 7.7137313607 17.6997093977 10.2883619570

Ce 0.0192087638 2.2160438412 7.0643309126

Ce -1.8946697865 5.5341991209 7.0717333723

Ce -3.8137945179 8.8510708058 7.0692072676

Ce -5.7366880833 12.1704826886 7.0636187849

Ce -7.6572290371 15.4853409017 7.0593310786

Ce -9.5687843649 18.8045670111 7.0546628989

Ce 3.8463265839 2.2158494892 7.0733000894

Ce 1.9322660040 5.5395769451 7.0903904976

Ce 0.0177471444 8.8529377507 7.0883637636

Ce -1.9080694157 12.1705303863 7.0737712343

Ce -3.8289889799 15.4885592164 7.0523438310

Ce -5.7454791548 18.8076071394 7.0444434059

Ce 7.6824887410 2.2175349750 7.0756710668

Ce 5.7873263211 5.5419240331 7.0917127362

Ce 3.8575425617 8.8529930502 7.0940769474

Ce 1.9301801175 12.1687436574 7.0904773352

Ce 0.0065814027 15.4841976117 7.0667623368

Ce -1.9150522700 18.8087264748 7.0444811714

Ce 11.5173701233 2.2131529788 7.0674082360

Ce 9.6147723046 5.5362492322 7.0876705516

Ce 7.6969664838 8.8562485952 7.0989000941

Ce 5.7704401573 12.1706438193 7.1045469183

Ce 3.8583034951 15.4817404320 7.0874291896

Ce 1.9400156896 18.8024890938 7.0591655713

Ce 15.3553508941 2.2114953287 7.0543232508

Ce 13.4385857302 5.5322079576 7.0696999480

Ce 11.5193415113 8.8525410987 7.0788483092

Ce 9.5991150299 12.1718450746 7.0861741287

Ce 7.6855521537 15.4806305264 7.0830261268

Ce 5.7731970622 18.8003364690 7.0593486641

Au -0.6637314530 6.3572429707 17.1448638624

Au 6.6532931179 7.3506956372 18.8573647827

Au 3.2027966196 15.4926673556 16.5851085600

Au 0.5587007019 11.4130023674 20.9887775074

Au -0.2610108587 10.6810528125 18.2523876565

Au 4.9912860531 9.6573293197 18.5344046911

Au 3.7144537656 8.7589562298 15.9672164465

Au 2.3982017567 6.0365573743 21.0451951933

Au 2.2332124862 6.2092100559 16.5165038800

Au 5.2726303590 6.1680641556 16.5008635986

Au 3.7752197598 12.3575882967 18.6489304943

Au 4.0317358918 8.8249793522 21.0514784278

Au 6.7524234676 8.9037655159 16.4784626662

Au 5.1376752439 11.3811597170 16.4370103799

Au 1.2162833768 7.2562781194 18.9534491965

Au 2.2532700584 10.0954432787 19.3171611942

Au 2.0562838616 11.1893460169 16.6824464164

Au 0.7868451616 8.6475579083 16.5663477486

Au 3.9494507663 6.9779575753 18.7271061090

Au 1.0446723614 8.6517354773 21.3754089472

C -2.5420315969 5.7188559605 17.4837420340

O -3.6303221309 5.8244776560 17.8377667130

C 8.2407630265 7.1321981607 20.0230091576

O 9.0091132878 7.2999750170 20.8612107937

C 2.5624829459 15.6563290005 18.3861711782

O 2.1168900261 15.8533645856 19.4260814498

C -0.3773861001 8.0835395424 22.6922646676

O -1.4111328236 7.6714556276 22.9899412802

C 5.1542965662 8.4609604586 22.6940725837

O 6.0979720276 8.3355589940 23.3414954280

C -0.6729503130 12.4404026897 22.1775462119

O -1.5621573151 12.5429785316 22.9003589851

C 2.6211043827 4.7411604451 22.5732461885

O 2.2724427720 4.0568314932 23.4295802811

C -2.2707480322 10.5334692224 18.1793160441

O -3.2656478954 9.9602382698 18.1060085133

(3) A bent intermediate; (Cofig. iii)

O 0.0074561671 -0.0004760889 6.2284353644

O -1.9104832121 3.3230923698 6.2302908089

O -3.8260923477 6.6379011647 6.2337820576

O -5.7423262061 9.9571685288 6.2339015726

O -7.6592641461 13.2745918114 6.2324828779

O -9.5793826027 16.5932695800 6.2289750828

O 3.8250763601 0.0066078577 6.2285279717

O 1.9153392132 3.3228502980 6.2796778776

O -0.0030593900 6.6394888397 6.2809321189

O -1.9207333342 9.9583278411 6.2810214845

O -3.8371157330 13.2757590149 6.2777404856

O -5.7542723976 16.5919382379 6.2268346985

O 7.6542232601 0.0064805556 6.2324126341

O 5.7834211779 3.3239575549 6.2845981998

O 3.8738048480 6.6393554017 6.2905507950

O 1.9404711197 9.9554489955 6.2724584489

O -0.0146018276 13.2758014629 6.2787672734

O -1.8838099055 16.5927100442 6.2317736004

O 11.5254574420 0.0012677184 6.2868236973

O 9.6138201485 3.3183439042 6.2851318881

O 7.6966136206 6.6376214632 6.2875158794

O 5.7770090772 9.9579266241 6.2929441555

O 3.8594105549 13.2767677003 6.2914627819

O 1.9445794945 16.5959572135 6.2896138716

O 15.3439602044 -0.0032498796 6.2320659488

O 13.4305172311 3.3185374729 6.2331526432

O 11.5182921457 6.6376556794 6.2835545343

O 9.6008620297 9.9570810665 6.2861526271

O 7.6817285726 13.2737868015 6.2853714971

O 5.7644445210 16.5932914233 6.2356823568

O 0.0510001035 2.2196297716 9.4858010392

O -1.8617270624 5.5337489342 9.4943647397

O -3.7761642171 8.8524888848 9.4934429801

O -5.6904566826 12.1723366181 9.4918089768

O -7.6127849075 15.4921768745 9.4866061523

O -9.5304957768 18.8093399455 9.4887930801

O 3.8672192810 2.2178370005 9.4893853605

O 1.9506081235 5.5314542368 9.4964284846

O 0.0296977763 8.8465698933 9.4930064335

O -1.8820165935 12.1722085550 9.4954323375

O -3.7947581371 15.4894670598 9.4883935661

O -5.7115706587 18.8065682777 9.4224832452

O 7.6900391550 2.2166892877 9.4913649922

O 5.8342792944 5.5313404307 9.4937334901

O 3.8534184918 8.8512035854 9.4999829373

O 1.9333892101 12.1737328421 9.4950321202

O 0.0188798338 15.4875227717 9.4960184686

O -1.8977198736 18.8088632203 9.4853112378

O 11.5045541598 2.2122778308 9.4897922977

O 9.6580654821 5.5358542805 9.5025958685

O 7.7480676005 8.8495945687 9.5037838802

O 5.7481355363 12.1717215275 9.5012300959

O 3.8380693105 15.4827049401 9.4926980544

O 1.9269112996 18.8074470789 9.4337760955

O 15.3925899111 2.2138607509 9.4898883498

O 13.4773764423 5.5321666185 9.4932536981

O 11.5619151458 8.8469299105 9.4946704933

O 9.6455949047 12.1668825720 9.4957007756

O 7.7253320421 15.4925596004 9.4965409279

O 5.8114150303 18.8144929174 9.4878521144

O 1.9162593770 1.1063528670 3.1292384590

O -0.0000000000 4.4254114690 3.1292384590

O -1.9162593770 7.7444700700 3.1292384590

O -3.8325187540 11.0635286720 3.1292384590

O -5.7487781310 14.3825872730 3.1292384590

O -7.6650375080 17.7016458750 3.1292384590

O 5.7487781310 1.1063528670 3.1292384590

O 3.8325187540 4.4254114690 3.1292384590

O 1.9162593770 7.7444700700 3.1292384590

O 0.0000000000 11.0635286720 3.1292384590

O -1.9162593770 14.3825872730 3.1292384590

O -3.8325187540 17.7016458750 3.1292384590

O 9.5812968850 1.1063528670 3.1292384590

O 7.6650375080 4.4254114690 3.1292384590

O 5.7487781310 7.7444700700 3.1292384590

O 3.8325187540 11.0635286720 3.1292384590

O 1.9162593770 14.3825872730 3.1292384590

O 0.0000000000 17.7016458750 3.1292384590

O 13.4138156390 1.1063528670 3.1292384590

O 11.4975562620 4.4254114690 3.1292384590

O 9.5812968850 7.7444700700 3.1292384590

O 7.6650375080 11.0635286720 3.1292384590

O 5.7487781310 14.3825872730 3.1292384590

O 3.8325187540 17.7016458750 3.1292384590

O 17.2463343930 1.1063528670 3.1292384590

O 15.3300750160 4.4254114690 3.1292384590

O 13.4138156390 7.7444700700 3.1292384590

O 11.4975562620 11.0635286720 3.1292384590

O 9.5812968850 14.3825872730 3.1292384590

O 7.6650375080 17.7016458750 3.1292384590

O 2.0029972049 1.1045929077 12.6151901930

O 0.0199446330 4.4273487766 12.6782410782

O -1.8925876872 7.7365542327 12.6785032317

O -3.8087249240 11.0681348034 12.6777140367

O -5.6888537644 14.4382921722 12.6165503236

O -7.5997351162 17.7614900209 12.6061556919

O 5.8313415360 1.1107570310 12.6780590437

O 3.9541564827 4.3587639117 12.6836499434

O 1.9963906415 7.7459319600 12.7773101037

O -0.0348314547 11.1285684672 12.6913247600

O -1.7929720286 14.4669776934 12.6955856608

O -3.7905371042 17.7617735189 12.6093246260

O 9.6487293419 1.1043838850 12.6247058981

O 7.7235855724 4.4291029230 12.6872909835

O 5.8214800984 7.7387216391 12.7797465463

O 3.8261985917 11.0644822502 12.7655837616

O 1.8264343812 14.5260135685 12.7722116138

O 0.0330687946 17.6262808317 12.7014362174

O 13.4643495633 1.1129851838 12.6125455870

O 11.5556834944 4.4237023446 12.6798156406

O 9.6404423886 7.7454143250 12.6855210906

O 7.8069985586 11.0555768477 12.6946747463

O 5.7136806011 14.3815662860 12.6891487382

O 3.8736749710 17.7067105394 12.6836288746

O 17.2850431772 1.1225992249 12.6041889103

O 15.3785574927 4.4247190758 12.6657575042

O 13.5242357625 7.7450213869 12.6209040761

O 11.6236172191 11.0646860855 12.6146344599

O 9.7164529149 14.3939705235 12.6176038945

O 7.7547094605 17.7746706737 12.6053816517

O -0.0000000000 2.2127057340 4.6938576890

O -1.9162593770 5.5317643360 4.6938576890

O -3.8325187540 8.8508229370 4.6938576890

O -5.7487781310 12.1698815390 4.6938576890

O -7.6650375080 15.4889401400 4.6938576890

O -9.5812968850 18.8079987420 4.6938576890

O 3.8325187540 2.2127057340 4.6938576890

O 1.9162593770 5.5317643360 4.6938576890

O 0.0000000000 8.8508229370 4.6938576890

O -1.9162593770 12.1698815390 4.6938576890

O -3.8325187540 15.4889401400 4.6938576890

O -5.7487781310 18.8079987420 4.6938576890

O 7.6650375080 2.2127057340 4.6938576890

O 5.7487781310 5.5317643360 4.6938576890

O 3.8325187540 8.8508229370 4.6938576890

O 1.9162593770 12.1698815390 4.6938576890

O 0.0000000000 15.4889401400 4.6938576890

O -1.9162593770 18.8079987420 4.6938576890

O 11.4975562620 2.2127057340 4.6938576890

O 9.5812968850 5.5317643360 4.6938576890

O 7.6650375080 8.8508229370 4.6938576890

O 5.7487781310 12.1698815390 4.6938576890

O 3.8325187540 15.4889401400 4.6938576890

O 1.9162593770 18.8079987420 4.6938576890

O 15.3300750160 2.2127057340 4.6938576890

O 13.4138156390 5.5317643360 4.6938576890

O 11.4975562620 8.8508229370 4.6938576890

O 9.5812968850 12.1698815390 4.6938576890

O 7.6650375080 15.4889401400 4.6938576890

O 5.7487781310 18.8079987420 4.6938576890

O 0.0481555426 0.0564772748 11.0964128670

O -1.8456372510 3.3242286767 11.1039471172

O -3.7518314173 6.6316496948 11.1012035592

O -5.6643203715 9.9544020951 11.0981766654

O -7.5814515446 13.2727880874 11.0972682629

O -9.5041462322 16.5989426855 11.0979091007

O 3.9048043664 0.0061255269 11.0946954689

O 1.9794951880 3.3287105977 11.1605600447

O 0.0666729890 6.6423591368 11.1777780681

O -1.8620894534 9.9540384252 11.1630206263

O -3.7592939948 13.2805297470 11.1732019936

O -5.6862112378 16.6070949656 11.0942061519

O 7.7088720752 0.0045943352 11.0966723563

O 5.8060214291 3.3156948457 11.1045698799

O 3.8870213082 6.6333417201 11.0879391774

O 1.8940335322 9.9583815746 11.0953233499

O 0.0480031500 13.2747231534 11.1611287485

O -1.8816005739 16.6056107869 11.0994729143

O 11.5370169941 -0.0146217331 11.1089861562

O 9.6209517306 3.3235403590 11.1597557510

O 7.7080869194 6.6413835252 11.1743288440

O 5.7880882343 9.9607579166 11.0782708335

O 3.7826676866 13.2795378259 11.1853216165

O 1.9601949074 16.6168197716 11.0925573423

O 15.3548272312 -0.0057644120 11.0956009541

O 13.4427735618 3.3201829554 11.1003468686

O 11.5834837043 6.6374000006 11.1642954612

O 9.6776338417 9.9567765180 11.1608576430

O 7.6886722017 13.2792877120 11.0893663636

O 5.7768655292 16.6006521058 11.0908864281

O 0.0534398955 2.2083639585 14.2806103875

O -1.8668047921 5.5297802214 14.3619460124

O -3.7843102673 8.8519306255 14.2945539969

O -5.6509785218 12.2395752232 14.2871944629

O -7.5678093478 15.5585916945 14.2862651276

O -9.4878522399 18.8743191215 14.2871340171

O 3.8750194757 2.2040491329 14.2930010386

O 1.8595674110 5.3791666707 14.3810311826

O -0.1401609499 8.8451672292 14.3754007450

O -1.9271546403 12.2341778829 14.2931409396

O -3.8328231204 15.5603365698 14.2768774318

O -5.7063321285 18.8116220109 14.2868521027

O 7.7660789387 2.2066830117 14.2898041959

O 5.9457236370 5.3860554293 14.5493452158

O 1.8532367473 12.3157099752 14.4639368964

O -0.2533906130 15.4905110395 14.8150109044

O -1.8959103010 18.8154669144 14.2822210373

O 11.5807297358 2.2048711419 14.2840700907

O 9.6702598691 5.5305358048 14.2907177312

O 7.9643247560 8.7749627622 14.5494495545

O 5.6624924766 12.3239418633 14.5437533240

O 3.9166413233 15.4825045079 14.5458915915

O 1.9601048352 18.8783869018 14.2817705989

O 15.3971193973 2.2130165786 14.2838861226

O 13.4818235411 5.5336744806 14.2864376150

O 11.6498454439 8.8492635116 14.2852857479

O 9.8708212532 12.0998603444 14.3720232298

O 7.9141793127 15.6422597053 14.3765655849

O 5.8224157843 18.9602533863 14.2944824176

O 1.9353859819 1.1047043228 7.8873310207

O 0.0172291966 4.4255596533 7.8946983822

O -1.8998214655 7.7424626927 7.8953481235

O -3.8163279246 11.0618395552 7.8960243745

O -5.7314763377 14.3840763786 7.8865988417

O -7.6513380178 17.7004812378 7.8274888971

O 5.7519903727 1.1080368497 7.8910459479

O 3.8433126900 4.4268201783 7.8989602935

O 1.9223597652 7.7437701889 7.8991839864

O 0.0040650014 11.0625851976 7.8965545043

O -1.9131678561 14.3852159037 7.8968162904

O -3.8235154900 17.6986316437 7.8264436683

O 9.5742847192 1.1024368626 7.8884626687

O 7.7282723594 4.4254441402 7.8997718196

O 5.8048674674 7.7473249147 7.9107454999

O 3.8241806839 11.0627550613 7.9017212759

O 1.9077085270 14.3810894944 7.8986583212

O -0.0121095716 17.6984140386 7.8944569500

O 13.4613513453 1.1131953209 7.8918862667

O 11.5498648952 4.4237342763 7.8982575390

O 9.6311227295 7.7440403400 7.9002658649

O 7.7088975669 11.0664927114 7.9049733789

O 5.7924799600 14.3871576978 7.9002870941

O 3.8838091565 17.7026377061 7.8965953466

O 17.2780082982 1.1065396116 7.8347888896

O 15.3642796719 4.4245678556 7.8928776163

O 13.4505609984 7.7448645048 7.8924206298

O 11.5340680015 11.0643844782 7.8927248375

O 9.6162706269 14.3833970318 7.8909320061

O 7.6973881652 17.7025103578 7.8864637544

Ce -0.0000000000 0.0000000000 3.9115480740

Ce -1.9162593770 3.3190586010 3.9115480740

Ce -3.8325187540 6.6381172030 3.9115480740

Ce -5.7487781310 9.9571758040 3.9115480740

Ce -7.6650375080 13.2762344060 3.9115480740

Ce -9.5812968850 16.5952930070 3.9115480740

Ce 3.8325187540 -0.0000000000 3.9115480740

Ce 1.9162593770 3.3190586010 3.9115480740

Ce 0.0000000000 6.6381172030 3.9115480740

Ce -1.9162593770 9.9571758040 3.9115480740

Ce -3.8325187540 13.2762344060 3.9115480740

Ce -5.7487781310 16.5952930070 3.9115480740

Ce 7.6650375080 0.0000000000 3.9115480740

Ce 5.7487781310 3.3190586010 3.9115480740

Ce 3.8325187540 6.6381172030 3.9115480740

Ce 1.9162593770 9.9571758040 3.9115480740

Ce 0.0000000000 13.2762344060 3.9115480740

Ce -1.9162593770 16.5952930070 3.9115480740

Ce 11.4975562620 0.0000000000 3.9115480740

Ce 9.5812968850 3.3190586010 3.9115480740

Ce 7.6650375080 6.6381172030 3.9115480740

Ce 5.7487781310 9.9571758040 3.9115480740

Ce 3.8325187540 13.2762344060 3.9115480740

Ce 1.9162593770 16.5952930070 3.9115480740

Ce 15.3300750160 -0.0000000000 3.9115480740

Ce 13.4138156390 3.3190586010 3.9115480740

Ce 11.4975562620 6.6381172030 3.9115480740

Ce 9.5812968850 9.9571758040 3.9115480740

Ce 7.6650375080 13.2762344060 3.9115480740

Ce 5.7487781310 16.5952930070 3.9115480740

Ce 0.0626052817 0.0176173737 13.4483647575

Ce -1.8558534588 3.3148669601 13.4557053308

Ce -3.7842133420 6.6334365929 13.4558228182

Ce -5.6754932199 9.9619158655 13.4542400667

Ce -7.5773313063 13.2943475735 13.4625034618

Ce -9.4989356626 16.6271162728 13.4702557167

Ce 3.8835637009 -0.0017259182 13.4723423035

Ce 1.9644757132 3.2535570026 13.4825004258

Ce 0.0137367160 6.6256732401 13.5172966035

Ce -1.9485299565 9.9589070691 13.4894910774

Ce -3.8066358176 13.3165690330 13.4913529184

Ce -5.7407613041 16.6301306924 13.4590877699

Ce 7.7143903480 0.0207347154 13.4621035150

Ce 5.8311746746 3.2555650283 13.4919153881

Ce 3.8862513414 6.4980707409 13.4987293401

Ce 1.8344382846 10.0239944184 13.5251400371

Ce -0.0201793983 13.2299667442 13.4893758149

Ce -2.0137778445 16.6822201860 13.4546979737

Ce 11.5519430349 0.0223660898 13.4542916153

Ce 9.6486067696 3.3225345839 13.4753142474

Ce 7.7886338512 6.6322571186 13.5228873846

Ce 5.9069824117 10.0382556171 13.4918349830

Ce 3.8508510079 13.3388105842 13.5490610228

Ce 2.0412955394 16.7432361080 13.4250947640

Ce 15.4146712170 0.0489450736 13.4583660265

Ce 13.4674683069 3.3166434262 13.4550471123

Ce 11.5840666706 6.6406981614 13.4761284253

Ce 9.7425484994 10.0068279246 13.5145385170

Ce 7.7942971434 13.3574262187 13.5875464155

Ce 5.8958946939 16.6593541759 13.5830579536

Ce 1.9651879160 1.1041974236 10.2832024688

Ce 0.0539107775 4.4268990798 10.3078125647

Ce -1.8642845573 7.7444818418 10.3024159718

Ce -3.7887622810 11.0671073449 10.2987695006

Ce -5.7114305552 14.3928239709 10.2906264995

Ce -7.6245823634 17.7029775138 10.2700220403

Ce 5.7854325826 1.1069292787 10.2750386694

Ce 3.8883724459 4.4262693661 10.2948428519

Ce 1.9647699521 7.7513438821 10.3445284781

Ce 0.0309486789 11.0635895435 10.2854451045

Ce -1.8785323244 14.3938350708 10.3093381847

Ce -3.8087984898 17.7086532097 10.2475336347

Ce 9.6075046345 1.1081071653 10.2859314533

Ce 7.7143855034 4.4329808668 10.3119095280

Ce 5.8006192416 7.7599980086 10.3453210245

Ce 3.8501599511 11.0625878388 10.3515210749

Ce 1.9352033201 14.3903858103 10.3205837065

Ce 0.0263498563 17.6987135220 10.2769510603

Ce 13.4580793967 1.0997291125 10.2738937936

Ce 11.5482177346 4.4299377016 10.3014766422

Ce 9.6400688902 7.7627217850 10.3240985887

Ce 7.7198745655 11.0875678286 10.3297423409

Ce 5.7890131176 14.3879043057 10.3633542485

Ce 3.8996773644 17.6950636845 10.2748253163

Ce 17.3005241955 1.1082693642 10.2713882883

Ce 15.3863472296 4.4237781397 10.2832324608

Ce 13.4795951527 7.7463829544 10.2856042430

Ce 11.5580011629 11.0690223741 10.2878003166

Ce 9.6251529576 14.3817761489 10.3099797552

Ce 7.7122483158 17.6960716697 10.2961722132

Ce 0.0202916160 2.2177483399 7.0645364313

Ce -1.8946012066 5.5341207733 7.0713711885

Ce -3.8133881135 8.8525310932 7.0699274654

Ce -5.7359042881 12.1701923764 7.0704464798

Ce -7.6545546504 15.4857316123 7.0590960765

Ce -9.5698256135 18.8038784252 7.0553472033

Ce 3.8453353730 2.2174462216 7.0736082326

Ce 1.9338830082 5.5410826732 7.0915092114

Ce 0.0182988002 8.8492095819 7.0852355862

Ce -1.9092171033 12.1716842956 7.0794454349

Ce -3.8226279442 15.4850803550 7.0604892336

Ce -5.7467379147 18.8100561388 7.0405712915

Ce 7.6825031178 2.2178406291 7.0762456290

Ce 5.7860259746 5.5418339641 7.0912704573

Ce 3.8563539566 8.8558375140 7.0987068747

Ce 1.9332901484 12.1687179485 7.0904872001

Ce 0.0115925114 15.4838850119 7.0739278177

Ce -1.9055156672 18.8088107949 7.0591997193

Ce 11.5175248023 2.2153446267 7.0757712039

Ce 9.6135211054 5.5366451562 7.0880603221

Ce 7.6960666382 8.8548671602 7.0981168370

Ce 5.7677270069 12.1697906795 7.1074072732

Ce 3.8595461570 15.4777567322 7.0885042244

Ce 1.9385453719 18.8026155171 7.0739062955

Ce 15.3555526657 2.2110016461 7.0538138472

Ce 13.4393670716 5.5328205935 7.0690704454

Ce 11.5190342687 8.8514133129 7.0780352484

Ce 9.5991437128 12.1723290509 7.0808892163

Ce 7.6844157378 15.4811940735 7.0822045686

Ce 5.7774292196 18.8023376536 7.0541409874

Au -0.6629080062 6.3870235392 17.1529797963

Au 6.6686631133 7.3817959556 18.8481611974

Au 2.1816622847 15.2815260732 16.0218618719

Au 0.5522063903 11.3984193016 20.9826613621

Au -0.2764496574 10.7174421218 18.2142287249

Au 5.0065284827 9.6513814424 18.5198801981

Au 3.7288989021 8.7617431528 15.9468616310

Au 2.4574874047 6.0334720800 21.0626014034

Au 2.2496246879 6.2056940873 16.5352876398

Au 5.2481845120 6.1416845853 16.5098517203

Au 3.8256147680 12.2756769997 18.7224338569

Au 4.0341620818 8.8209008358 21.0452412582

Au 6.7268319989 8.9001503356 16.4345400927

Au 5.1049058896 11.3986179675 16.4206936770

Au 1.2250944919 7.2045193892 18.9808190000

Au 2.2257075896 10.0574890952 19.3127219410

Au 2.0705876477 11.2075494637 16.7109819194

Au 0.8270927961 8.6593667577 16.6124138840

Au 3.9633745572 6.9807743027 18.7404640053

Au 1.0558022312 8.6400453256 21.3898098146

C -2.5427506697 5.7388828577 17.4740579606

O -3.6298184146 5.8246947624 17.8373294932

C 8.2596108665 7.1321630277 20.0042194822

O 9.0089993045 7.2996705058 20.8600997204

C 0.1252614246 15.4053794620 16.1262040018

O -0.6498510870 15.4162440665 17.0513631987

C -0.3701811465 8.0624834762 22.6939811905

O -1.4119069975 7.6712039308 22.9903558876

C 5.1646441341 8.4973522156 22.6887878120

O 6.0984349958 8.3353707874 23.3417546541

C -0.6874468497 12.4268467537 22.1620032719

O -1.5616671607 12.5424171252 22.8996956345

C 2.6516119765 4.7516049410 22.5989824840

O 2.2723066182 4.0552455803 23.4320202647

C -2.2841356089 10.5561083127 18.1799380044

O -3.2662476943 9.9608260418 18.1057378103

(4) CO_2_ desorbing into the gas phase (Cofig. iv)

O 0.0075377622 -0.0003074238 6.2283149638

O -1.9103994715 3.3230647145 6.2301993073

O -3.8263269830 6.6376399493 6.2333633782

O -5.7428392455 9.9571410727 6.2329516724

O -7.6591000001 13.2745817403 6.2325409789

O -9.5794754003 16.5933624151 6.2290197136

O 3.8251396455 0.0067277959 6.2283534606

O 1.9155117572 3.3229688988 6.2794589677

O -0.0032436208 6.6386185663 6.2803088437

O -1.9215869232 9.9580470756 6.2354232605

O -3.8371818300 13.2766268677 6.2779301587

O -5.7540450270 16.5919983680 6.2269964032

O 7.6540625472 0.0068615326 6.2324927247

O 5.7838000715 3.3236831080 6.2845775849

O 3.8736485889 6.6394151608 6.2900768727

O 1.9127022719 9.9567406789 6.2512011366

O -0.0148166287 13.2765710274 6.2401652638

O -1.8835906792 16.5926789801 6.2315252232

O 11.5254616396 0.0014624244 6.2881654348

O 9.6141584828 3.3182120066 6.2850101113

O 7.6966945592 6.6375788035 6.2873874386

O 5.7767569623 9.9575381430 6.2923983405

O 3.8590901674 13.2770170455 6.2915045697

O 1.9443247788 16.5955815464 6.2897032997

O 15.3442067541 -0.0030941023 6.2319068666

O 13.4302201896 3.3186620308 6.2330457584

O 11.5182992681 6.6374347205 6.2836335849

O 9.6009870966 9.9572035380 6.2860408626

O 7.6819258782 13.2737254100 6.2853755527

O 5.7643048647 16.5933537454 6.2360630853

O 0.0513639156 2.2199540697 9.4857776047

O -1.8615600392 5.5330028660 9.4931481485

O -3.7784486556 8.8526467091 9.4905786155

O -5.6871519248 12.1753138779 9.4931295323

O -7.6125497403 15.4923148611 9.4872540099

O -9.5306520810 18.8094777554 9.4891244949

O 3.8676048840 2.2177937675 9.4893144858

O 1.9513784895 5.5306567359 9.4957224009

O 0.0344008861 8.8420531947 9.4899280121

O -1.8877876529 12.1737221202 9.4861062846

O -3.7944222351 15.4926711766 9.4828705188

O -5.7131442598 18.8077509790 9.4212971925

O 7.6922489064 2.2150992129 9.4927230320

O 5.8357707405 5.5298521212 9.4920280773

O 3.8549390835 8.8461249565 9.4937559389

O 1.9352751066 12.1747630154 9.4909517404

O 0.0202430065 15.4894269477 9.5006731968

O -1.8931283285 18.8147307412 9.4846733487

O 11.5043297483 2.2049604017 9.4952070667

O 9.6586346158 5.5357726509 9.5022185822

O 7.7522526420 8.8476311509 9.4999731147

O 5.7508776418 12.1754172865 9.4981793433

O 3.8365801811 15.4807265900 9.4918043640

O 1.9238359136 18.8110343371 9.4248899657

O 15.3916617709 2.2139707539 9.4896923799

O 13.4769913205 5.5318525773 9.4926850426

O 11.5613995504 8.8465847710 9.4936107539

O 9.6471544773 12.1671509950 9.4949328390

O 7.7246086911 15.4929616809 9.4973017120

O 5.8131567089 18.8155874071 9.4869984131

O 1.9162593770 1.1063528670 3.1292384590

O -0.0000000000 4.4254114690 3.1292384590

O -1.9162593770 7.7444700700 3.1292384590

O -3.8325187540 11.0635286720 3.1292384590

O -5.7487781310 14.3825872730 3.1292384590

O -7.6650375080 17.7016458750 3.1292384590

O 5.7487781310 1.1063528670 3.1292384590

O 3.8325187540 4.4254114690 3.1292384590

O 1.9162593770 7.7444700700 3.1292384590

O -0.0000000000 11.0635286720 3.1292384590

O -1.9162593770 14.3825872730 3.1292384590

O -3.8325187540 17.7016458750 3.1292384590

O 9.5812968850 1.1063528670 3.1292384590

O 7.6650375080 4.4254114690 3.1292384590

O 5.7487781310 7.7444700700 3.1292384590

O 3.8325187540 11.0635286720 3.1292384590

O 1.9162593770 14.3825872730 3.1292384590

O 0.0000000000 17.7016458750 3.1292384590

O 13.4138156390 1.1063528670 3.1292384590

O 11.4975562620 4.4254114690 3.1292384590

O 9.5812968850 7.7444700700 3.1292384590

O 7.6650375080 11.0635286720 3.1292384590

O 5.7487781310 14.3825872730 3.1292384590

O 3.8325187540 17.7016458750 3.1292384590

O 17.2463343930 1.1063528670 3.1292384590

O 15.3300750160 4.4254114690 3.1292384590

O 13.4138156390 7.7444700700 3.1292384590

O 11.4975562620 11.0635286720 3.1292384590

O 9.5812968850 14.3825872730 3.1292384590

O 7.6650375080 17.7016458750 3.1292384590

O 2.0020001524 1.1039467087 12.6155549502

O 0.0204471640 4.4266865095 12.6782650371

O -1.8915414466 7.7364196300 12.6729060415

O -3.8101329747 11.0677665960 12.6111670270

O -5.6900753410 14.4353489848 12.6160551984

O -7.6013871060 17.7607129656 12.6064924024

O 5.8311810910 1.1104266014 12.6767801975

O 3.9583333978 4.3570097726 12.6838524084

O 2.0024176847 7.7351727179 12.7764295197

O -0.0026103322 11.0599472723 12.6834514702

O -1.7357900536 14.5185575309 12.7711502592

O -3.7945922016 17.7653738487 12.6112884389

O 9.6505302840 1.1043035139 12.6201947070

O 7.7245789236 4.4277418325 12.6874081109

O 5.8263424766 7.7406359276 12.7779053097

O 3.8661311051 11.0057387427 12.7005890635

O 1.7685095404 14.4598618060 12.6923434706

O 0.0492535663 17.5586311346 12.7689066044

O 13.4664284695 1.1169094406 12.6155347429

O 11.5556986910 4.4236416988 12.6797573162

O 9.6420715593 7.7456262143 12.6852271347

O 7.8060729708 11.0557520055 12.6969483135

O 5.7200361478 14.3755190666 12.6885449464

O 3.8540077615 17.7738997887 12.6745002460

O 17.2852327944 1.1239432914 12.6041974404

O 15.3788499239 4.4249015951 12.6646065961

O 13.5256606046 7.7447859121 12.6199288749

O 11.6237356550 11.0656238613 12.6149587908

O 9.7132502753 14.3901580880 12.6248805143

O 7.7492894961 17.7732662230 12.6041863068

O 0.0000000000 2.2127057340 4.6938576890

O -1.9162593770 5.5317643360 4.6938576890

O -3.8325187540 8.8508229370 4.6938576890

O -5.7487781310 12.1698815390 4.6938576890

O -7.6650375080 15.4889401400 4.6938576890

O -9.5812968850 18.8079987420 4.6938576890

O 3.8325187540 2.2127057340 4.6938576890

O 1.9162593770 5.5317643360 4.6938576890

O 0.0000000000 8.8508229370 4.6938576890

O -1.9162593770 12.1698815390 4.6938576890

O -3.8325187540 15.4889401400 4.6938576890

O -5.7487781310 18.8079987420 4.6938576890

O 7.6650375080 2.2127057340 4.6938576890

O 5.7487781310 5.5317643360 4.6938576890

O 3.8325187540 8.8508229370 4.6938576890

O 1.9162593770 12.1698815390 4.6938576890

O 0.0000000000 15.4889401400 4.6938576890

O -1.9162593770 18.8079987420 4.6938576890

O 11.4975562620 2.2127057340 4.6938576890

O 9.5812968850 5.5317643360 4.6938576890

O 7.6650375080 8.8508229370 4.6938576890

O 5.7487781310 12.1698815390 4.6938576890

O 3.8325187540 15.4889401400 4.6938576890

O 1.9162593770 18.8079987420 4.6938576890

O 15.3300750160 2.2127057340 4.6938576890

O 13.4138156390 5.5317643360 4.6938576890

O 11.4975562620 8.8508229370 4.6938576890

O 9.5812968850 12.1698815390 4.6938576890

O 7.6650375080 15.4889401400 4.6938576890

O 5.7487781310 18.8079987420 4.6938576890

O 0.0476761861 0.0569040562 11.0964691418

O -1.8451608735 3.3247081325 11.1037591830

O -3.7518216459 6.6315884417 11.1007292512

O -5.6679922661 9.9524508836 11.0947431556

O -7.5802645865 13.2732573181 11.0973678444

O -9.5049956993 16.5978245112 11.0984224231

O 3.9047111986 0.0062641243 11.0944519216

O 1.9799721734 3.3294681560 11.1613379272

O 0.0673596522 6.6416896539 11.1771771420

O -1.8577580270 9.9529302756 11.1054261810

O -3.7211856173 13.3387752682 11.1789401035

O -5.6866683785 16.6075200224 11.0943697535

O 7.7089459649 0.0048086877 11.0964968574

O 5.8063087588 3.3150459028 11.1039419455

O 3.8913523595 6.6316077033 11.0872048396

O 1.9468980378 9.9493206177 11.0864891498

O 0.0029882618 13.2169237031 11.0994245355

O -1.9050616168 16.6512759346 11.0975218764

O 11.5675159194 -0.0712415306 11.1722644355

O 9.6221826379 3.3229221359 11.1582232934

O 7.7096695436 6.6411161052 11.1735953884

O 5.8003060066 9.9558391526 11.0244778169

O 3.7846332069 13.2789490349 11.1827999263

O 1.9886937494 16.6570036289 11.0950170753

O 15.3546978427 0.0002541232 11.0901884527

O 13.4425176316 3.3199727182 11.0999805676

O 11.5843275042 6.6369817318 11.1646896397

O 9.6792632561 9.9567590714 11.1617807850

O 7.6907389468 13.2778078991 11.0919421287

O 5.7711056832 16.6010015194 11.0921644787

O 0.0529921911 2.2088726704 14.2813582570

O -1.8667170155 5.5300647376 14.3617431780

O -3.7836363057 8.8549439605 14.2905966869

O -5.6451372366 12.2387726853 14.2843086186

O -7.5672522537 15.5572389508 14.2869036568

O -9.4889882283 18.8740765334 14.2877204475

O 3.8759280264 2.2040688160 14.2932253969

O 1.8630690346 5.3785997784 14.3810548839

O -0.1368572796 8.8404806341 14.3757349569

O -1.9621042038 12.1700257151 14.2828042259

O -3.8365304542 15.5568427776 14.2813435866

O -5.7068583203 18.8115728763 14.2876923784

O 7.7664506334 2.2055457423 14.2902426367

O 5.9468295878 5.3857294240 14.5488446983

O 1.8953658126 12.2535980429 14.3874860717

O -1.9998220035 17.1242165600 17.3887502480

O -1.8959495801 18.8260847919 14.2819198440

O 11.5811273435 2.2042182718 14.2860986951

O 9.6712720618 5.5306443167 14.2910183923

O 7.9665133021 8.7742658811 14.5501390466

O 5.6577519756 12.3218408903 14.5458773053

O 3.7460960315 15.4866692652 14.2966107190

O 1.9613437029 18.8808868154 14.2890950551

O 15.3972588783 2.2170697222 14.2861061225

O 13.4823802699 5.5344909339 14.2864278785

O 11.6509256090 8.8499316559 14.2836327339

O 9.8699942706 12.0989354744 14.3751634351

O 7.9079984887 15.6414196714 14.3777030481

O 5.8203562495 18.9595646411 14.2962193502

O 1.9353713834 1.1046969723 7.8873577015

O 0.0174402456 4.4257013908 7.8945009067

O -1.9005485969 7.7445631684 7.8926382745

O -3.8149355204 11.0600414926 7.8872812610

O -5.7313567895 14.3841133523 7.8860934873

O -7.6515380711 17.7003857582 7.8272172749

O 5.7520236353 1.1085704559 7.8912099713

O 3.8432425653 4.4267487919 7.8987819224

O 1.9225403079 7.7447364314 7.8963783877

O 0.0024233774 11.0623129891 7.8924234062

O -1.9109579000 14.3850618674 7.8967368997

O -3.8233552467 17.6996160750 7.8256826045

O 9.5749461485 1.1029768808 7.8886490137

O 7.7284702249 4.4249253201 7.8996193878

O 5.8048522414 7.7473608851 7.9083001807

O 3.8215973585 11.0608326404 7.8954719202

O 1.9041895069 14.3804223277 7.8948149799

O -0.0128124704 17.6973774328 7.8959203881

O 13.4611108392 1.1143265317 7.8921760628

O 11.5498488470 4.4227519538 7.8986717910

O 9.6316295523 7.7438681237 7.8995801086

O 7.7094737250 11.0663676569 7.9041893703

O 5.7928527471 14.3874417312 7.8994965542

O 3.8842187351 17.7029503806 7.8958439813

O 17.2779219999 1.1068123307 7.8341966059

O 15.3639511783 4.4246545459 7.8926554170

O 13.4509074741 7.7448273257 7.8908784678

O 11.5347349434 11.0646607313 7.8927603746

O 9.6158759926 14.3833161584 7.8913893782

O 7.6975405756 17.7023995832 7.8860346584

Ce -0.0000000000 -0.0000000000 3.9115480740

Ce -1.9162593770 3.3190586010 3.9115480740

Ce -3.8325187540 6.6381172030 3.9115480740

Ce -5.7487781310 9.9571758040 3.9115480740

Ce -7.6650375080 13.2762344060 3.9115480740

Ce -9.5812968850 16.5952930070 3.9115480740

Ce 3.8325187540 -0.0000000000 3.9115480740

Ce 1.9162593770 3.3190586010 3.9115480740

Ce -0.0000000000 6.6381172030 3.9115480740

Ce -1.9162593770 9.9571758040 3.9115480740

Ce -3.8325187540 13.2762344060 3.9115480740

Ce -5.7487781310 16.5952930070 3.9115480740

Ce 7.6650375080 0.0000000000 3.9115480740

Ce 5.7487781310 3.3190586010 3.9115480740

Ce 3.8325187540 6.6381172030 3.9115480740

Ce 1.9162593770 9.9571758040 3.9115480740

Ce 0.0000000000 13.2762344060 3.9115480740

Ce -1.9162593770 16.5952930070 3.9115480740

Ce 11.4975562620 0.0000000000 3.9115480740

Ce 9.5812968850 3.3190586010 3.9115480740

Ce 7.6650375080 6.6381172030 3.9115480740

Ce 5.7487781310 9.9571758040 3.9115480740

Ce 3.8325187540 13.2762344060 3.9115480740

Ce 1.9162593770 16.5952930070 3.9115480740

Ce 15.3300750160 -0.0000000000 3.9115480740

Ce 13.4138156390 3.3190586010 3.9115480740

Ce 11.4975562620 6.6381172030 3.9115480740

Ce 9.5812968850 9.9571758040 3.9115480740

Ce 7.6650375080 13.2762344060 3.9115480740

Ce 5.7487781310 16.5952930070 3.9115480740

Ce 0.0556616945 0.0074299676 13.4507176780

Ce -1.8547879888 3.3110328249 13.4561404811

Ce -3.7815637915 6.6294720111 13.4529397649

Ce -5.6724961636 9.9645620431 13.4398849190

Ce -7.5786991619 13.3012608508 13.4572085423

Ce -9.5221482908 16.6224138357 13.4730295503

Ce 3.8779288102 -0.0068335852 13.4712565689

Ce 1.9639965191 3.2525622995 13.4844323046

Ce 0.0238422613 6.6172963115 13.5130943624

Ce -1.9522571281 9.9447662764 13.4644035029

Ce -3.8040811788 13.3220256899 13.4901864045

Ce -5.7471930821 16.6309604247 13.4612863212

Ce 7.7152734059 0.0235489775 13.4606360876

Ce 5.8335256201 3.2432051932 13.4878076492

Ce 3.9631043678 6.4862086766 13.4934951464

Ce 1.8834318402 9.9656877951 13.5012220238

Ce -0.0027220015 13.1520698755 13.4428075806

Ce -2.0179061718 16.7053002437 13.4462089862

Ce 11.5705390823 -0.0041039675 13.4840194671

Ce 9.6557277788 3.3128923196 13.4713419324

Ce 7.8098114337 6.6276753542 13.5184536156

Ce 5.9146944174 10.0333695591 13.4905763382

Ce 3.8278707398 13.3412913381 13.5124715779

Ce 2.0771130406 16.6946882723 13.4363204417

Ce 15.4060798502 0.0556599216 13.4611311113

Ce 13.4669131123 3.3139330406 13.4551302388

Ce 11.5911771418 6.6413926770 13.4749907312

Ce 9.7465290590 10.0058831784 13.5107678920

Ce 7.7938895244 13.3419649222 13.6589084375

Ce 5.8118393002 16.6598658734 13.5464937903

Ce 1.9637897255 1.1038094588 10.2858608797

Ce 0.0537737907 4.4264867838 10.3073644496

Ce -1.8633700099 7.7425110417 10.2932696404

Ce -3.7809466308 11.0695523754 10.2810088710

Ce -5.7105499915 14.4008240278 10.2886888787

Ce -7.6275228715 17.7018156815 10.2728077977

Ce 5.7839083842 1.1068029934 10.2719078292

Ce 3.8883873310 4.4256679622 10.2927178756

Ce 1.9732633072 7.7467445421 10.3415385138

Ce 0.0381377401 11.0460930651 10.2599199979

Ce -1.8750836354 14.3996172080 10.3010355306

Ce -3.8120984998 17.7146222802 10.2417741834

Ce 9.6103500676 1.1014973613 10.2946433920

Ce 7.7156424536 4.4320397917 10.3112581314

Ce 5.8053175799 7.7625100505 10.3271164971

Ce 3.8613941201 11.0534094875 10.3415418538

Ce 1.9342306849 14.3828219259 10.3062245018

Ce 0.0361525811 17.6947516283 10.2946223622

Ce 13.4634651760 1.0951740629 10.2810999002

Ce 11.5494406791 4.4304072809 10.3019299169

Ce 9.6405199848 7.7636357148 10.3190963925

Ce 7.7208567133 11.0931866109 10.3280226017

Ce 5.7955207283 14.3816548192 10.3715016193

Ce 3.8963177853 17.7045017095 10.2693795346

Ce 17.3006095885 1.1081809503 10.2727536770

Ce 15.3860611408 4.4230662190 10.2833603383

Ce 13.4809481226 7.7483124275 10.2833002529

Ce 11.5594263825 11.0694444873 10.2866304084

Ce 9.6184261146 14.3786106822 10.3187337777

Ce 7.7133382780 17.6973205224 10.3004130715

Ce 0.0202338894 2.2167782994 7.0651131094

Ce -1.8955337238 5.5311216649 7.0701489292

Ce -3.8148864807 8.8517931427 7.0620354300

Ce -5.7375671684 12.1713913898 7.0680750986

Ce -7.6567989802 15.4862859675 7.0609204743

Ce -9.5704081275 18.8031680419 7.0563276831

Ce 3.8445895170 2.2166836854 7.0732986170

Ce 1.9343332793 5.5403825507 7.0906348963

Ce 0.0161169302 8.8456186519 7.0724440656

Ce -1.9100510922 12.1727554198 7.0688781639

Ce -3.8242468170 15.4871276338 7.0596287472

Ce -5.7477154315 18.8102031328 7.0396954731

Ce 7.6842985631 2.2165052234 7.0767142446

Ce 5.7867740514 5.5398657462 7.0897853507

Ce 3.8534846450 8.8560010852 7.0949471142

Ce 1.9333981147 12.1701185395 7.0808150045

Ce 0.0124485937 15.4882653958 7.0651187779

Ce -1.9032668079 18.8100482765 7.0623153736

Ce 11.5181078846 2.2137795661 7.0764199135

Ce 9.6134189689 5.5361296263 7.0874762781

Ce 7.6971226363 8.8564284045 7.0970391410

Ce 5.7690683895 12.1712171776 7.1073660679

Ce 3.8625462131 15.4782506095 7.0911985737

Ce 1.9375973683 18.8033664156 7.0740590388

Ce 15.3545539384 2.2095288219 7.0544299003

Ce 13.4392126571 5.5320534778 7.0689937984

Ce 11.5188677924 8.8514401871 7.0774389487

Ce 9.5988800360 12.1740441966 7.0817142088

Ce 7.6842181121 15.4812089658 7.0843439308

Ce 5.7783508202 18.8024069909 7.0545380851

Au -0.6634278689 6.3375114118 17.1391126919

Au 6.6728424875 7.3600361094 18.8451224859

Au 0.0732652211 15.4630242976 15.5240452510

Au 0.5348585591 11.3684480390 20.9755575446

Au -0.2597753370 10.6908907617 18.1995433328

Au 5.0160971301 9.6216517102 18.5133069250

Au 3.7403142661 8.7317327946 15.9425441215

Au 2.4374676983 6.0040580827 21.0321959561

Au 2.2605655071 6.1783919579 16.5203245053

Au 5.2662154931 6.1244941621 16.5201546854

Au 3.8477723160 12.2254181649 18.7454287712

Au 4.0365849806 8.7925240347 21.0420313942

Au 6.7440822859 8.8897348369 16.4362460364

Au 5.1181829678 11.3836805494 16.4196119278

Au 1.2184421405 7.2047865398 18.9528330873

Au 2.2278420590 10.0311080411 19.3238998560

Au 2.1071340242 11.1330521153 16.7082091171

Au 0.7985008298 8.6156063423 16.5696342396

Au 3.9580586079 6.9577185003 18.7339470773

Au 1.0550571457 8.6089736272 21.3830789674

C -2.5447543560 5.7043691380 17.4780327546

O -3.6300892920 5.8242055207 17.8376227561

C 8.2592270162 7.1121552816 20.0087927421

O 9.0089484582 7.2998903184 20.8604658348

C -1.4443716305 16.7091201068 18.3333237918

O -0.8880895862 16.2947739257 19.2771230474

C -0.3660697557 8.0450760032 22.6925107159

O -1.4131710293 7.6709456858 22.9919527672

C 5.1645267886 8.4753765922 22.6863565617

O 6.0991177391 8.3349593771 23.3429274657

C -0.6932852115 12.4051043179 22.1574304578

O -1.5612133910 12.5421451071 22.8993451371

C 2.6384762454 4.7238286802 22.5700831804

O 2.2722662213 4.0553932144 23.4315080690

C -2.2723843109 10.5406548743 18.1590042314

O -3.2648728048 9.9606489499 18.1054768388

(5) Au moves out of the oxygen defect (Configuration vi)

O 0.0073648389 -0.0002917765 6.2283911249

O -1.9104356654 3.3228534641 6.2296105336

O -3.8267902604 6.6376059668 6.2319996697

O -5.7433585740 9.9571276022 6.2321788375

O -7.6593097571 13.2745377297 6.2317801889

O -9.5792833146 16.5932384267 6.2288696273

O 3.8253205549 0.0068215724 6.2283334518

O 1.9159361973 3.3229010852 6.2787149635

O -0.0023041430 6.6385379078 6.2791983251

O -1.9218765339 9.9583851925 6.2340410955

O -3.8372257837 13.2765617782 6.2768676862

O -5.7544454337 16.5921351892 6.2266944847

O 7.6560162917 0.0075183095 6.2334396854

O 5.7840123127 3.3237608719 6.2845142587

O 3.8735708394 6.6398086345 6.2898602221

O 1.9099276579 9.9566971919 6.2729765072

O -0.0149148617 13.2768840814 6.2393300543

O -1.8826661462 16.5931937887 6.2319637973

O 11.5258343142 0.0011333399 6.2893926481

O 9.6142775631 3.3177119321 6.2854433851

O 7.6963090149 6.6375475954 6.2873446004

O 5.7762406703 9.9575904256 6.2928913181

O 3.8590113376 13.2770987310 6.2918470797

O 1.9442767496 16.5961703052 6.2903515203

O 15.3432278981 -0.0035674792 6.2333258461

O 13.4296806349 3.3176671078 6.2338304974

O 11.5180214985 6.6375331597 6.2834711636

O 9.6009319802 9.9570607187 6.2858953250

O 7.6815999928 13.2737668880 6.2854098222

O 5.7637017107 16.5932511096 6.2373526626

O 0.0522978140 2.2196315046 9.4838407632

O -1.8613336505 5.5338979568 9.4909256627

O -3.7791345887 8.8532824315 9.4890040657

O -5.6875184339 12.1751752455 9.4926028623

O -7.6126532373 15.4924636952 9.4867647879

O -9.5306325794 18.8096999839 9.4890972653

O 3.8675875360 2.2178273107 9.4892136774

O 1.9513500821 5.5310879916 9.4951791001

O 0.0321028477 8.8394837014 9.4839384619

O -1.8918041046 12.1737777322 9.4822327123

O -3.7991310920 15.4909027889 9.4736762032

O -5.7132412280 18.8078142974 9.4214549586

O 7.6908847941 2.2158510785 9.4931257144

O 5.8353388084 5.5296951506 9.4923125466

O 3.8510818313 8.8492932580 9.4959092725

O 1.9355861194 12.1754075558 9.4906066923

O 0.0197181436 15.4909451581 9.5006796041

O -1.8962323005 18.8093014771 9.4866328051

O 11.5034881610 2.2047999438 9.4955563120

O 9.6577292603 5.5347744175 9.5030355196

O 7.7516255165 8.8473749048 9.5000899937

O 5.7481843042 12.1751207891 9.4980718714

O 3.8350091807 15.4809434549 9.4922750252

O 1.9180040903 18.8118622994 9.4853101935

O 15.3889301984 2.2125478898 9.4887398578

O 13.4751118933 5.5320076324 9.4910805094

O 11.5609892833 8.8466220787 9.4932358895

O 9.6466903412 12.1676144230 9.4944889353

O 7.7247637998 15.4924057012 9.4965801383

O 5.8128672561 18.8168782705 9.4870711268

O 1.9162593770 1.1063528670 3.1292384590

O 0.0000000000 4.4254114690 3.1292384590

O -1.9162593770 7.7444700700 3.1292384590

O -3.8325187540 11.0635286720 3.1292384590

O -5.7487781310 14.3825872730 3.1292384590

O -7.6650375080 17.7016458750 3.1292384590

O 5.7487781310 1.1063528670 3.1292384590

O 3.8325187540 4.4254114690 3.1292384590

O 1.9162593770 7.7444700700 3.1292384590

O 0.0000000000 11.0635286720 3.1292384590

O -1.9162593770 14.3825872730 3.1292384590

O -3.8325187540 17.7016458750 3.1292384590

O 9.5812968850 1.1063528670 3.1292384590

O 7.6650375080 4.4254114690 3.1292384590

O 5.7487781310 7.7444700700 3.1292384590

O 3.8325187540 11.0635286720 3.1292384590

O 1.9162593770 14.3825872730 3.1292384590

O 0.0000000000 17.7016458750 3.1292384590

O 13.4138156390 1.1063528670 3.1292384590

O 11.4975562620 4.4254114690 3.1292384590

O 9.5812968850 7.7444700700 3.1292384590

O 7.6650375080 11.0635286720 3.1292384590

O 5.7487781310 14.3825872730 3.1292384590

O 3.8325187540 17.7016458750 3.1292384590

O 17.2463343930 1.1063528670 3.1292384590

O 15.3300750160 4.4254114690 3.1292384590

O 13.4138156390 7.7444700700 3.1292384590

O 11.4975562620 11.0635286720 3.1292384590

O 9.5812968850 14.3825872730 3.1292384590

O 7.6650375080 17.7016458750 3.1292384590

O 2.0034775456 1.1043642640 12.6142122248

O 0.0254860361 4.4257653968 12.6713808612

O -1.8974355944 7.7397719479 12.6113112377

O -3.8127529809 11.0680961546 12.6092287724

O -5.6914604173 14.4342008889 12.6163370614

O -7.6011691897 17.7609340671 12.6061179764

O 5.8306967029 1.1108815739 12.6778869950

O 3.9537730946 4.3634854097 12.6850777455

O 2.0036536125 7.7473352909 12.7783011607

O -0.0077649355 11.0722615972 12.6841471844

O -1.7422312876 14.5154613008 12.7716746405

O -3.7972630812 17.7679761969 12.6105443768

O 9.6457817747 1.1068623387 12.6800105423

O 7.7234418094 4.4293171236 12.6881199998

O 5.8186132709 7.7386300866 12.7782642852

O 3.8464587187 11.0010249912 12.6981719012

O 1.7333409722 14.5185098331 12.6954088620

O 0.0030787574 17.5639846961 12.7598681820

O 13.4605993103 1.1143821004 12.6108642515

O 11.5544006042 4.4243812402 12.6799063534

O 9.6402764877 7.7456207257 12.6852887450

O 7.8069825966 11.0579652424 12.6881625562

O 5.7135867939 14.3781811047 12.6835786509

O 3.8470761083 17.7721805445 12.6809996101

O 17.3153371604 1.1730595454 12.5988595125

O 15.3719256437 4.4234575283 12.6123017572

O 13.5229447394 7.7460742411 12.6175647689

O 11.6233253420 11.0656268534 12.6147164354

O 9.7079875125 14.3882683275 12.6150373254

O 7.7530234971 17.7782208535 12.6075864362

O -0.0000000000 2.2127057340 4.6938576890

O -1.9162593770 5.5317643360 4.6938576890

O -3.8325187540 8.8508229370 4.6938576890

O -5.7487781310 12.1698815390 4.6938576890

O -7.6650375080 15.4889401400 4.6938576890

O -9.5812968850 18.8079987420 4.6938576890

O 3.8325187540 2.2127057340 4.6938576890

O 1.9162593770 5.5317643360 4.6938576890

O 0.0000000000 8.8508229370 4.6938576890

O -1.9162593770 12.1698815390 4.6938576890

O -3.8325187540 15.4889401400 4.6938576890

O -5.7487781310 18.8079987420 4.6938576890

O 7.6650375080 2.2127057340 4.6938576890

O 5.7487781310 5.5317643360 4.6938576890

O 3.8325187540 8.8508229370 4.6938576890

O 1.9162593770 12.1698815390 4.6938576890

O -0.0000000000 15.4889401400 4.6938576890

O -1.9162593770 18.8079987420 4.6938576890

O 11.4975562620 2.2127057340 4.6938576890

O 9.5812968850 5.5317643360 4.6938576890

O 7.6650375080 8.8508229370 4.6938576890

O 5.7487781310 12.1698815390 4.6938576890

O 3.8325187540 15.4889401400 4.6938576890

O 1.9162593770 18.8079987420 4.6938576890

O 15.3300750160 2.2127057340 4.6938576890

O 13.4138156390 5.5317643360 4.6938576890

O 11.4975562620 8.8508229370 4.6938576890

O 9.5812968850 12.1698815390 4.6938576890

O 7.6650375080 15.4889401400 4.6938576890

O 5.7487781310 18.8079987420 4.6938576890

O 0.0474156196 0.0567051080 11.0959430057

O -1.8419716490 3.3283950141 11.0953777915

O -3.7566618314 6.6323894163 11.0941117998

O -5.6685670458 9.9522593879 11.0945639579

O -7.5805553292 13.2728623977 11.0970974163

O -9.5051193009 16.5980835963 11.0976929070

O 3.9049917422 0.0062291059 11.0945887671

O 1.9809205079 3.3285640135 11.1593243270

O 0.0704846724 6.6413938667 11.1732237694

O -1.8579265966 9.9578531580 11.1005921717

O -3.7230705191 13.3371683160 11.1782944930

O -5.6868445690 16.6064741885 11.0941169094

O 7.7103411045 0.0066876173 11.0987015275

O 5.8054952401 3.3152795849 11.1049223639

O 3.8874045508 6.6329624778 11.0849855101

O 1.8910026517 9.9536900649 11.0897418388

O 0.0021412054 13.2199269588 11.0997386125

O -1.9400075132 16.6013950210 11.0944406278

O 11.5023618207 -0.0660877703 11.1757167508

O 9.6205651376 3.3184443526 11.1654865614

O 7.7081165047 6.6415957886 11.1737961169

O 5.7944768176 9.9577912708 11.0146867139

O 3.7797361446 13.2817574538 11.1853574142

O 1.9862976869 16.6582261198 11.0983988338

O 15.3608754009 0.0043242622 11.0938360822

O 13.4379741222 3.3186054266 11.0976502840

O 11.5826038244 6.6370201106 11.1639962594

O 9.6787254034 9.9569967246 11.1591081788

O 7.6872540050 13.2787608943 11.0901985218

O 5.7718348113 16.6006911353 11.0878540734

O 0.0566289397 2.2116837029 14.2837245175

O -1.8684529410 5.5350475460 14.2894999218

O -3.7815937921 8.8528271192 14.2874041574

O -5.6464000394 12.2377536967 14.2847827709

O -7.5681296847 15.5563184181 14.2864787124

O -9.4879675997 18.8757208383 14.2887354680

O 3.8752235939 2.2050488097 14.2928659512

O 1.8632199926 5.3819019604 14.3781927582

O -0.1370324051 8.8403058963 14.2992378926

O -1.9646518768 12.1707819789 14.2825053134

O -3.8399301651 15.5563169205 14.2835223602

O -5.7082382506 18.8111159626 14.2891468674

O 7.7610759766 2.2086450345 14.2940360342

O 5.9432350721 5.3876648011 14.5478614533

O 1.8622889061 12.3069022816 14.3854129183

O -1.9007709939 18.8221324421 14.2912456706

O 11.5837480831 2.2071330463 14.2882611650

O 9.6705151536 5.5318540622 14.2919663433

O 7.9685493754 8.7736005052 14.5498196595

O 5.6201730696 12.3867896863 14.5477513457

O 3.7338941519 15.4826364249 14.3642652520

O 1.9579487287 18.8821518416 14.2886176496

O 15.3952200425 2.2235069676 14.2817168042

O 13.4848573409 5.5335237235 14.2831118802

O 11.6510443021 8.8501965131 14.2839352920

O 9.8692680547 12.0975153275 14.3750577833

O 7.9160342051 15.6375619477 14.3806265905

O 5.7891832918 19.0265326425 14.2948785962

O 1.9355235121 1.1046586250 7.8868965386

O 0.0173303692 4.4256721390 7.8935538501

O -1.9017302955 7.7442074995 7.8852600842

O -3.8157100526 11.0599516768 7.8859413909

O -5.7325599255 14.3833292597 7.8844617392

O -7.6514634343 17.7002340412 7.8269924241

O 5.7520288592 1.1083448570 7.8911531182

O 3.8434656807 4.4267303802 7.8986825175

O 1.9227381487 7.7439052739 7.8969502110

O 0.0010907231 11.0633416482 7.8923276546

O -1.9107760227 14.3850177568 7.8957168666

O -3.8240116468 17.7000208732 7.8246052406

O 9.5745327980 1.1025973245 7.8935071202

O 7.7282067674 4.4251061741 7.8999342891

O 5.8034297932 7.7471134274 7.9094091907

O 3.8217932446 11.0613525129 7.8964432719

O 1.9037435822 14.3810987263 7.8952615295

O -0.0099434576 17.6989603744 7.8977964835

O 13.4609996328 1.1098708238 7.8946639994

O 11.5492821835 4.4226119743 7.8987161337

O 9.6317600354 7.7438404782 7.8996533107

O 7.7096078983 11.0666686842 7.9038605037

O 5.7921232772 14.3877861141 7.8996823717

O 3.8806377181 17.7046486078 7.8978234912

O 17.2780613701 1.1074473220 7.8321704489

O 15.3638985624 4.4244142203 7.8873857466

O 13.4505247234 7.7450945728 7.8902553020

O 11.5349336724 11.0645301904 7.8924516746

O 9.6160969723 14.3834723410 7.8903136188

O 7.6973611106 17.7022410624 7.8861650633

Ce 0.0000000000 0.0000000000 3.9115480740

Ce -1.9162593770 3.3190586010 3.9115480740

Ce -3.8325187540 6.6381172030 3.9115480740

Ce -5.7487781310 9.9571758040 3.9115480740

Ce -7.6650375080 13.2762344060 3.9115480740

Ce -9.5812968850 16.5952930070 3.9115480740

Ce 3.8325187540 -0.0000000000 3.9115480740

Ce 1.9162593770 3.3190586010 3.9115480740

Ce 0.0000000000 6.6381172030 3.9115480740

Ce -1.9162593770 9.9571758040 3.9115480740

Ce -3.8325187540 13.2762344060 3.9115480740

Ce -5.7487781310 16.5952930070 3.9115480740

Ce 7.6650375080 -0.0000000000 3.9115480740

Ce 5.7487781310 3.3190586010 3.9115480740

Ce 3.8325187540 6.6381172030 3.9115480740

Ce 1.9162593770 9.9571758040 3.9115480740

Ce 0.0000000000 13.2762344060 3.9115480740

Ce -1.9162593770 16.5952930070 3.9115480740

Ce 11.4975562620 -0.0000000000 3.9115480740

Ce 9.5812968850 3.3190586010 3.9115480740

Ce 7.6650375080 6.6381172030 3.9115480740

Ce 5.7487781310 9.9571758040 3.9115480740

Ce 3.8325187540 13.2762344060 3.9115480740

Ce 1.9162593770 16.5952930070 3.9115480740

Ce 15.3300750160 0.0000000000 3.9115480740

Ce 13.4138156390 3.3190586010 3.9115480740

Ce 11.4975562620 6.6381172030 3.9115480740

Ce 9.5812968850 9.9571758040 3.9115480740

Ce 7.6650375080 13.2762344060 3.9115480740

Ce 5.7487781310 16.5952930070 3.9115480740

Ce 0.0529420380 0.0174990305 13.4498565227

Ce -1.8547812120 3.3148002416 13.4375131050

Ce -3.7896510409 6.6290600120 13.4247065283

Ce -5.6775126822 9.9647487768 13.4412599113

Ce -7.5942100437 13.3009933674 13.4608147026

Ce -9.5257742871 16.6217805852 13.4752405551

Ce 3.8771916401 -0.0093939564 13.4729832081

Ce 1.9605711066 3.2441485662 13.4818766693

Ce 0.0092638360 6.6242597652 13.4843172786

Ce -1.9620302930 9.9461148876 13.4460717753

Ce -3.8149456226 13.3211146423 13.4922433186

Ce -5.7508283163 16.6289594724 13.4600732799

Ce 7.7111454952 0.0251410393 13.4711582683

Ce 5.8312299123 3.2476103863 13.4895018622

Ce 3.8914968400 6.4979416473 13.4896105706

Ce 1.8407387178 10.0106179224 13.4896342560

Ce -0.0326923732 13.1833515577 13.4441862143

Ce -2.0296563163 16.7038862089 13.4459566908

Ce 11.5541972860 -0.0007180450 13.4938716357

Ce 9.6537777764 3.3153178996 13.4830944365

Ce 7.7995481823 6.6311047582 13.5173602975

Ce 5.8980718244 10.0389578922 13.4134417732

Ce 3.8129001592 13.3840599442 13.5264762516

Ce 2.0725515145 16.7110987695 13.4539639769

Ce 15.4102695374 0.0630557726 13.4611079442

Ce 13.4658561505 3.3141359478 13.4486250998

Ce 11.5853447257 6.6419493605 13.4754951588

Ce 9.7407167520 10.0153653287 13.5152718311

Ce 7.7335657155 13.3557830192 13.6011222527

Ce 5.8323953845 16.6550431883 13.5867061330

Ce 1.9636283064 1.1013808429 10.2837240073

Ce 0.0535678907 4.4230218986 10.3028714307

Ce -1.8678112325 7.7428633233 10.2792187551

Ce -3.7855352629 11.0700534104 10.2782784076

Ce -5.7115683653 14.4002015245 10.2895315138

Ce -7.6300747152 17.7029669431 10.2733286669

Ce 5.7838329352 1.1063651746 10.2745806019

Ce 3.8869690461 4.4241062230 10.2926569057

Ce 1.9665509454 7.7492206849 10.3284076092

Ce 0.0257752276 11.0525500950 10.2527062420

Ce -1.8821011736 14.3964307846 10.2948296728

Ce -3.8161120714 17.7095539749 10.2415392638

Ce 9.6062540373 1.0998737609 10.3057003052

Ce 7.7165897239 4.4296247162 10.3136626594

Ce 5.8023842809 7.7528066376 10.3255419195

Ce 3.8465711269 11.0633419554 10.3340315030

Ce 1.9282321853 14.3948652371 10.3113482895

Ce 0.0239087664 17.6907942253 10.3082042044

Ce 13.4570371058 1.0942240159 10.2892102207

Ce 11.5490693156 4.4287193097 10.3034661861

Ce 9.6406589402 7.7639405337 10.3223406712

Ce 7.7275699863 11.0933478299 10.3154596390

Ce 5.7879871350 14.3924980302 10.3626669228

Ce 3.8983993802 17.7047760288 10.2908432350

Ce 17.3015351675 1.1108588087 10.2707545392

Ce 15.3839052255 4.4209219533 10.2744190949

Ce 13.4782949947 7.7477012501 10.2801316213

Ce 11.5592858879 11.0693766048 10.2882767753

Ce 9.6218686344 14.3833550112 10.3173340020

Ce 7.7068254638 17.6963785388 10.3083489760

Ce 0.0200480889 2.2155559506 7.0638218454

Ce -1.8959177591 5.5300948871 7.0678337181

Ce -3.8164533504 8.8518569927 7.0596086203

Ce -5.7380272279 12.1719167243 7.0677552234

Ce -7.6562404989 15.4870744400 7.0605838814

Ce -9.5715549657 18.8025343209 7.0576466139

Ce 3.8452467083 2.2154474775 7.0736736478

Ce 1.9343129983 5.5383511891 7.0893472568

Ce 0.0140693800 8.8460438842 7.0726320090

Ce -1.9116250024 12.1732601266 7.0665531915

Ce -3.8255361885 15.4877119115 7.0598818703

Ce -5.7476573702 18.8106189973 7.0402562096

Ce 7.6848340362 2.2158218026 7.0784734453

Ce 5.7868162483 5.5382496776 7.0888330887

Ce 3.8534266935 8.8563267043 7.0952521844

Ce 1.9318418787 12.1725724056 7.0825390224

Ce 0.0121427792 15.4894885976 7.0658804683

Ce -1.9033054257 18.8101703587 7.0645221223

Ce 11.5168889096 2.2128261720 7.0787887909

Ce 9.6137653853 5.5354860016 7.0886451179

Ce 7.6977847490 8.8547192037 7.0970189447

Ce 5.7685335964 12.1726493347 7.1072578859

Ce 3.8609999690 15.4810268971 7.0920413891

Ce 1.9375474513 18.8027860841 7.0832847964

Ce 15.3540247552 2.2079866646 7.0545240512

Ce 13.4384020060 5.5311100203 7.0682881791

Ce 11.5182627917 8.8513899258 7.0777417241

Ce 9.6003079691 12.1753280114 7.0798447618

Ce 7.6849812751 15.4833793358 7.0853195002

Ce 5.7774085061 18.8015848695 7.0583554690

Au -0.6272559906 6.3770208405 17.1117421957

Au 6.6988519140 7.3905837736 18.8083332088

Au -0.0022455254 15.5224540331 15.5580727491

Au 0.4779160973 11.3877595428 20.8959356601

Au -0.2767701993 10.7290367293 18.1312213747

Au 4.9989348310 9.6271055438 18.4812253984

Au 3.7374115772 8.7527665696 15.9173609855

Au 2.4408173514 6.0265809834 21.0487964413

Au 2.2620301180 6.1839257465 16.5095132315

Au 5.2721752719 6.1336751237 16.5005386451

Au 3.8265838778 12.2408526930 18.7040015257

Au 3.9832983008 8.8651537182 21.0149687770

Au 6.7326958288 8.9078346559 16.3987529309

Au 5.0969784582 11.3861357315 16.3799426442

Au 1.2617722866 7.1975732588 18.9353657031

Au 2.1986294181 10.0602619702 19.2639195360

Au 2.0896975308 11.1978412597 16.6458509582

Au 0.8540822512 8.6498446221 16.5546987479

Au 3.9932770545 6.9357825728 18.7411707299

Au 1.0174677050 8.6268127367 21.3272213121

C -2.5147824359 5.7706357876 17.4437094938

O -3.5866228301 5.8974572974 17.8378463285

C 8.2759669061 7.1502506326 19.9850059711

O 9.0075069559 7.2996014102 20.8595544065

C -0.3901088438 8.0767786770 22.6524754829

O -1.4130613178 7.6708439417 22.9904557372

C 5.1028105945 8.5091235635 22.6575986969

O 6.0106824572 8.3355914908 23.3422128456

C -0.7246448602 12.3996683891 22.1227159770

O -1.5611263339 12.5418499502 22.8988152197

C 2.6409505108 4.7609680499 22.6010526071

O 2.2720470221 4.0556094295 23.4309512359

C -2.2869555648 10.5630206862 18.0923046937

O -3.2666317422 9.9599885942 18.1048309624

(6) Au+-CO at the oxygen site (Cofig. vii)

O 0.0207414425 -0.0082903908 6.2064059931

O -1.9069972970 3.3099838993 6.2016022297

O -3.8333250395 6.6190042351 6.1988463668

O -5.7601520097 9.9313513691 6.1916070637

O -7.6892713736 13.2419355762 6.1816689203

O -9.6161505125 16.5574920651 6.1764646690

O 3.8369623906 0.0104280582 6.2203547877

O 1.9182293104 3.3209823389 6.2663698983

O -0.0088390367 6.6315409915 6.2608564271

O -1.9363870814 9.9444800476 6.2102709784

O -3.8621147230 13.2538852652 6.2010803079

O -5.7938048136 16.5706469821 6.1867829935

O 7.6564185898 0.0194705287 6.2322499036

O 5.7833162688 3.3349295325 6.2818482466

O 3.8675544922 6.6433981515 6.2856854591

O 1.9247854439 9.9532311260 6.2384790460

O -0.0401033044 13.2675045093 6.2217435600

O -1.9687129362 16.5777787187 6.2046519720

O 11.5399667281 0.0292635493 6.2545424388

O 9.6159293891 3.3432426854 6.2479114396

O 7.6890073296 6.6528926750 6.2970967018

O 5.7603703659 9.9668099853 6.2968639542

O 3.8330561242 13.2796315664 6.2906400855

O 1.9101860131 16.5913572106 6.2819889673

O 15.3579862022 0.0344134712 6.2685499928

O 13.4339505945 3.3518933862 6.2620191363

O 11.5109112238 6.6644658559 6.3091418849

O 9.5837845274 9.9780610138 6.3050890375

O 7.6555957813 13.2877253663 6.2973420103

O 5.7288512974 16.6020368656 6.2934251657

O 0.0452959405 2.2090214683 9.4642971841

O -1.8784866488 5.5175064241 9.4638732148

O -3.8077529597 8.8303610226 9.4561074730

O -5.7269210314 12.1485172031 9.4493774273

O -7.6560026108 15.4574510885 9.4394026203

O -9.5846897531 18.7714770572 9.4390892206

O 3.8612137180 2.2193791979 9.4842154899

O 1.9359232844 5.5275221236 9.4836781544

O 0.0109097394 8.8372759383 9.4733495377

O -1.9189314021 12.1580083282 9.4662289387

O -3.8490472885 15.4628391065 9.4458287868

O -5.7704268913 18.7810360666 9.3861440418

O 7.6832080121 2.2278227427 9.4944401955

O 5.8185113947 5.5402900666 9.4934435714

O 3.8372840525 8.8480849816 9.4884561733

O 1.9018892928 12.1657218781 9.4796473441

O -0.0308084731 15.4813549188 9.4783778888

O -1.9496701237 18.7964464212 9.4504750428

O 11.4978994766 2.2343443916 9.5052310653

O 9.6353408933 5.5592903878 9.5156658684

O 7.7193182366 8.8649318149 9.5131173807

O 5.7207015032 12.1734514204 9.4991038293

O 3.8233096054 15.4221808937 9.4928625179

O 1.8637363785 18.8036839463 9.4184475483

O 15.3852201613 2.2493411041 9.5260295989

O 13.4563944608 5.5647280967 9.5250032238

O 11.5336624817 8.8718433775 9.5214166301

O 9.6079081999 12.1845034759 9.5147344351

O 7.6848124586 15.5010767875 9.5109501205

O 5.7552511729 18.8268816433 9.4954340884

O 1.9381239261 1.1061235039 3.1145407020

O 0.0120510955 4.4194920089 3.1088306279

O -1.9140217351 7.7328605129 3.1031205537

O -3.8400945656 11.0462290179 3.0974104796

O -5.7661673962 14.3595975219 3.0917004054

O -7.6922402267 17.6729660269 3.0859903313

O 5.7705964852 1.1174735758 3.1295493382

O 3.8445236546 4.4308420807 3.1238392641

O 1.9184508241 7.7442105847 3.1181291899

O -0.0076220065 11.0575790897 3.1124191158

O -1.9336948370 14.3709475937 3.1067090417

O -3.8597676676 17.6843160987 3.1009989675

O 9.6030690443 1.1288236476 3.1445579744

O 7.6769962137 4.4421921526 3.1388479003

O 5.7509233832 7.7555606566 3.1331378262

O 3.8248505526 11.0689291616 3.1274277520

O 1.8987777221 14.3822976656 3.1217176779

O -0.0272951085 17.6956661706 3.1160076037

O 13.4355416034 1.1401737194 3.1595666107

O 11.5094687729 4.4535422244 3.1538565365

O 9.5833959423 7.7669107284 3.1481464624

O 7.6573231118 11.0802792334 3.1424363882

O 5.7312502812 14.3936477374 3.1367263141

O 3.8051774507 17.7070162424 3.1310162399

O 17.2680141626 1.1515237912 3.1745752469

O 15.3419413320 4.4648922962 3.1688651727

O 13.4158685014 7.7782608002 3.1631550986

O 11.4897956709 11.0916293052 3.1574450244

O 9.5637228403 14.4049978092 3.1517349503

O 7.6376500098 17.7183663142 3.1460248761

O 1.9891178354 1.0992809829 12.6012985551

O 0.0001129826 4.4102888475 12.6498343563

O -1.9329061419 7.7209148121 12.5840499800

O -3.8573554326 11.0376284068 12.5779349431

O -5.7826641354 14.3591901212 12.5695247903

O -7.6377193379 17.6729216903 12.5665089009

O 5.8101513278 1.1214116601 12.6736078946

O 3.9018594294 4.4144439131 12.6809396116

O 1.9314954037 7.6820870933 12.7516636134

O -0.0478097711 11.0403805952 12.6657254269

O -1.8407209240 14.4410757903 12.7584944707

O -3.9321442807 17.7487345291 12.5794751311

O 9.6321304199 1.1391832279 12.6206517518

O 7.7018334568 4.4411373016 12.6936701086

O 5.7529096168 7.8298542906 12.7875188554

O 3.9073596798 10.9733715415 12.7707109614

O 1.9152333170 14.3793954299 12.7605759916

O -0.0407942398 17.6270862722 12.6818919908

O 13.4505698459 1.1496958125 12.6329968389

O 11.5271777596 4.4454032722 12.6994801465

O 9.5996601212 7.7646665083 12.7128859050

O 7.7099729525 11.0057515306 12.6990462453

O 5.6504499638 14.3861698436 12.7048657884

O 3.8139550651 17.7061746130 12.6864047248

O 17.2695715176 1.1612398886 12.6478066778

O 15.3461530360 4.4565994792 12.6511409425

O 13.4816673388 7.7713527206 12.6497964135

O 11.5736008683 11.0875686732 12.7006080404

O 9.6487246775 14.4000786725 12.6298304341

O 7.6888898912 17.7794397243 12.6276661224

O 0.0124840788 2.2059323511 4.6722414690

O -1.9135887518 5.5193008560 4.6665313949

O -3.8396615823 8.8326693600 4.6608213207

O -5.7657344129 12.1460378650 4.6551112466

O -7.6918072434 15.4594063690 4.6494011724

O -9.6178800740 18.7727748740 4.6436910983

O 3.8449566379 2.2172824229 4.6872501052

O 1.9188838073 5.5306509279 4.6815400311

O -0.0071890232 8.8440194319 4.6758299569

O -1.9332618538 12.1573879369 4.6701198828

O -3.8593346843 15.4707564409 4.6644098087

O -5.7854075149 18.7841249459 4.6586997345

O 7.6774291970 2.2286324947 4.7022587414

O 5.7513563665 5.5420009997 4.6965486673

O 3.8252835359 8.8553695037 4.6908385932

O 1.8992107054 12.1687380087 4.6851285190

O -0.0268621252 15.4821065127 4.6794184449

O -1.9529349557 18.7954750177 4.6737083707

O 11.5099017562 2.2399825666 4.7172673777

O 9.5838289256 5.5533510715 4.7115573035

O 7.6577560951 8.8667195755 4.7058472294

O 5.7316832645 12.1800880805 4.7001371552

O 3.8056104339 15.4934565845 4.6944270811

O 1.8795376034 18.8068250895 4.6887170069

O 15.3423743153 2.2513326384 4.7322760139

O 13.4163014847 5.5647011434 4.7265659397

O 11.4902286542 8.8780696474 4.7208558656

O 9.5641558236 12.1914381524 4.7151457914

O 7.6380829931 15.5048066564 4.7094357173

O 5.7120101625 18.8181751614 4.7037256431

O 0.0417639643 0.0421605346 11.0743904937

O -1.8578756289 3.3062372382 11.0712717106

O -3.7856957826 6.6101074207 11.0599600739

O -5.7103902587 9.9266860523 11.0576462725

O -7.6339938066 13.2303881985 11.0499605843

O -9.5583162014 16.5584992599 11.0463929196

O 3.8988193702 0.0014672250 11.0901416878

O 1.9656113944 3.3235345386 11.1457402665

O 0.0398071189 6.6291237943 11.1459106339

O -1.8920040987 9.9423007556 11.0755768321

O -3.7994287660 13.2675445995 11.1390354392

O -5.7404631819 16.5683942091 11.0543328078

O 7.7043376155 0.0172820330 11.1000776028

O 5.7871012471 3.3248119730 11.1091102253

O 3.8707122213 6.6407391643 11.0795591180

O 1.9289319524 9.9513562073 11.0822510783

O -0.0007920861 13.2613897781 11.0777548054

O -1.9935469281 16.5883541944 11.0515536263

O 11.5305841243 0.0111327503 11.1236071367

O 9.6034286402 3.3424065345 11.1200268862

O 7.6740902123 6.6551447227 11.1907258579

O 5.7648256624 9.9645215372 11.0977319847

O 3.7768932028 13.2062551769 11.1768637105

O 1.8994637922 16.5928573522 11.0106478000

O 15.3461809807 0.0262552605 11.1296217356

O 13.4213499886 3.3489916843 11.1264849246

O 11.5067105408 6.6632321797 11.1901976465

O 9.6443008813 9.9811395517 11.1891795585

O 7.6430140720 13.2827793395 11.1119299260

O 5.7220266371 16.6036787300 11.0311811568

O 0.0277189446 2.1976184676 14.2588964471

O -1.9093082020 5.5107887289 14.2629478220

O -3.8298015569 8.8260156874 14.2564915561

O -5.7082531499 12.1986158753 14.2523462180

O -7.6707587795 15.4593296128 14.2449107065

O -9.5583849535 18.8271088252 14.2344856693

O 3.8532767656 2.2073631072 14.2827416950

O 1.8276972933 5.3737730123 14.3782575824

O -0.2262774496 8.7640659160 14.3614908493

O -2.0732091010 12.0765333647 14.3503617242

O -3.9622364713 15.4617822576 14.2674826253

O -5.7875035167 18.7824042788 14.2562978819

O 7.7380167725 2.2236918261 14.2968981782

O 5.9158961915 5.4002636611 14.5459402574

O 2.0059236202 12.2348946086 14.5497679917

O -1.9702307628 18.9363956519 14.2780658189

O 11.5544362592 2.2355817272 14.3076825475

O 9.6313276570 5.5502676876 14.3083960926

O 7.7930229946 8.8571784342 14.4735403450

O 5.7033761567 12.1716448344 14.3991004819

O 3.7726773994 15.3360513343 14.7394144335

O 1.8849617656 19.0116812821 14.3665036699

O 15.3712671143 2.2548192071 14.3199665052

O 13.4484637608 5.5615916023 14.3148677219

O 11.5991508126 8.8698879548 14.3145525455

O 9.7266846473 12.1119382916 14.3862551210

O 7.8808285919 15.5750478805 14.3953540332

O 5.7487556710 18.9735924933 14.3128795630

O 1.9388162177 1.1017379160 7.8727367957

O 0.0110925688 4.4173425114 7.8733519066

O -1.9157203367 7.7318716034 7.8596522929

O -3.8406287768 11.0429243876 7.8513952261

O -5.7689293651 14.3548178516 7.7903083282

O -7.6976425934 17.6680559319 7.7845239796

O 5.7555455657 1.1175307776 7.8896876464

O 3.8370672556 4.4300023410 7.8934469728

O 1.9066713035 7.7429246675 7.8852900443

O -0.0223788295 11.0538305616 7.8738898262

O -1.9490363555 14.3678888153 7.8709111794

O -3.8729873636 17.6808927163 7.7961923826

O 9.5808396424 1.1244218134 7.8418207739

O 7.7228915041 4.4376393992 7.9064280686

O 5.7902685815 7.7542754766 7.9128200260

O 3.7960101720 11.0641799603 7.8964953370

O 1.8658619884 14.3768910256 7.8873941443

O -0.0597423801 17.6893364740 7.8189573971

O 13.4601522123 1.1449540360 7.9136538340

O 11.5402022609 4.4479444530 7.9192499497

O 9.6141711084 7.7645200210 7.9186936028

O 7.6845598644 11.0797752846 7.9167467288

O 5.7607713548 14.3904633195 7.9063988683

O 3.8346615319 17.7026969102 7.9019458895

O 17.2809569569 1.1503432904 7.8784615837

O 15.3560288203 4.4618537598 7.9269768752

O 13.4333615139 7.7765857801 7.9244757930

O 11.5082378841 11.0903022665 7.9232538428

O 9.5812535276 14.4018782366 7.9121189142

O 7.6540203285 17.7171740033 7.9060426692

Ce 0.0221041381 -0.0063313604 3.8887418045

Ce -1.9039686924 3.3070371436 3.8830317304

Ce -3.8300415230 6.6204056486 3.8773216562

Ce -5.7561143535 9.9337741526 3.8716115821

Ce -7.6821871841 13.2471426576 3.8659015079

Ce -9.6082600147 16.5605111615 3.8601914338

Ce 3.8545766973 0.0050187114 3.9037504407

Ce 1.9285038667 3.3183872154 3.8980403666

Ce 0.0024310361 6.6317557204 3.8923302924

Ce -1.9236417944 9.9451242244 3.8866202183

Ce -3.8497146250 13.2584927294 3.8809101441

Ce -5.7757874555 16.5718612334 3.8752000700

Ce 7.6870492564 0.0163687832 3.9187590769

Ce 5.7609764258 3.3297372872 3.9130490028

Ce 3.8349035953 6.6431057922 3.9073389286

Ce 1.9088307647 9.9564742962 3.9016288545

Ce -0.0172420658 13.2698428012 3.8959187803

Ce -1.9433148964 16.5832113052 3.8902087062

Ce 11.5195218155 0.0277188551 3.9337677131

Ce 9.5934489850 3.3410873591 3.9280576390

Ce 7.6673761544 6.6544558641 3.9223475648

Ce 5.7413033238 9.9678243681 3.9166374907

Ce 3.8152304933 13.2811928730 3.9109274165

Ce 1.8891576627 16.5945613770 3.9052173424

Ce 15.3519943746 0.0390689269 3.9487763493

Ce 13.4259215441 3.3524374309 3.9430662752

Ce 11.4998487135 6.6658059359 3.9373562010

Ce 9.5737758830 9.9791744399 3.9316461269

Ce 7.6477030524 13.2925429449 3.9259360527

Ce 5.7216302219 16.6059114489 3.9202259786

Ce 0.0371112737 0.0023091414 13.4262053027

Ce -1.8867397096 3.2792326915 13.4085589293

Ce -3.8155042953 6.6031322540 13.3901752923

Ce -5.7058806013 9.9401190035 13.4122107111

Ce -7.6484103485 13.2601202002 13.4244028596

Ce -9.5678654580 16.5658788394 13.4149190350

Ce 3.8731976178 -0.0234063615 13.4636942926

Ce 1.9365887580 3.2527505567 13.4675196478

Ce -0.0306884489 6.6000202292 13.4734444765

Ce -2.0017519712 9.9279631976 13.4510834935

Ce -3.8625603151 13.2793463542 13.4713830273

Ce -5.8031309286 16.5657736088 13.4271551049

Ce 7.7000100948 0.0449621920 13.4731506729

Ce 5.7883631833 3.2567087706 13.4845567125

Ce 3.8865031358 6.5548313149 13.4908388466

Ce 1.8316258973 9.9452115217 13.5272764816

Ce -0.0870861481 13.1197178869 13.4841157097

Ce -2.1086022524 16.7173756308 13.3989661412

Ce 11.5544251617 0.0468158964 13.4967821889

Ce 9.6311892761 3.3122407930 13.4689324211

Ce 7.7844808906 6.6111542763 13.5193965299

Ce 5.9109110628 10.0226510835 13.3915064508

Ce 3.7845779937 13.2156913187 13.4893468664

Ce 1.9396896824 16.6712954807 13.4357047351

Ce 15.3680662979 0.0618569494 13.5125078760

Ce 13.4477149647 3.3337059094 13.4753066111

Ce 11.5622803591 6.6545199367 13.4966155421

Ce 9.7178492484 10.0204038528 13.5407942505

Ce 7.6809862015 13.3673952066 13.5629936832

Ce 5.8130427419 16.6603603185 13.6122219868

Ce 1.9560823505 1.0959511032 10.2741533699

Ce 0.0333105953 4.4135288720 10.2844934786

Ce -1.8918434722 7.7319774364 10.2610276913

Ce -3.8133584297 11.0482146914 10.2524318766

Ce -5.7495756992 14.3620955182 10.2507672254

Ce -7.6769114742 17.6657369396 10.2285556903

Ce 5.7827362244 1.1086888430 10.2739077788

Ce 3.8630417043 4.4248562227 10.2956272105

Ce 1.9325923132 7.7414827442 10.3294419232

Ce -0.0006848233 11.0506004787 10.2626985845

Ce -1.9209122513 14.3772017861 10.2983182782

Ce -3.8731806728 17.6845564511 10.2105272050

Ce 9.6082591473 1.1149018736 10.2928871481

Ce 7.7028209662 4.4335243418 10.3060780213

Ce 5.7689837630 7.7489324926 10.2980918679

Ce 3.8126206352 11.0523313114 10.2957722775

Ce 1.8909845637 14.3711257752 10.3159541825

Ce -0.0291945967 17.6853586621 10.2678269741

Ce 13.4473560302 1.1225778192 10.3024753911

Ce 11.5302664458 4.4461422369 10.3147010721

Ce 9.6167869330 7.7776241152 10.3376044602

Ce 7.6985681040 11.1049459833 10.2885763560

Ce 5.7416751698 14.3984614281 10.3506552594

Ce 3.8364407472 17.6901806302 10.2942236446

Ce 17.2871213340 1.1427112645 10.3225998288

Ce 15.3672129182 4.4547012668 10.3142345894

Ce 13.4522374546 7.7770743955 10.3155445621

Ce 11.5331557946 11.0957252916 10.3206361793

Ce 9.5884316839 14.4068620980 10.3268770826

Ce 7.6495598459 17.7103157211 10.3160244995

Ce 0.0214642158 2.2057733101 7.0453255198

Ce -1.9021393795 5.5167637064 7.0425804365

Ce -3.8294451363 8.8332595645 7.0283779645

Ce -5.7597165028 12.1469967673 7.0184130335

Ce -7.6892337108 15.4589319131 7.0068034637

Ce -9.6168435593 18.7680596799 7.0086698574

Ce 3.8455749532 2.2165637567 7.0698425828

Ce 1.9244579781 5.5341463890 7.0786042305

Ce -0.0000308020 8.8410117313 7.0562156548

Ce -1.9371206884 12.1623127620 7.0418566570

Ce -3.8629707548 15.4663263715 7.0223342762

Ce -5.7942118276 18.7834692385 7.0032947410

Ce 7.6857211759 2.2267951143 7.0702250546

Ce 5.7763746862 5.5401597572 7.0858976078

Ce 3.8336265534 8.8556158532 7.0804569069

Ce 1.8990801497 12.1739970210 7.0696340989

Ce -0.0317738542 15.4798927757 7.0555916533

Ce -1.9561682051 18.7922378633 7.0220549930

Ce 11.5222605669 2.2353557227 7.0790763661

Ce 9.6095076603 5.5529548934 7.0972739358

Ce 7.6851061190 8.8652830081 7.0971313630

Ce 5.7416130963 12.1862733757 7.1018146772

Ce 3.8208333976 15.4845000362 7.0977597609

Ce 1.8909693993 18.7985006140 7.0601886632

Ce 15.3579694815 2.2443972097 7.0923459176

Ce 13.4336389520 5.5625369133 7.0991234169

Ce 11.5056405658 8.8775367551 7.1045953464

Ce 9.5812463617 12.1986994517 7.0931186450

Ce 7.6490082209 15.5010925450 7.1034503966

Ce 5.7287389421 18.8110109404 7.0758561444

Au -0.7095120718 6.3498906220 17.1473736569

Au 6.6194023333 7.4147374757 18.8329474033

Au 3.2032432243 15.3335518067 16.6534253510

Au 0.4809982372 11.4095978338 20.9724849448

Au -0.3213090503 10.6651398730 18.2327713488

Au 4.9214383582 9.6949545749 18.5231222035

Au 3.6825908839 8.7388886195 15.9520156529

Au 2.3624241389 6.0467950281 21.0507211173

Au 2.1917192969 6.1944385645 16.4997228171

Au 5.2431161115 6.1404841291 16.5153129748

Au 3.7520210270 12.4068737329 18.5348507371

Au 3.9738084035 8.8560663962 21.0355112982

Au 6.6339740774 8.8945441297 16.4204561300

Au 5.0765404679 11.3346611770 16.3384313906

Au 1.1851056678 7.2427415048 18.9452610791

Au 2.2010091217 10.1283997882 19.2926054955

Au 1.9906034729 11.1950118331 16.6662719810

Au 0.7480713268 8.6330841769 16.5632133823

Au 3.9134320987 6.9988495129 18.7147171211

Au 0.9825483303 8.6588842616 21.3573168013

C -2.5911116554 5.7036734463 17.4453910534

O -3.6793097147 5.7996368132 17.8032362025

C 8.1818967688 7.1718333188 20.0276535271

O 8.9431392290 7.3112911773 20.8773701330

C 2.5136059869 15.5731299201 18.4257568757

O 2.0288548438 15.8442477029 19.4310459150

C -0.4506855477 8.0639818454 22.6625453007

O -1.4836926245 7.6518417391 22.9636165482

C 5.0859607296 8.4728489055 22.6832788144

O 6.0178556696 8.3365133394 23.3457115229

C -0.7605051922 12.4371153172 22.1541570547

O -1.6514914146 12.5209103269 22.8779779340

C 2.5763521356 4.7475298370 22.5822315167

O 2.2060836634 4.0474108773 23.4168945492

C -2.3266676873 10.4968648117 18.1554161878

O -3.3280301419 9.9365948156 18.0751627392

(7) O_2_ adsorption at the oxygen defect (Cofig. viii)

O 0.007260235 -0.000642861 6.229377129

O -1.910080747 3.322823815 6.230106722

O -3.826388856 6.637017015 6.231582900

O -5.746184568 9.954319967 6.227488028

O -7.663628782 13.273173273 6.226681531

O -9.580266107 16.593674277 6.227574960

O 3.823941531 0.006763026 6.227883314

O 1.914194081 3.322392453 6.278525118

O -0.001881605 6.639239877 6.279742097

O -1.915671380 9.955641772 6.232056041

O -3.836431478 13.276924873 6.225726128

O -5.757929493 16.595174851 6.222601579

O 7.642977785 0.005145286 6.224793088

O 5.779973692 3.325662521 6.280703968

O 3.872586565 6.639812260 6.288700612

O 1.941063144 9.954342303 6.249983824

O -0.011971185 13.275273784 6.236088109

O -1.932291714 16.591131522 6.225472920

O 11.528057851 0.003437394 6.231422748

O 9.612387293 3.323054860 6.231153865

O 7.696053323 6.637076580 6.286848689

O 5.776415555 9.957683039 6.290849751

O 3.859869412 13.276088578 6.291520728

O 1.947156602 16.593252981 6.286685641

O 15.345315267 -0.003167084 6.231443070

O 13.431293171 3.319380303 6.230819162

O 11.517902624 6.637469686 6.283015704

O 9.599880403 9.956848646 6.283956716

O 7.681924387 13.273274256 6.283031333

O 5.764936004 16.592724026 6.283699481

O 0.051036972 2.215027295 9.488970359

O -1.860204425 5.533726308 9.492720880

O -3.778676290 8.853097768 9.484567164

O -5.690659229 12.173142697 9.476569719

O -7.610146928 15.490288192 9.482065034

O -9.526983421 18.808081750 9.485659740

O 3.867577022 2.216330146 9.490086088

O 1.949157704 5.531496863 9.494724329

O 0.037551097 8.846923634 9.490881930

O -1.882339882 12.172095164 9.486267649

O -3.796558205 15.491615097 9.482340910

O -5.713470344 18.807368190 9.422610661

O 7.689223515 2.214267393 9.486041808

O 5.779969658 5.531592397 9.492413299

O 3.856821493 8.846260172 9.491918681

O 1.937284571 12.169319527 9.490102951

O 0.017325780 15.493448294 9.493416989

O -1.895213614 18.813392866 9.417497125

O 11.505093745 2.209582586 9.481415539

O 9.652215498 5.538999255 9.499017216

O 7.743405580 8.847432135 9.501606935

O 5.754762295 12.168153002 9.493638404

O 3.868392032 15.425126472 9.497428535

O 1.922811082 18.805210188 9.427161339

O 15.393787789 2.211287442 9.489655712

O 13.476458509 5.532010693 9.490192166

O 11.562487270 8.844931522 9.490415700

O 9.644742363 12.168779725 9.492365809

O 7.726602451 15.488683265 9.495635601

O 5.809504177 18.813379786 9.489457227

O 1.916259377 1.106352867 3.129238459

O 0.000000000 4.425411469 3.129238459

O -1.916259377 7.744470070 3.129238459

O -3.832518754 11.063528672 3.129238459

O -5.748778131 14.382587273 3.129238459

O -7.665037508 17.701645875 3.129238459

O 5.748778131 1.106352867 3.129238459

O 3.832518754 4.425411469 3.129238459

O 1.916259377 7.744470070 3.129238459

O 0.000000000 11.063528672 3.129238459

O -1.916259377 14.382587273 3.129238459

O -3.832518754 17.701645875 3.129238459

O 9.581296885 1.106352867 3.129238459

O 7.665037508 4.425411469 3.129238459

O 5.748778131 7.744470070 3.129238459

O 3.832518754 11.063528672 3.129238459

O 1.916259377 14.382587273 3.129238459

O 0.000000000 17.701645875 3.129238459

O 13.413815639 1.106352867 3.129238459

O 11.497556262 4.425411469 3.129238459

O 9.581296885 7.744470070 3.129238459

O 7.665037508 11.063528672 3.129238459

O 5.748778131 14.382587273 3.129238459

O 3.832518754 17.701645875 3.129238459

O 17.246334393 1.106352867 3.129238459

O 15.330075016 4.425411469 3.129238459

O 13.413815639 7.744470070 3.129238459

O 11.497556262 11.063528672 3.129238459

O 9.581296885 14.382587273 3.129238459

O 7.665037508 17.701645875 3.129238459

O 2.002579900 1.103154060 12.615526441

O 0.024360463 4.424465018 12.672910299

O -1.892662111 7.738456365 12.617268625

O -3.814352072 11.063034155 12.601420142

O -5.714241252 14.391344501 12.607903966

O -7.574789616 17.715807695 12.605878194

O 5.831186859 1.107720913 12.673751434

O 3.921569633 4.400423736 12.682323910

O 2.045607762 7.813816019 12.779536131

O 0.043203705 10.995999660 12.678093410

O -1.875374856 14.462634912 12.687010094

O -3.792533026 17.762513130 12.609969975

O 9.649459495 1.108152028 12.612933494

O 7.729448428 4.423697730 12.679286683

O 5.751147987 7.746106136 12.777311445

O 3.823244498 10.917044203 12.773951806

O 2.031774556 14.455459888 12.767621155

O 0.058062032 17.699098513 12.681118942

O 13.464280994 1.112932180 12.608325306

O 11.552677512 4.421404627 12.673867537

O 9.639817020 7.747557222 12.681247312

O 7.769684555 10.992115873 12.684583841

O 5.704815789 14.370800510 12.688063683

O 3.806854819 17.689764558 12.696583624

O 17.287044780 1.116926963 12.598554046

O 15.371843666 4.422640301 12.612697659

O 13.520856801 7.744421289 12.617004480

O 11.620038403 11.062180427 12.610935495

O 9.717712307 14.393907220 12.611550207

O 7.759278773 17.767892085 12.606774698

O 0.000000000 2.212705734 4.693857689

O -1.916259377 5.531764336 4.693857689

O -3.832518754 8.850822937 4.693857689

O -5.748778131 12.169881539 4.693857689

O -7.665037508 15.488940140 4.693857689

O -9.581296885 18.807998742 4.693857689

O 3.832518754 2.212705734 4.693857689

O 1.916259377 5.531764336 4.693857689

O 0.000000000 8.850822937 4.693857689

O -1.916259377 12.169881539 4.693857689

O -3.832518754 15.488940140 4.693857689

O -5.748778131 18.807998742 4.693857689

O 7.665037508 2.212705734 4.693857689

O 5.748778131 5.531764336 4.693857689

O 3.832518754 8.850822937 4.693857689

O 1.916259377 12.169881539 4.693857689

O 0.000000000 15.488940140 4.693857689

O -1.916259377 18.807998742 4.693857689

O 11.497556262 2.212705734 4.693857689

O 9.581296885 5.531764336 4.693857689

O 7.665037508 8.850822937 4.693857689

O 5.748778131 12.169881539 4.693857689

O 3.832518754 15.488940140 4.693857689

O 1.916259377 18.807998742 4.693857689

O 15.330075016 2.212705734 4.693857689

O 13.413815639 5.531764336 4.693857689

O 11.497556262 8.850822937 4.693857689

O 9.581296885 12.169881539 4.693857689

O 7.665037508 15.488940140 4.693857689

O 5.748778131 18.807998742 4.693857689

O 0.073068086 0.010784093 11.095431210

O -1.840743918 3.320290635 11.099777950

O -3.755018936 6.633886405 11.094568022

O -5.669589579 9.952471845 11.092425497

O -7.583193133 13.270224543 11.092597970

O -9.502265242 16.597485251 11.096236796

O 3.905027326 0.001632392 11.097000785

O 1.977038320 3.324960358 11.158369062

O 0.076651334 6.643342812 11.177887701

O -1.858069525 9.960948129 11.099938244

O -3.764292007 13.279706207 11.103777537

O -5.680776424 16.599370935 11.092458807

O 7.713051265 0.002448988 11.092300611

O 5.803180833 3.316558021 11.106695748

O 3.880637451 6.634776117 11.087172178

O 1.955987981 9.955211566 11.095904651

O 0.049918289 13.273774207 11.101237547

O -1.898489675 16.658089913 11.096451118

O 11.534103677 -0.006797812 11.095687895

O 9.618355983 3.322024255 11.102272861

O 7.700613373 6.640753036 11.174125699

O 5.790489772 9.957109331 11.087088897

O 3.824845482 13.200633113 11.180683839

O 1.945354013 16.597901764 11.080899201

O 15.354756382 -0.013110450 11.094544463

O 13.438082835 3.317812318 11.095654625

O 11.576315033 6.636812643 11.157679665

O 9.679933172 9.958136299 11.106156786

O 7.691479193 13.272796810 11.094534860

O 5.775066220 16.597803276 11.079919097

O 0.051994837 2.208246461 14.279168869

O -1.866400869 5.533974701 14.289637612

O -3.780602860 8.851887227 14.286393551

O -5.655195667 12.226635512 14.282412512

O -7.566873974 15.550011270 14.287888726

O -9.483840031 18.871813455 14.285445500

O 3.876866042 2.205830042 14.287876652

O 1.860500374 5.387389343 14.376969644

O -0.054734239 8.843270215 14.292779942

O -2.023127718 12.091187235 14.296602450

O -3.836700636 15.558410945 14.282251356

O -5.707999142 18.810541437 14.287375201

O 7.765190507 2.208243127 14.287078394

O 5.940776211 5.391621511 14.476343429

O 2.055555139 12.090664004 14.471335456

O -1.897653369 18.816667763 14.283711396

O 11.580761463 2.209360105 14.283384048

O 9.667670187 5.532192831 14.288417234

O 7.881611578 8.774319641 14.470685789

O 5.709744082 12.240351642 14.467752746

O 3.848597462 15.344817357 14.715460980

O 1.961328675 18.894853496 14.288411136

O 15.397283874 2.217688539 14.279297561

O 13.484270004 5.533766784 14.281709130

O 11.645518549 8.850331380 14.280999406

O 9.801438977 12.092510408 14.301760704

O 7.920357567 15.630906738 14.376423426

O 5.820663962 18.961377565 14.290420102

O 1.935186435 1.104669810 7.887396152

O 0.017422892 4.424870641 7.894445406

O -1.899142935 7.745502463 7.886913458

O -3.814427761 11.062735169 7.828774010

O -5.731707778 14.381731602 7.828414093

O -7.651765863 17.699464475 7.826917646

O 5.752290549 1.108958578 7.889544501

O 3.839522258 4.425050950 7.895988215

O 1.923130768 7.744718717 7.897391201

O 0.003843767 11.063036796 7.890139064

O -1.912363243 14.382594824 7.890581486

O -3.829562681 17.699340268 7.822726025

O 9.577491878 1.109280395 7.825340099

O 7.727026468 4.425205342 7.899790607

O 5.805547592 7.746650378 7.908097182

O 3.823980101 11.061221029 7.898754795

O 1.902637772 14.380135901 7.895845905

O -0.009365119 17.697137880 7.828954391

O 13.457966226 1.112767359 7.886504640

O 11.547000113 4.422309622 7.894520640

O 9.631100561 7.743770892 7.898811262

O 7.709563381 11.065470244 7.903399608

O 5.796477439 14.381942737 7.899080717

O 3.879404197 17.699276072 7.901906406

O 17.277954826 1.106720446 7.836567853

O 15.363808981 4.424537420 7.887717027

O 13.451054043 7.744962583 7.888955141

O 11.534240597 11.064517372 7.889124973

O 9.616864514 14.382856108 7.889815092

O 7.700140171 17.703135525 7.888665429

Ce 0.000000000 0.000000000 3.911548074

Ce -1.916259377 3.319058601 3.911548074

Ce -3.832518754 6.638117203 3.911548074

Ce -5.748778131 9.957175804 3.911548074

Ce -7.665037508 13.276234406 3.911548074

Ce -9.581296885 16.595293007 3.911548074

Ce 3.832518754 0.000000000 3.911548074

Ce 1.916259377 3.319058601 3.911548074

Ce 0.000000000 6.638117203 3.911548074

Ce -1.916259377 9.957175804 3.911548074

Ce -3.832518754 13.276234406 3.911548074

Ce -5.748778131 16.595293007 3.911548074

Ce 7.665037508 0.000000000 3.911548074

Ce 5.748778131 3.319058601 3.911548074

Ce 3.832518754 6.638117203 3.911548074

Ce 1.916259377 9.957175804 3.911548074

Ce 0.000000000 13.276234406 3.911548074

Ce -1.916259377 16.595293007 3.911548074

Ce 11.497556262 0.000000000 3.911548074

Ce 9.581296885 3.319058601 3.911548074

Ce 7.665037508 6.638117203 3.911548074

Ce 5.748778131 9.957175804 3.911548074

Ce 3.832518754 13.276234406 3.911548074

Ce 1.916259377 16.595293007 3.911548074

Ce 15.330075016 0.000000000 3.911548074

Ce 13.413815639 3.319058601 3.911548074

Ce 11.497556262 6.638117203 3.911548074

Ce 9.581296885 9.957175804 3.911548074

Ce 7.665037508 13.276234406 3.911548074

Ce 5.748778131 16.595293007 3.911548074

Ce 0.048205162 -0.004390380 13.453802148

Ce -1.869003680 3.286908444 13.434608903

Ce -3.792648928 6.632106982 13.423524649

Ce -5.671866217 9.968324342 13.443068406

Ce -7.573238002 13.295995987 13.446411831

Ce -9.498301984 16.616841541 13.475069969

Ce 3.887746102 -0.021420180 13.467186470

Ce 1.955956069 3.235815617 13.475441930

Ce -0.009094966 6.607308214 13.485714896

Ce -1.960292449 9.966375543 13.448714921

Ce -3.795978162 13.298790671 13.455957978

Ce -5.720345543 16.615045702 13.460071445

Ce 7.724893108 0.004656950 13.457635528

Ce 5.828231810 3.239588115 13.475627061

Ce 3.883230311 6.502349326 13.489967659

Ce 1.837187083 9.991827659 13.432714567

Ce 0.010314225 13.210407874 13.600603742

Ce -1.979609846 16.673978211 13.445018404

Ce 11.569708624 0.009414916 13.456783271

Ce 9.654964128 3.302458024 13.454538564

Ce 7.799282709 6.606610711 13.500797388

Ce 5.931237462 9.993935080 13.488338919

Ce 3.863645639 13.244228333 13.504742287

Ce 1.926581941 16.677943709 13.524295305

Ce 15.396785407 -0.002356780 13.462600527

Ce 13.471066143 3.302407272 13.446771520

Ce 11.589672134 6.633834912 13.469810189

Ce 9.746714899 9.995669876 13.473564693

Ce 7.813183430 13.344153948 13.587322177

Ce 5.893306469 16.607864537 13.504165819

Ce 1.965484970 1.093907624 10.285042222

Ce 0.048984619 4.416949834 10.302545323

Ce -1.865551785 7.751579333 10.281748320

Ce -3.786946497 11.069872653 10.263872248

Ce -5.702846141 14.392156497 10.274221461

Ce -7.620571584 17.694317640 10.272440529

Ce 5.788002859 1.099647494 10.269530507

Ce 3.876911435 4.422411046 10.291667349

Ce 1.968635384 7.745937912 10.307045755

Ce 0.030515615 11.083100166 10.269937161

Ce -1.868554330 14.393261031 10.320812647

Ce -3.805520406 17.708381490 10.241251957

Ce 9.614082050 1.099700741 10.266889349

Ce 7.710884622 4.425164192 10.296676685

Ce 5.789042939 7.753970236 10.330202810

Ce 3.866043869 11.049965387 10.306800861

Ce 1.939087120 14.380020983 10.348228235

Ce 0.037946835 17.701012563 10.267661644

Ce 13.454799573 1.097054461 10.271439813

Ce 11.548298486 4.424722952 10.290435096

Ce 9.644073919 7.757267938 10.305415061

Ce 7.722121746 11.082515202 10.309984370

Ce 5.794078900 14.374425395 10.349876386

Ce 3.872188121 17.676074068 10.312194224

Ce 17.299247182 1.090633756 10.273612225

Ce 15.383620711 4.419507251 10.274523782

Ce 13.478454937 7.748615205 10.277975608

Ce 11.565775250 11.072155599 10.271484752

Ce 9.628981497 14.382840611 10.305514297

Ce 7.716295016 17.695420489 10.293224417

Ce 0.018199841 2.211102458 7.063764431

Ce -1.895614817 5.530559315 7.068449981

Ce -3.814362129 8.851856844 7.052267303

Ce -5.735908053 12.173671260 7.045666270

Ce -7.655300233 15.488991673 7.050444773

Ce -9.569544563 18.803103291 7.056139190

Ce 3.844197179 2.212997463 7.073937698

Ce 1.931233609 5.533595218 7.085267909

Ce 0.015596116 8.852910609 7.070867335

Ce -1.909779846 12.180029736 7.055625875

Ce -3.824332851 15.488142752 7.049394603

Ce -5.747241398 18.805804016 7.039199946

Ce 7.681214304 2.213000796 7.059757693

Ce 5.779680221 5.538597430 7.087818633

Ce 3.857618765 8.855337197 7.081489140

Ce 1.930478264 12.178870626 7.083192974

Ce 0.007341845 15.485944158 7.070486686

Ce -1.911055427 18.805650010 7.037906654

Ce 11.518324130 2.209724845 7.054046858

Ce 9.614524080 5.533722777 7.077155559

Ce 7.697776768 8.853444282 7.093731164

Ce 5.771568038 12.176146984 7.097387594

Ce 3.856230583 15.479370226 7.099286784

Ce 1.938699672 18.798703361 7.060937215

Ce 15.354495583 2.206945800 7.053764582

Ce 13.439301328 5.531168543 7.067102268

Ce 11.520949034 8.852112536 7.073924479

Ce 9.601549982 12.175113232 7.078397982

Ce 7.684428865 15.483856215 7.087204572

Ce 5.772386555 18.797469161 7.068523009

Au -0.668661913 6.347566152 17.143836705

Au 6.644571376 7.377384302 18.860306324

Au 3.323356188 15.353333102 16.669783030

Au 0.519881137 11.407899887 20.944300734

Au -0.245702007 10.638923499 18.189688165

Au 4.967810928 9.659907470 18.500495314

Au 3.716118186 8.743085368 15.901114145

Au 2.418002464 6.004927844 21.002692617

Au 2.271178158 6.148375372 16.518666397

Au 5.281974552 6.144016638 16.470546519

Au 3.783014296 12.367505841 18.562325917

Au 4.063949748 8.764491125 21.010293301

Au 6.699657173 8.852719802 16.423244990

Au 5.170806120 11.355033523 16.383536894

Au 1.231146899 7.256534605 18.933939727

Au 2.257283869 10.102000171 19.315481226

Au 2.089462207 11.110134724 16.656653008

Au 0.807717525 8.587987502 16.522782122

Au 3.958304575 6.965382905 18.696972706

Au 1.061825933 8.667926340 21.377254991

C -2.543518883 5.699417350 17.485165166

O -3.630607037 5.823903827 17.837346491

C 8.246782991 7.171329294 20.009162105

O 9.008366479 7.299963417 20.859979519

C 2.645781380 15.596074810 18.444226596

O 2.118363332 15.852569817 19.431894489

C -0.364240458 8.034597413 22.666408808

O -1.408805647 7.671687065 22.987325732

C 5.157205260 8.442434375 22.685238981

O 6.097492815 8.335579285 23.341283693

C -0.711531703 12.432506689 22.133731678

O -1.562205419 12.542472625 22.899917916

C 2.602799803 4.731519393 22.554468651

O 2.272244593 4.058879681 23.426910646

C -2.252031388 10.500763815 18.156256280

O -3.265976183 9.960359351 18.105659195

O -0.128961297 15.553388985 14.538918343

O 0.911227774 16.084619355 15.447579556

(8) the 2nd CO_2_ (Cofig. xi)

O 0.007119212 -0.000446962 6.229437365

O -1.910136998 3.322805211 6.230319427

O -3.826459646 6.636784899 6.231292912

O -5.745870174 9.953340612 6.226294826

O -7.664735577 13.273674592 6.224987351

O -9.580451752 16.593711037 6.227365494

O 3.823774792 0.006695860 6.228049409

O 1.913135183 3.322536007 6.278885042

O -0.001892628 6.638924463 6.280134675

O -1.914970215 9.954261570 6.229599276

O -3.838728971 13.274486041 6.220986684

O -5.759463379 16.596193353 6.220478889

O 7.642209949 0.005677393 6.223652452

O 5.766134356 3.326657117 6.255587000

O 3.871887640 6.639901314 6.288060747

O 1.944807312 9.952778528 6.247107668

O 0.036667943 13.272233128 6.228476805

O -1.932257376 16.595604627 6.221023472

O 11.528082153 0.004492330 6.230779114

O 9.611823582 3.323212255 6.231101629

O 7.695330353 6.637494578 6.286440448

O 5.776987056 9.957323973 6.291430852

O 3.861611460 13.275889040 6.291261897

O 1.948577378 16.593851995 6.284016772

O 15.345649686 -0.002815254 6.231252923

O 13.430795773 3.319573027 6.231159881

O 11.516505925 6.636841238 6.232933178

O 9.598822744 9.956346035 6.281771283

O 7.681964701 13.273389876 6.282810865

O 5.765308952 16.592919987 6.280820044

O 0.051097349 2.215213650 9.489002892

O -1.861120398 5.533875719 9.493587794

O -3.780983526 8.851592174 9.483134294

O -5.692362049 12.171958844 9.420718416

O -7.609375829 15.491229739 9.481099954

O -9.526927779 18.808534106 9.486167232

O 3.867500151 2.215651082 9.490167284

O 1.949500388 5.531774218 9.495044067

O 0.037223777 8.846667496 9.491845838

O -1.882775568 12.172682404 9.476980768

O -3.795480833 15.490280787 9.420685220

O -5.711212228 18.806158632 9.425789244

O 7.688466394 2.213318757 9.486383122

O 5.776856571 5.531662096 9.492021269

O 3.855907744 8.846012560 9.492480069

O 1.937864630 12.168889986 9.488791061

O 0.019788271 15.492438892 9.482903463

O -1.896099305 18.811375020 9.419731949

O 11.504250090 2.211238404 9.475911614

O 9.589565442 5.534414577 9.492926314

O 7.739123018 8.848045714 9.498896795

O 5.756395590 12.168941933 9.494059810

O 3.869240427 15.428060767 9.497527217

O 1.923707121 18.804464888 9.425300909

O 15.393989320 2.212279583 9.489497917

O 13.474504113 5.532349942 9.490201785

O 11.563088395 8.847561438 9.487259162

O 9.646408324 12.169761347 9.491591299

O 7.727172869 15.489311701 9.496603986

O 5.809295059 18.813695133 9.490044687

O 1.916259377 1.106352867 3.129238459

O 0.000000000 4.425411469 3.129238459

O -1.916259377 7.744470070 3.129238459

O -3.832518754 11.063528672 3.129238459

O -5.748778131 14.382587273 3.129238459

O -7.665037508 17.701645875 3.129238459

O 5.748778131 1.106352867 3.129238459

O 3.832518754 4.425411469 3.129238459

O 1.916259377 7.744470070 3.129238459

O 0.000000000 11.063528672 3.129238459

O -1.916259377 14.382587273 3.129238459

O -3.832518754 17.701645875 3.129238459

O 9.581296885 1.106352867 3.129238459

O 7.665037508 4.425411469 3.129238459

O 5.748778131 7.744470070 3.129238459

O 3.832518754 11.063528672 3.129238459

O 1.916259377 14.382587273 3.129238459

O 0.000000000 17.701645875 3.129238459

O 13.413815639 1.106352867 3.129238459

O 11.497556262 4.425411469 3.129238459

O 9.581296885 7.744470070 3.129238459

O 7.665037508 11.063528672 3.129238459

O 5.748778131 14.382587273 3.129238459

O 3.832518754 17.701645875 3.129238459

O 17.246334393 1.106352867 3.129238459

O 15.330075016 4.425411469 3.129238459

O 13.413815639 7.744470070 3.129238459

O 11.497556262 11.063528672 3.129238459

O 9.581296885 14.382587273 3.129238459

O 7.665037508 17.701645875 3.129238459

O 2.004965858 1.103035658 12.614614721

O 0.024836213 4.425531749 12.672841955

O -1.891521093 7.740355203 12.617167414

O -3.816291576 11.061438662 12.600419002

O -5.649410698 14.385281126 12.602890077

O -7.565833446 17.706391339 12.610029399

O 5.832154778 1.105785677 12.674013515

O 3.922069772 4.408579197 12.685027027

O 2.045862801 7.815865170 12.781882045

O 0.016623105 11.049113674 12.667643166

O -1.948015001 14.445600047 12.611598872

O -3.763365458 17.710186565 12.607971412

O 9.648333251 1.108336652 12.611337264

O 7.727550120 4.424218867 12.680809282

O 5.741449307 7.744404752 12.781454389

O 3.813940507 10.921435619 12.771970347

O 2.034093030 14.453600423 12.692499400

O 0.035496843 17.768752793 12.603454455

O 13.464610988 1.112458392 12.609573922

O 11.551425377 4.425233653 12.666396689

O 9.633931920 7.744868925 12.678026171

O 7.781522013 10.996673046 12.675682815

O 5.709917068 14.377306971 12.691057307

O 3.803091983 17.696769457 12.692613449

O 17.285941465 1.115790619 12.598955554

O 15.371359310 4.423266058 12.611864782

O 13.453971618 7.742249139 12.611333280

O 11.620739445 11.056931828 12.601351556

O 9.751637467 14.444868430 12.607690921

O 7.756864870 17.768302273 12.613201095

O 0.000000000 2.212705734 4.693857689

O -1.916259377 5.531764336 4.693857689

O -3.832518754 8.850822937 4.693857689

O -5.748778131 12.169881539 4.693857689

O -7.665037508 15.488940140 4.693857689

O -9.581296885 18.807998742 4.693857689

O 3.832518754 2.212705734 4.693857689

O 1.916259377 5.531764336 4.693857689

O 0.000000000 8.850822937 4.693857689

O -1.916259377 12.169881539 4.693857689

O -3.832518754 15.488940140 4.693857689

O -5.748778131 18.807998742 4.693857689

O 7.665037508 2.212705734 4.693857689

O 5.748778131 5.531764336 4.693857689

O 3.832518754 8.850822937 4.693857689

O 1.916259377 12.169881539 4.693857689

O 0.000000000 15.488940140 4.693857689

O -1.916259377 18.807998742 4.693857689

O 11.497556262 2.212705734 4.693857689

O 9.581296885 5.531764336 4.693857689

O 7.665037508 8.850822937 4.693857689

O 5.748778131 12.169881539 4.693857689

O 3.832518754 15.488940140 4.693857689

O 1.916259377 18.807998742 4.693857689

O 15.330075016 2.212705734 4.693857689

O 13.413815639 5.531764336 4.693857689

O 11.497556262 8.850822937 4.693857689

O 9.581296885 12.169881539 4.693857689

O 7.665037508 15.488940140 4.693857689

O 5.748778131 18.807998742 4.693857689

O 0.072767591 0.011642387 11.095472969

O -1.840997771 3.320320981 11.099843170

O -3.764370936 6.636937075 11.098162214

O -5.668023268 9.954900589 11.089285854

O -7.575832485 13.271547891 11.090952586

O -9.504021280 16.602548625 11.094396200

O 3.905206840 0.000307510 11.097858445

O 1.977356362 3.325137737 11.159037875

O 0.076164643 6.643158636 11.177204130

O -1.860187364 9.962213639 11.098291221

O -3.772068016 13.271244323 11.085501263

O -5.678244437 16.594761046 11.090256670

O 7.713678894 -0.002439392 11.094517634

O 5.800663455 3.314497520 11.108052111

O 3.881444201 6.635087029 11.090469624

O 1.948196819 9.957956628 11.095997460

O 0.047690485 13.279622955 11.094814987

O -1.868008886 16.605666179 11.084716506

O 11.533644384 0.003987003 11.087155909

O 9.618027887 3.321491515 11.102222231

O 7.695193961 6.642863668 11.176277547

O 5.790538123 9.957655174 11.079791696

O 3.831189088 13.199850764 11.171629289

O 1.945429694 16.600363840 11.094153064

O 15.354082330 -0.010798061 11.094145871

O 13.435409777 3.318364270 11.094503740

O 11.522597393 6.634404880 11.101822763

O 9.680799099 9.957186112 11.100862792

O 7.702973450 13.274770961 11.096613967

O 5.772867788 16.599909957 11.089678879

O 0.052951145 2.209145467 14.278044649

O -1.867994507 5.535935893 14.289455221

O -3.785492292 8.849185306 14.281196685

O -5.689302191 12.168991033 14.284989034

O -7.523005413 15.492336938 14.287499441

O -9.481927185 18.872086309 14.285509380

O 3.877815047 2.205774630 14.287383978

O 1.862847017 5.388953790 14.379988844

O -0.052966157 8.850449656 14.296937323

O -2.019413380 12.098105974 14.297783850

O -3.798881162 15.495400665 14.290864341

O -5.701268714 18.807046279 14.284348922

O 7.765520576 2.207000731 14.286115915

O 5.935095776 5.398815052 14.483829949

O 1.977794138 12.096597088 14.465168582

O -1.894924075 18.819634521 14.284031347

O 11.579387820 2.213387480 14.282386507

O 9.665933678 5.531750721 14.288508479

O 7.878576759 8.776558154 14.473335755

O 5.750839849 12.184540863 14.387538677

O 3.779323603 15.405511826 14.646575237

O 1.951580369 18.873925283 14.289834823

O 15.396417743 2.217445407 14.278544688

O 13.483925841 5.535188830 14.279656256

O 11.575497469 8.849933365 14.283703195

O 9.866119583 12.099257175 14.299685129

O 7.922888823 15.633193209 14.378355644

O 5.818371461 18.965159675 14.294658558

O 1.934813737 1.104768052 7.887013417

O 0.017261952 4.424958948 7.894608812

O -1.899269873 7.745792139 7.886513302

O -3.810214098 11.061406045 7.823961612

O -5.734554413 14.383171186 7.822798377

O -7.651454812 17.699339078 7.827516191

O 5.752495648 1.110514765 7.888654953

O 3.839765097 4.424674097 7.895779692

O 1.923285222 7.744655682 7.897842939

O 0.003867505 11.062907495 7.888085155

O -1.906789134 14.378615246 7.822017907

O -3.829110869 17.702603840 7.820499302

O 9.575554681 1.109233919 7.824233398

O 7.717920799 4.420729615 7.892624985

O 5.805375444 7.745676620 7.907957305

O 3.826044038 11.060264051 7.899288895

O 1.906568991 14.381306125 7.894705687

O -0.010675568 17.698588317 7.821492532

O 13.457669409 1.113238807 7.885634293

O 11.543941402 4.427392904 7.894370258

O 9.633139235 7.742658293 7.896043175

O 7.710677969 11.065640376 7.902525262

O 5.797008204 14.382557932 7.898848011

O 3.880436749 17.699326250 7.901124874

O 17.277863770 1.106728271 7.837183741

O 15.363016308 4.425161009 7.886773343

O 13.448135221 7.743177678 7.884415367

O 11.530657049 11.061968215 7.882882043

O 9.617123645 14.382930566 7.890118638

O 7.699529265 17.702881068 7.889223409

Ce 0.000000000 0.000000000 3.911548074

Ce -1.916259377 3.319058601 3.911548074

Ce -3.832518754 6.638117203 3.911548074

Ce -5.748778131 9.957175804 3.911548074

Ce -7.665037508 13.276234406 3.911548074

Ce -9.581296885 16.595293007 3.911548074

Ce 3.832518754 0.000000000 3.911548074

Ce 1.916259377 3.319058601 3.911548074

Ce 0.000000000 6.638117203 3.911548074

Ce -1.916259377 9.957175804 3.911548074

Ce -3.832518754 13.276234406 3.911548074

Ce -5.748778131 16.595293007 3.911548074

Ce 7.665037508 0.000000000 3.911548074

Ce 5.748778131 3.319058601 3.911548074

Ce 3.832518754 6.638117203 3.911548074

Ce 1.916259377 9.957175804 3.911548074

Ce 0.000000000 13.276234406 3.911548074

Ce -1.916259377 16.595293007 3.911548074

Ce 11.497556262 0.000000000 3.911548074

Ce 9.581296885 3.319058601 3.911548074

Ce 7.665037508 6.638117203 3.911548074

Ce 5.748778131 9.957175804 3.911548074

Ce 3.832518754 13.276234406 3.911548074

Ce 1.916259377 16.595293007 3.911548074

Ce 15.330075016 0.000000000 3.911548074

Ce 13.413815639 3.319058601 3.911548074

Ce 11.497556262 6.638117203 3.911548074

Ce 9.581296885 9.957175804 3.911548074

Ce 7.665037508 13.276234406 3.911548074

Ce 5.748778131 16.595293007 3.911548074

Ce 0.053922481 -0.002604273 13.446811169

Ce -1.871670571 3.287021980 13.433375143

Ce -3.804639637 6.639442408 13.420058065

Ce -5.688555549 9.973234796 13.441105132

Ce -7.536684379 13.295687832 13.449992962

Ce -9.497384948 16.621763365 13.473705240

Ce 3.896764228 -0.029663289 13.464353095

Ce 1.957927207 3.231984048 13.472198756

Ce -0.015698893 6.609749266 13.474317315

Ce -1.961014683 9.994312893 13.445681603

Ce -3.783916320 13.294270266 13.450279719

Ce -5.682066831 16.588180297 13.459688503

Ce 7.736715825 -0.028436938 13.456133044

Ce 5.833632084 3.228308328 13.473975949

Ce 3.882564517 6.504753437 13.488612829

Ce 1.805162146 10.029604490 13.414422449

Ce 0.006870922 13.344699586 13.575535222

Ce -1.892958128 16.624247113 13.450726506

Ce 11.554625483 0.017318200 13.446029395

Ce 9.649455035 3.299084419 13.455142262

Ce 7.794802129 6.610260438 13.497053406

Ce 5.915190828 10.020333815 13.427292927

Ce 3.887030145 13.283460786 13.470980266

Ce 1.887283262 16.661575426 13.476686647

Ce 15.385056084 -0.000291007 13.456290182

Ce 13.468567866 3.307814179 13.444607897

Ce 11.565734988 6.632170001 13.455095967

Ce 9.751065521 9.987194593 13.468407975

Ce 7.899080070 13.344368619 13.582933930

Ce 5.854525184 16.649074425 13.586798571

Ce 1.966311476 1.090859723 10.281029557

Ce 0.047270806 4.415820360 10.300434592

Ce -1.869657359 7.754407689 10.281910653

Ce -3.790251346 11.071879680 10.254813974

Ce -5.695055251 14.386208056 10.256800648

Ce -7.617979167 17.694076166 10.274573201

Ce 5.790010245 1.095413779 10.270544627

Ce 3.875369300 4.419082413 10.290713065

Ce 1.966390964 7.747384497 10.308399750

Ce 0.023221460 11.090886984 10.273393150

Ce -1.873983724 14.388668878 10.292976646

Ce -3.794741619 17.694490947 10.250339286

Ce 9.614997756 1.100163157 10.264302385

Ce 7.704502955 4.422989799 10.294923971

Ce 5.787334546 7.747488269 10.319157495

Ce 3.862584219 11.055185509 10.294798716

Ce 1.936404546 14.377215860 10.322093632

Ce 0.031190919 17.705264748 10.254069596

Ce 13.450875834 1.095794816 10.269254042

Ce 11.535706654 4.425495768 10.281464695

Ce 9.631276911 7.757068769 10.293530589

Ce 7.730630380 11.089182508 10.301824307

Ce 5.799528167 14.384400712 10.365014713

Ce 3.882601780 17.676356498 10.315626566

Ce 17.297749319 1.091717977 10.273275139

Ce 15.378559431 4.419505663 10.273992658

Ce 13.464700215 7.748804463 10.269183215

Ce 11.566186908 11.070643127 10.265095918

Ce 9.636299179 14.390539754 10.299250284

Ce 7.711179054 17.691711773 10.304128049

Ce 0.017328950 2.210068221 7.062803664

Ce -1.896084709 5.531074945 7.068493161

Ce -3.813404609 8.853781371 7.051230347

Ce -5.735025735 12.174374707 7.039457946

Ce -7.654005955 15.489381550 7.048281258

Ce -9.569879921 18.801199322 7.058388298

Ce 3.842503598 2.210838392 7.069751770

Ce 1.930161942 5.533424415 7.084739487

Ce 0.015938616 8.853822487 7.072442901

Ce -1.904465831 12.179351950 7.045249696

Ce -3.825719001 15.490201591 7.035142556

Ce -5.745332012 18.804460072 7.042021567

Ce 7.677842734 2.212386759 7.056460108

Ce 5.776793337 5.535285457 7.081987494

Ce 3.857120412 8.854911517 7.079296148

Ce 1.933430933 12.178105809 7.076846432

Ce 0.012248545 15.487036028 7.060659235

Ce -1.914178791 18.803374722 7.034769628

Ce 11.517142934 2.206825236 7.051949913

Ce 9.607468830 5.532599521 7.069768440

Ce 7.696477063 8.855281658 7.090558147

Ce 5.773215889 12.180444561 7.099245532

Ce 3.859219428 15.480008426 7.099129053

Ce 1.939563947 18.798571080 7.061469518

Ce 15.354517405 2.206362501 7.053785253

Ce 13.438651611 5.531501175 7.058938425

Ce 11.520638594 8.853709120 7.065131434

Ce 9.604023221 12.175850560 7.075137685

Ce 7.683444922 15.484943758 7.087515284

Ce 5.772989549 18.796822964 7.068422855

Au -0.689990836 6.509817000 17.139416870

Au 6.711115737 7.401276668 18.763881989

Au 3.751966977 14.209365041 16.473111914

Au 0.546249101 11.381978519 20.998706199

Au -0.256518780 10.702539777 18.233765497

Au 5.000242255 9.757486048 18.493147549

Au 3.738671411 8.831130960 15.942398688

Au 2.427494324 6.091018857 21.051953797

Au 2.260614341 6.291889163 16.454634730

Au 5.343226537 6.234774148 16.457501160

Au 3.800263279 12.507801550 18.572098317

Au 4.060000672 8.960128995 21.024005400

Au 6.705745813 8.980412622 16.403574866

Au 5.124201294 11.469346410 16.344854657

Au 1.262099662 7.313044619 18.928165831

Au 2.279318367 10.168653571 19.260970390

Au 2.043285348 11.288866170 16.639672070

Au 0.840172897 8.716730737 16.531556812

Au 4.000422513 7.043022103 18.695481260

Au 1.060793774 8.662804376 21.369345186

C -2.540934529 5.784109649 17.472747767

O -3.629292213 5.824569206 17.837281223

C 8.263478886 7.223730505 19.987020790

O 9.007103578 7.299998044 20.858951649

C 0.346182677 15.487895681 18.596600992

O 1.048504111 15.420815609 19.535550107

C -0.357041006 8.067773567 22.720288902

O -1.407143692 7.673694774 22.986426458

C 5.189369011 8.508523564 22.640422257

O 6.091477290 8.337413799 23.334974309

C -0.691621315 12.446040815 22.154451194

O -1.561824772 12.543677600 22.900474060

C 2.618004025 4.752018583 22.566364988

O 2.273642288 4.063189066 23.420813493

C -2.266914672 10.528488290 18.173210614

O -3.264511225 9.961191583 18.106033896

O -0.009595295 15.701476347 14.299733257

O -0.353740865 15.560222375 17.657739113
